# Candidate Genes for Age at Menarche Are Associated With Uterine Leiomyoma

**DOI:** 10.3389/fgene.2020.512940

**Published:** 2021-01-22

**Authors:** Irina Ponomarenko, Evgeny Reshetnikov, Alexey Polonikov, Irina Verzilina, Inna Sorokina, Anna Yermachenko, Volodymyr Dvornyk, Mikhail Churnosov

**Affiliations:** ^1^Department of Medical Biological Disciplines, Belgorod State University, Belgorod, Russia; ^2^Department of Biology, Medical Genetics and Ecology, Kursk State Medical University, Kursk, Russia; ^3^Department of Social Epidemiology, Pierre Louis Institute of Epidemiology and Public Health, Sorbonne Universités, Paris, France; ^4^Department of Life Sciences, College of Science and General Studies, Alfaisal University, Riyadh, Saudi Arabia

**Keywords:** age at menarche, association, gene–environment interactions, gene–gene interaction, single nucleotide polymorphism, uterine leiomyoma

## Abstract

Age at menarche (AAM) is an important marker of the pubertal development and function of the hypothalamic–pituitary–ovarian system. It was reported as a possible factor for a risk of uterine leiomyoma (UL). However, while more than 350 loci for AAM have been determined by genome-wide association studies (GWASs) to date, no studies of these loci for their association with UL have been conducted so far. In this study, we analyzed 52 candidate loci for AAM for possible association with UL in a sample of 569 patients and 981 controls. The results of the study suggested that 23 out of the 52 studied polymorphisms had association with UL. Locus rs7759938 *LIN28B* was individually associated with the disease according to the dominant model. Twenty loci were associated with UL within 11 most significant models of intergenic interactions. Nine loci involved in 16 most significant models of interactions between single-nucleotide polymorphism (SNP), induced abortions, and chronic endometritis were associated with UL. Among the 23 loci associated with UL, 16 manifested association also with either AAM (7 SNPs) or height and/or body mass index (BMI) (13 SNPs). The above 23 SNPs and 514 SNPs linked to them have non-synonymous, regulatory, and expression quantitative trait locus (eQTL) significance for 35 genes, which play roles in the pathways related to development of the female reproductive organs and hormone-mediated signaling [false discovery rate (FDR) ≤ 0.05]. This is the first study reporting associations of candidate genes for AAM with UL.

## Introduction

Uterine leiomyoma (UL), commonly known as fibroids, is a benign tumor of smooth muscle in the uterus (Wise and Laughlin-Tommaso, 2016; Stewart et al., 2017). The symptoms include excessive menstrual bleeding, abdominal pain, and pregnancy complications (Zimmermann et al., 2012; Stewart et al., 2017). About 30 percent of reproductive-age women are diagnosed with UL, which is considered as a major factor of gynecologic morbidity (Wise et al., 2004), one of the primary causes of hospitalizations for gynecological disorders, and the most frequent reason for hysterectomy in the United States (Wise and Laughlin-Tommaso, 2016). ULs bear a substantial economic burden: the annual cost of treating this disorder has been estimated to be about $34 billion in the United States alone, which exceeded the combined cost of treating breast and colon cancers (Cardozo et al., 2012).

The biological mechanisms of UL development are not well understood (Rafnar et al., 2018). Family history is an important risk factor for UL (Commandeur et al., 2015; Wise and Laughlin-Tommaso, 2016). A risk to develop the disease is 2.5-fold greater for first-degree relatives of affected women than the population average (Vikhlyaeva et al., 1995), and the concordance among monozygotic twins is almost twice that of dizygotic twins (Luoto et al., 2000). Heritability estimates of UL from twin studies vary from 26 to 69% in European populations (Snieder et al., 1998; Luoto et al., 2000). More estimates come from recent genome-wide association studies (GWASs). Cha et al. (2011) reported candidate loci for UL in chromosome regions 10q24.33 (*OBFC1*), 11p15.5 (*BET1L*), and 22q13.1 (*TNRC6B*) in a cohort of Japanese patients. One more locus, 17q25.3 (*FASN*, *CCDC57*, and *SLC16A3*), was suggested by the genome-wide linkage and follow-up association studies in a meta-analysis of white women (Eggert et al., 2012). The 11p15.5 (*BET1L*) locus was later replicated in a sample of Caucasians (Edwards et al., 2013). The 22q13.1 (*TNRC6B*) locus was replicated in Caucasian, American, and Saudi Arabian cohorts (Edwards et al., 2013; Aissani et al., 2015; Bondagji et al., 2017). However, none of the above loci was replicated in African–American women (Wise et al., 2012). A GWAS of a risk for leiomyoma in African–American women suggested a locus on 22q13.1 (*CYTH4*) (Hellwege et al., 2017). In 2018, two teams presented results of the first GWAS of UL in European populations (Rafnar et al., 2018; Välimäki et al., 2018) reporting 21 and 22 loci associated with the increased risk of UL.

Age at menarche (AAM) has been suggested as a risk factor for UL (Wise et al., 2004; Terry et al., 2010; Velez Edwards et al., 2013; Wise and Laughlin-Tommaso, 2016). Women with an early AAM have, on average, longer period of menstrual cycling in their life and thus a greater lifelong exposure to estrogens, which may promote growth of UL (Wise et al., 2004). Cell division rate in the myometrium is the highest during the luteal phase of the menstrual cycle; therefore, a longer history of cycling may increase a risk of UL (Wise and Laughlin-Tommaso, 2016). On the other hand, some studies reported no significant association between age of menarche and UL (Samadi et al., 1996; Zimmermann et al., 2012).

The significantly different heritability estimates for UL [from 26 to 69% according to twin studies (Snieder et al., 1998; Luoto et al., 2000) and only 13% according to GWAS data single-nucleotide polymorphism (SNP) heritability (Rafnar et al., 2018)] bring about a problem of so-called “missing heritability.” Studies of candidate genes associated with specific risk factors for UL [e.g., AAM and body mass index (BMI)] may help to solve this problem. For example, more than 350 loci have been reported by GWAS for association with AAM (Day et al., 2017). There are data that candidate genes for AAM (e.g., *FTO*, *LIN28B*, *MAP2K5*, *TNNI3K*, *GPRC5B*, and *FANCL*) may also contribute to various anthropometric traits (e.g., BMI, height, and others) (Ong et al., 2009; Elks et al., 2010; Fernandez-Rhodes et al., 2013; Perry et al., 2014), while some of these traits (e.g., an increased BMI) may be risk factors for UL (Luoto et al., 2000; Wise and Laughlin-Tommaso, 2016; McWilliams and Chennathukuzhi, 2017). However, there have been no studies on the possible association of the candidate genes for AAM with a risk of UL.

The purpose of the present study was to analyze 52 candidate loci for menarcheal age for their possible association with UL. Recently, we studied association of these loci with some phenotypic traits (including AAM, height, and BMI) in the same sample of Caucasian females (Ponomarenko et al., 2019). Using the same sample for the analysis of different traits eliminates between-sample heterogeneity and makes comparison and interpretation of the results more meaningful and robust.

## Materials and Methods

### Study Participants

The study protocol was approved by the Regional Ethics Committee of Belgorod State University. All participants signed informed consent documents before entering the study. The participants were recruited through the Perinatal Center of the Belgorod Regional Clinical Hospital of St. Joasaph during 2008–2013. All patients with UL underwent hysterectomy followed by morphological verification of the diagnosis. The control group included women without clinical (asymptomatic women) and ultrasound signs of benign proliferative diseases of the reproductive organs. They were enrolled during regular medical examinations at the above Perinatal Center. A total of 1,620 women were recruited: 600 patients with UL (ICD-10 code D25) and 1,020 controls.

The participants were enrolled under the following exclusion criteria: self-declared non-Russian descent, a birthplace outside of Central Russia (Sorokina et al., 2018), chronic severe disorders of the vital organs (heart, respiratory, or renal failure), cancers of pelvis and breast, and severe autoimmune disorders.

The following data were collected for each participant: physical characteristics (height, weight, and BMI), AAM (determined as described elsewhere; Ponomarenko et al., 2019), characteristics of the menstrual cycle (length and duration of menses), other data about reproductive health (number of pregnancies and childbirths, age at first birth, time since last birth, spontaneous and induced abortions, and infertility), marital status, family history of UL, use of oral contraceptives and age at first oral contraceptive use, and smoking and alcohol use. The participants were also examined for the presence of gynecological disorders (endometrial hyperplasia, endometriosis, adenomyosis, etc.).

### Blood Sample Collection and DNA Handling

The phlebotomy was performed by a certified nurse. DNA extraction from buffy coat was performed according to the protocol used in our previous gene association studies (Ponomarenko et al., 2019).

### Single-Nucleotide Polymorphism Selection

The SNPs for the study were selected according to the criteria and procedure and using the online tools described elsewhere (Ponomarenko, 2018; Ponomarenko et al., 2019). Briefly, the criteria included the following: (1) associations with AAM or traits, which have common biological pathways with menarche (anthropometric characteristics, obesity, etc.), (2) effect on gene expression (eSNP), (3) regulatory potential (regSNP), (4) tag value (tagSNP), and (5) minor allele fraction (MAF) > 5%.

The regulatory potential of the SNPs and effect on gene expression was estimated using the following online tools: HaploReg (v4.1)^1^, RegulomeDB (version 1.1)^2^, rSNPBase^3^, SNPinfo Web Server–SNP Function Prediction (FuncPred)^4^, Blood eQTL browser^5^, and GTEx Portal^6^.

In total, 52 loci were selected for the present study (Supplementary Tables 1–4). All SNPs appeared to have a significant regulatory potential (Supplementary Table 2), 43 SNPs were eSNPs (Supplementary Table 3), and 29 were tagSNPs.

Fourteen loci among the 52 selected showed association with AAM according to the results of GWAS and 28 loci according to the results of gene association studies (Supplementary Table 4). Seventeen SNPs were associated with anthropometric characteristics (Supplementary Table 4). Ten more polymorphisms were not directly associated with AAM but manifested association or tagged with the traits related to menarche [e.g., polycystic ovary syndrome (PCOS), vitamin D metabolism, and physical characteristics; Supplementary Table 4]. These SNPs included rs1884051 *ESR1*, rs3020394 *ESR1*, rs12324955 *FTO*, rs4633 *COMT*, rs222020 *GC*, rs222003 *GC*, rs1544410 *VDR*, rs3756261 *EGF*, rs7766109 *F13A1*, and rs2252673 *INSR*. All these SNPs have a significant regulatory potential, nine of them are eSNPs, and eight are tagSNPs.

Several of the selected loci were previously reported to be associated with AAM (14 SNPs), BMI (15 SNPs), and height (16 SNPs) in the studied sample (Ponomarenko et al., 2019).

### Single-Nucleotide Polymorphism Genotyping and Data Quality Control

DNA samples were genotyped using the Sequenom MassARRAY^®^ iPLEX platform at the Centre of Genomic Sciences (University of Hong Kong). The procedure for DNA sample preparation and data quality control are described elsewhere (Ponomarenko et al., 2019). All DNA samples successfully met the following quality control criteria: call rate > 95%, the success rate of duplicate check > 99.5%, and the success rate of the blank check > 90%. Finally, the proportion of the determined genotypes for the 52 SNPs was 98.84%. The samples with a proportion of determined genotypes < 95% were excluded from the analysis (*n* = 70). Thus, the final study sample included 1,550 participants: 569 women with UL and 981 control subjects.

### Statistical Analysis

Association of clinical anamnestic risk factors with UL was assessed using logistic regression analysis as implemented in the *epicalc* package in the R software environment [version 3.4.0 (2017-04-21)].

The correspondence of the SNPs to the Hardy–Weinberg equilibrium (HWE) was checked using the chi-square test. The logistic regression method was used to analyze associations of the SNPs with UL assuming additive, recessive, and dominant genetic models. To account for possible confounding effects, the following set of covariates was applied: family history of UL, history of infertility, the presence of induced abortions in the anamnesis, and chronic endometritis as qualitative variables (yes/no), whereas age, BMI, and the number of induced abortions in the anamnesis as quantitative variables (value of the trait) (Table 1). The adaptive permutation test was applied to adjust for multiple comparisons (Che et al., 2014). The significance level was set at *p*_*perm*_ < 0.01 (after the Bonferroni correction based on the numbers of genetic models studied).

**TABLE 1:** Participant characteristics from case and control groups.

Parameters	Case group (*n* = 569)	Control group (*n* = 981)	*p*
	$\bar{x}$ ± SD/%(n)	$\bar{x}$ ± SD/%(n)	
Age = years	43.22 ± 8.35	40.73 ± 8.60	**1e-5**
Height = m	1.66 ± 0.06	1.65 ± 0.06	0.24
Weight = kg	76.43 ± 14.35	72.49 ± 13.37	**1e-5**
BMI = kg/m^2^	27.90 ± 5.38	26.66 ± 4.61	**0.0002**
**Ratio of participants to BMI % (n):**			
Underweight (<18.50)	1.58 (9)	1.12 (11)	**1e-5**
Normal weight (18.50–24.99)	31.81 (181)	42.41 (416)	
Overweight (25.00–29.99)	34.27 (195)	30.49 (299)	
Obese (>30.00)	32.34 (184)	25.99 (255)	
Family history (mother had uterine leiomyoma)	35.15 (200)	17.53 (172)	**0.0005**
Married	85.06 (484)	85.93 (843)	0.69
Smoker (yes)	13.71 (78)	17.33 (170)	0.07
Alcohol consumption (≥7 drinks per week)	2.99 (17)	3.06 (30)	0.99
Oral contraceptive use	9.49 (54)	10.09 (99)	0.77
Beginning age of oral contraceptive use (mean; years)	23.43 ± 2.28	23.64 ± 2.36	0.62
**Onset of menarche or first menstrual cycle**			
Age of menarche (years)	13.45 ± 1.31	13.27 ± 1.25	0.31
Ratio of participants at onset of menarche % (*n*):			
Early (<12 years)	4.62 (26)	6.42 (63)	0.36
Average (12–14 years)	80.28 (452)	79.51 (780)	
Late (>14 years)	15.10 (85)	14.07 (138)	
Duration of menstrual bleeding (mean; days)	5.07 ± 1.56	4.94 ± 0.94	0.62
Length of menstrual cycle (mean; days)	28.04 ± 2.15	28.15 ± 2.24	0.78
**Reproductive traits**			
Age at first birth (mean; years)	21.19 ± 2.59	21.71 ± 3.49	0.27
Time elapsed since last birth (mean; years)	15.08 ± 2.28	14.32 ± 2.07	0.24
No. of gravidity (mean)	3.34 ± 2.22	2.45 ± 1.55	**1e-5**
No. of births (mean)	1.46 ± 0.85	1.51 ± 0.67	0.52
No. of spontaneous abortions (mean)	0.26 ± 0.64	0.24 ± 0.51	0.64
No. of induced abortions (mean)	1.59 ± 1.65	0.67 ± 0.99	**1e-5**
No. of induced abortions:	31.81 (181)		
0	23.20 (132)	58.92 (578)	**1e-5**
1	21.62 (123)	23.75 (233)	
2	12.30 (70)	10.40 (102)	
3	11.07 (63)	5.40 (53)	
≥4		1.53 (15)	
History of infertility	13.71 (78)	5.20 (51)	**0.0005**
**Gynecologic pathology**			
Cervical disorders	26.01 (148)	25.08 (246)	0.73
History of sexually transmitted disease	27.06 (154)	26.91 (264)	0.99
Chronic endometriosis	10.02 (57)	5.71 (56)	**0.003**
Chronic inflammation of adnexa	34.62 (197)	31.91 (313)	0.30
Endometrial hyperplasia	47.10 (268)	–	**–**
Endometriosis	36.38 (207)	–	**–**
Adenomyosis	33.92 (193)	–	**–**

*BMI, body mass index. The values of *P* < 0.05 are in bold.*

The given sample size (569 patients with UL and 981 controls) was sufficient to detect differences in allelic frequencies between affected subjects and controls at OR = 1.23–1.57 for the additive model, OR = 1.35–1.60 for the dominant model, and OR = 1.40–8.70 for the recessive model (at 80% power, ɑ= 0.05 for two-sided test).

The haplotype blocks were identified using the algorithm implemented in HaploView v.4.2 (Barrett et al., 2005). The association analyses and permutation test were performed using the respective algorithms implemented in the PLINK v. 2.050 software (Purcell et al., 2007)^7^. The significance level was set at *p*_*perm*_ < 0.05.

The interactions between genes were analyzed assuming the two-, three-, and four-locus models and using the model-based multifactor dimensionality reduction (MB-MDR) method (Calle et al., 2008, 2010; Mahachie et al., 2012) in the *mbmdr* package (version 2.6) in the R software environment [version 3.4.0 (2017-04-21)]. The permutation test was demonstrated to be efficient for analysis of large massifs of GWAS data without reduction of the power (Che et al., 2014). For the permutation test, the following threshold *p*-values (after the Bonferroni correction based on the numbers of combinations studied for 52 SNPs) were adopted for models of gene–gene interactions: *p* < 3.8 ^∗^ 10^–5^ (<0.05/1,326) for two-locus models, *p* < 2.3 ^∗^ 10^–6^ (<0.05/22,100) for three-locus models, and *p* < 1.8 ^∗^ 10^–7^ (<0.05/270,725) for four-locus models. The significance level was set at *p*_*perm*_ ≤ 0.001.

The interactions of the genes with induced abortions and chronic endometritis were analyzed for their possible effect on UL. Induced abortions and chronic endometritis were included in the analysis because (i) they were determined as risk factors for UL in the study sample (Table 1), (ii) induced abortions are a common birth control method of women in Russia as compared with other countries (Sedgh et al., 2007), and (iii) induced abortions are a common cause of post-abortion endometritis and, subsequently, chronic endometritis in Russian women (Douglas et al., 2014). The analysis was performed using MB-MDR with adjustment for covariates (age, BMI, family history of UL, and history of infertility) and multiple comparisons (1,000 permutations) as described above. The permutation test was applied to the selected best models of gene–environment interactions with the significance level of *p* < 1 ^∗^ 10^–19^. The significance level was set at *p*_*perm*_ < 0.001.

The most significant models of gene–gene and gene–environment interactions associated with UL were cross-validated using generalized multifactor dimensionality reduction (GMDR) (Lou et al., 2007; Chen et al., 2011)^8^ and the respective software (Beta 0.9)^9^. The main parameters of the validation were adjusted for covariates and multiple comparisons using the permutation test (1,000 permutations with 10-fold cross-validation, which provided statistical significance at *p*_*perm*_ < 0.001 for a validated model).

The identified interactions and proportion of their contribution to the total variance of the trait were visualized using the MDR method^10^ and the MDR v. 3.0.2 software^11^.

### Functional Single-Nucleotide Polymorphisms

The HaploReg v. 4.1^12^ and the data of the European population from the 1000 Genomes Project Phase were utilized to determine SNPs linked to the UL-associated polymorphisms. Then all these loci were analyzed for their functional significance (non-synonymous SNPs, regulatory potential, and eQTLs).

#### Non-synonymous Single-Nucleotide Polymorphisms

The SIFTonline tool^13^ was used to analyze non-synonymous SNPs and their functional predictions.

#### Regulatory Effects

The regulatory potential of the candidate loci for UL was analyzed using SNP Function Prediction (FuncPred)^14^, rSNPBase^15^, RegulomeDB (version 1.1)^16^, and HaploReg (v4.1).

#### Expression Quantitative Trait Loci

The data from the Blood eQTL browser^17^ were utilized to assess the eQTL (*cis-* and *trans*-eQTL) significance of the studied SNPs in peripheral blood. The eQTL value in other organs and tissues was estimated using the GTEx Portal^18^ (data release V7 updated on 09/05/2017, dbGaP Accession phs000424.v7.p2). The false discovery rate (FDR) ≤ 0.05 was applied.

#### Pathway Analyses

The functional significance of the UL-associated genes in metabolic pathways was analyzed using the Gene Ontology Portal tools (PANTHER Overrepresentation Test accessed on 04/13/2017; PANTHER version 12.0 accessed on 07/10/2017^19^) and the FDR test to correct the results for multiple comparisons. GeneMANIA^20^ (version 3.5.0, accessed on 03/13/2017) and the automatic weighting were used to construct the gene interaction networks. Gene Ontology Portal provides a possibility to evaluate functional significance of genes in various metabolic pathways using seven different data bases (Gene Ontology molecular function, Gene Ontology biological process, PANTHER protein class, PANTHER pathway, PANTHER molecular function, PANTHER biological process, and Reactome pathway) and makes it possible to construct the gene interaction networks based on seven different variants of gene interaction, i.e., physical interactions, predicted interaction, pathways, co-expression, co-localization, genetic interactions, and shared protein domains.

## Results

**FIGURE 1:**
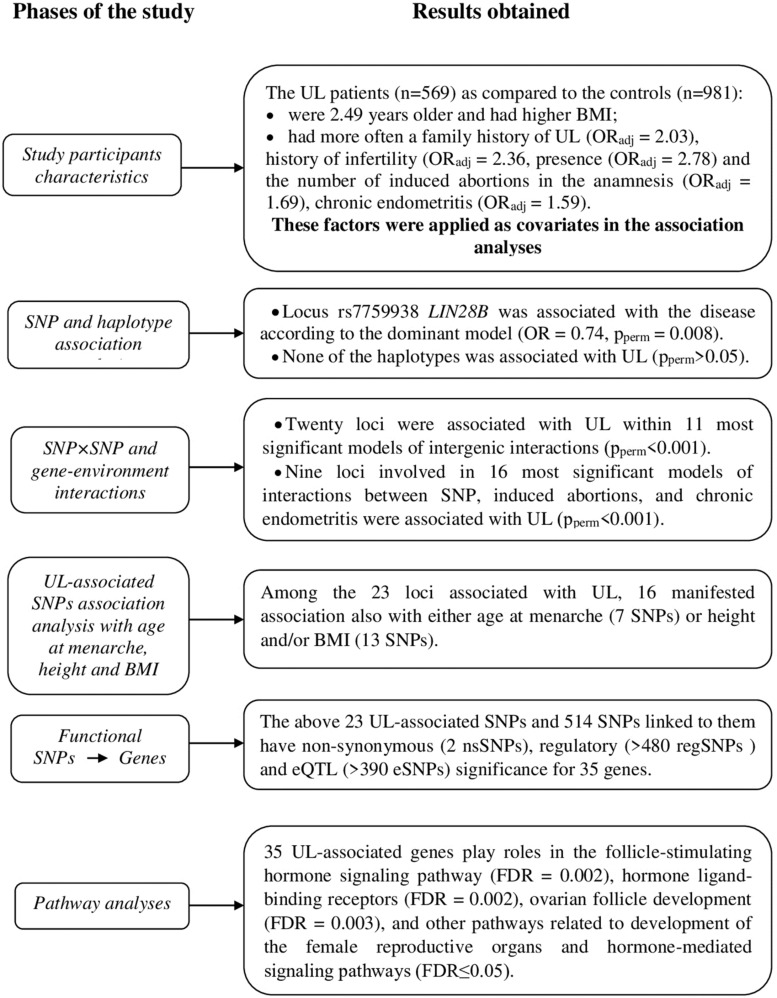
Schematically displayed the phases of the associations study single-nucleotide polymorphism (SNP) candidate genes for age at menarche and uterine leiomyoma (UL) and at each phase at short note the results obtained.

The phases of the study and main results are outlined in Figure 1.

### Study Participants Characteristics

The UL patients (*n* = 569) were 2.49 years older and had higher BMI as compared with the controls (*n* = 981) (*p* < 0.001) (Table 1). They also had more often a family history of UL (adjusted for age and BMI, OR_*adj*_ = 2.03, 95% CI 1.46–2.98, p < 0.001), history of infertility (OR_*adj*_ = 2.36, 95% CI 1.75–3.97, *p* < 0.001), presence (OR_*adj*_ = 2.78, 95% CI 2.23–3.48, *p* < 0.001), and the number of induced abortions in the anamnesis (OR_*adj*_ = 1.69, 95% CI 1.54–1.86, *p* < 0.001), chronic endometritis (OR_*adj*_ = 1.59, 95% CI 1.08–2.35, *p* = 0.02) (Table 1). Therefore, these factors (in addition to age and BMI) were applied as covariates in the association analyses. The higher number of pregnancies in the patients (3.34 vs. 2.45 in cases and controls, respectively) was due to the higher number of the induced abortions (1.59 vs. 0.67 in cases and controls, respectively) in this group as compared with the controls.

### Single-Nucleotide Polymorphism and Haplotype Association Analysis

The data about the analyzed loci are presented in Supplementary Tables 5, 6. All loci had MAF > 5% and were in correspondence with the HWE (*ð*_*bonf*_ > 0.001).

Allele C of the rs7759938 *LIN28B* locus was associated with UL according to the dominant model (OR = 0.74, 95% CI 0.59–0.92, *p* = 0.006, *p*_*perm*_ = 0.008, power = 82.62%) (Table 2). None of the haplotypes was associated with UL (*p*_*perm*_ > 0.05) (Supplementary Table 7).

**TABLE 2:** Associations of the 52 SNPs with uterine leiomyoma.

Chr	SNP	*n*	Additive model				Dominant model				Recessive model			
			OR	95% CI		*p*	OR	95% CI		*p*	OR	95% CI		p
				L95	U95			L95	U95			L95	U95	
1	rs1514175	1,407	0.98	0.83	1.14	0.759	0.86	0.69	1.08	0.200	1.20	0.88	1.63	0.247
1	rs466639	1,410	0.92	0.72	1.17	0.485	0.95	0.73	1.23	0.681	0.46	0.14	1.50	0.196
1	rs7538038	1,411	1.14	0.94	1.37	0.174	1.17	0.94	1.47	0.167	1.16	0.70	1.92	0.570
2	rs713586	1,407	0.92	0.79	1.08	0.313	0.89	0.70	1.13	0.336	0.91	0.68	1.21	0.511
2	rs2164808	1,409	1.04	0.89	1.22	0.603	1.01	0.78	1.30	0.942	1.11	0.85	1.45	0.430
2	rs7589318	1,406	1.01	0.85	1.20	0.920	0.95	0.76	1.18	0.632	1.22	0.84	1.77	0.296
2	rs4374421	1,353	0.91	0.76	1.08	0.271	0.80	0.64	1.01	0.055	1.17	0.80	1.71	0.422
2	rs7579411	1,397	0.88	0.75	1.04	0.128	0.77	0.61	0.98	0.032	0.98	0.74	1.29	0.862
2	rs6729809	1,343	0.94	0.79	1.13	0.528	0.93	0.74	1.17	0.516	0.94	0.63	1.40	0.776
2	rs4953616	1,403	0.98	0.82	1.18	0.831	0.95	0.76	1.18	0.622	1.11	0.71	1.74	0.635
2	rs6732220	1,409	0.93	0.77	1.11	0.406	0.93	0.74	1.16	0.510	0.83	0.51	1.35	0.454
2	rs4953655	1,410	0.89	0.74	1.07	0.209	0.89	0.72	1.12	0.329	0.74	0.44	1.22	0.236
2	rs887912	1,334	0.84	0.70	1.02	0.075	0.81	0.65	1.03	0.081	0.79	0.48	1.30	0.357
2	rs12617311	1,405	0.95	0.80	1.12	0.514	0.87	0.69	1.08	0.200	1.12	0.79	1.59	0.522
3	rs6438424	1,397	0.96	0.82	1.12	0.570	1.00	0.78	1.27	0.970	0.88	0.67	1.15	0.355
4	rs2013573	1,408	0.93	0.76	1.15	0.513	0.92	0.73	1.16	0.455	1.02	0.50	2.06	0.961
4	rs13111134	1,408	0.98	0.81	1.18	0.795	0.93	0.74	1.16	0.504	1.25	0.75	2.08	0.401
4	rs222003	1,410	1.13	0.83	1.53	0.431	1.13	0.82	1.55	0.451	1.42	0.22	9.05	0.711
4	rs222020	1,411	1.20	0.94	1.52	0.144	1.20	0.93	1.56	0.164	1.44	0.54	3.86	0.467
4	rs3756261	1,401	1.25	0.93	1.68	0.132	1.23	0.91	1.67	0.185	3.41	0.57	20.47	0.179
5	rs757647	1,394	0.95	0.79	1.15	0.598	0.92	0.74	1.16	0.484	1.04	0.62	1.75	0.869
6	rs7766109	1,408	1.07	0.91	1.26	0.389	0.98	0.77	1.26	0.883	1.25	0.96	1.63	0.104
6	rs4946651	1,410	0.88	0.75	1.03	0.119	0.80	0.64	1.00	0.055	0.93	0.70	1.25	0.640
6	rs7759938	1,409	0.82	0.68	0.97	0.023	**0.74**	**0.59**	**0.92**	**0.006**	0.93	0.62	1.41	0.738
6	rs314280	1,383	0.93	0.79	1.09	0.351	0.83	0.66	1.04	0.112	1.06	0.78	1.45	0.717
6	rs314276	1,372	0.84	0.71	1.00	0.053	0.78	0.62	0.97	0.026	0.90	0.61	1.32	0.591
6	rs3020394	1,410	0.98	0.83	1.16	0.802	1.02	0.81	1.27	0.892	0.86	0.59	1.26	0.430
6	rs1884051	1,412	0.96	0.81	1.14	0.624	0.99	0.79	1.23	0.914	0.83	0.56	1.23	0.357
6	rs7753051	1,411	0.93	0.79	1.11	0.435	0.86	0.69	1.07	0.169	1.14	0.78	1.69	0.499
7	rs1079866	1,410	1.07	0.87	1.32	0.504	1.10	0.87	1.39	0.432	0.97	0.50	1.91	0.938
8	rs2288696	1,411	0.91	0.75	1.11	0.360	0.92	0.73	1.15	0.452	0.78	0.42	1.43	0.423
9	rs2090409	1,320	0.97	0.82	1.15	0.725	1.03	0.82	1.30	0.804	0.84	0.60	1.17	0.296
9	rs10980926	1,403	0.92	0.78	1.09	0.340	0.83	0.67	1.04	0.105	1.13	0.78	1.64	0.527
9	rs10441737	1,340	0.89	0.75	1.06	0.199	0.81	0.64	1.02	0.068	1.04	0.72	1.51	0.833
11	rs10769908	1,390	1.00	0.85	1.17	0.970	0.92	0.71	1.17	0.488	1.10	0.84	1.43	0.498
11	rs555621	1,407	0.93	0.79	1.09	0.352	0.95	0.76	1.20	0.681	0.82	0.60	1.13	0.223
11	rs11031010	1,393	0.74	0.58	0.95	0.016	0.71	0.54	0.93	0.014	0.73	0.31	1.73	0.471
11	rs1782507	1,403	1.13	0.96	1.33	0.150	1.10	0.87	1.37	0.429	1.34	0.97	1.87	0.080
11	rs6589964	1,407	1.11	0.95	1.29	0.195	1.17	0.91	1.50	0.215	1.12	0.87	1.46	0.385
12	rs1544410	1,404	0.98	0.84	1.15	0.817	0.93	0.74	1.17	0.529	1.07	0.78	1.46	0.674
14	rs999460	1,411	0.91	0.77	1.07	0.258	0.84	0.67	1.05	0.116	1.01	0.72	1.44	0.937
14	rs4986938	1,408	1.13	0.96	1.34	0.146	1.05	0.84	1.32	0.664	1.50	1.07	2.09	0.019
15	rs2241423	1,402	0.96	0.79	1.18	0.722	1.03	0.81	1.30	0.807	0.53	0.26	1.08	0.081
16	rs12444979	1,407	0.92	0.74	1.15	0.448	0.98	0.77	1.26	0.880	0.39	0.17	0.93	0.034
16	rs9939609	1,408	0.96	0.82	1.12	0.584	0.92	0.73	1.16	0.481	0.98	0.74	1.31	0.893
16	rs12324955	1,412	0.91	0.77	1.08	0.291	0.97	0.78	1.21	0.799	0.65	0.43	0.99	0.047
18	rs1398217	1,402	0.96	0.82	1.13	0.621	1.02	0.81	1.29	0.866	0.84	0.62	1.14	0.261
19	rs2252673	1,407	1.11	0.92	1.34	0.279	1.11	0.88	1.39	0.379	1.29	0.77	2.17	0.334
20	rs1073768	1,405	0.99	0.85	1.16	0.906	0.98	0.76	1.26	0.877	1.00	0.76	1.30	0.973
22	rs4633	1,411	1.01	0.87	1.18	0.856	1.16	0.90	1.49	0.264	0.90	0.70	1.16	0.409
23	rs5930973	1,390	1.06	0.75	1.48	0.756	NA	NA	NA	NA	NA	NA	NA	NA
23	rs3092921	1,410	1.02	0.76	1.36	0.922	NA	NA	NA	NA	NA	NA	NA	NA

*All results were obtained after adjustment for covariates. Statistically significant values after adjustment by the adaptive permutation test. ÎR, odds ratio; 95% CI, 95% confidence interval; SNP, single-nucleotide polymorphism. The values of *P* < 0.05 are in bold.*

### Single-Nucleotide Polymorphism × Single-Nucleotide Polymorphism Interactions

In total, 11 most significant two-, three-, and four-locus models of gene–gene interactions associated with UL were determined (Table 3 and Supplementary Table 8). These models included 20 SNPs. Loci of the *FSHB*, *LIN28B*, and *POMC* genes appeared in the largest number of models (seven, six, and five, respectively). Locus rs314276 *LIN28B* contributed to the most significant gene–gene interaction models at all the levels considered. Among the obtained models of SNP–SNP interactions, the most optimal for prediction was rs314276 *LIN28B* × rs1782507 *FSHB* × rs1544410 *VDR* × rs7589318 *POMC* (OR = 2.69, Test. Bal. Acc. 54.70, sensitivity 68.72, specificity 55.05, Table 3). It had the highest values of sensitivity and specificity as compared with the others.

**TABLE 3:** The most significant models of SNP–SNP interactions associated with uterine leiomyoma.

*N*	Models of SNP × SNP interactions	NH	*beta* H	WH	NL	*beta* L	WL	*p*_*perm*_
Two-locus models (*ð* < 3 * 10^–5^)								
1	rs11031010 *FSHB* × rs2241423 *MAP2K5*	1	0.234	4.67	2	–0.718	19.10	< 0.001
2	rs222020 *GC* × rs4374421 *LHCGR*	2	0.288	7.14	2	–0.504	18.33	< 0.001
3	rs314276 *LIN28B* × rs2164808 *POMC*	1	0.460	8.96	2	–0.700	17.53	0.001
Three-locus models (*ð* < 5 * 10^–8^)								
1	rs1782507 *FSHB* × rs1884051 *ESR1* × rs7766109 *F13A1*	4	0.966	39.52	1	–0.444	4.37	< 0.001
2	rs1782507 *FSHB* × rs3020394 *ESR1* × rs7766109 *F13A1*	4	0.927	36.66	1	–0.448	4.58	< 0.001
3	rs11031010 *FSHB* × rs314276 *LIN28B* × rs2164808 *POMC*	4	0.496	20.14	4	–0.827	30.63	< 0.001
4	rs314276 *LIN28B* × rs2164808 *POMC* × rs7753051 *IGF2R*	6	0.621	30.44	4	–0.702	23.45	< 0.001
5	rs11031010 *FSHB* × rs2164808 *POMC* × rs7753051 *IGF2R*	4	0.635	29.87	3	–0.507	14.54	< 0.001
Four-locus models (*ð* < 5 * 10^–13^)								
1	rs314276 *LIN28B* × rs1782507 *FSHB* × rs1544410 *VDR* × rs7589318 *POMC*	11	0.998	55.89	6	–0.967	36.33	< 0.001
2	rs12324955 *FTO* × rs10980926 *ZNF483* × rs555621 *FSHB* × rs4953655 *FSHR*	10	0.868	53.39	5	–0.764	16.10	< 0.001
3	rs4946651 *LIN28B* × rs7589318 *POMC* × rs10769908 *STK33* × rs6729809 *LHCGR*	9	1.127	52.57	2	–0.557	7.37	< 0.001

*The results were obtained using the model-based multifactor dimensionality reduction (MB-MDR) method with adjustment for covariates. NH, number of significant high-risk genotypic combinations associated with uterine leiomyoma; *beta* H, coefficient of the logistic regression for significant high-risk genotypic combinations associated with uterine leiomyoma; WH, the Wald test value for significant high-risk genotypic combinations associated with uterine leiomyoma; NL, number of significant low-risk genotypic combinations associated with the uterine leiomyoma; *beta* L, coefficient of the logistic regression for significant low-risk genotypic combinations associated with the uterine leiomyoma; WL, the Wald test value for significant low-risk genotypic combinations associated with uterine leiomyoma; *p*_*perm*_, *p*-value for the permutation test (1,000 permutations); SNP, single-nucleotide polymorphism.*

The following genotype combinations were determined to have the most significant associations with UL: rs11031010 CA *FSHB* × rs2241423 GG *MAP2K5* (*β* = −0.66, *p* = 0.0002), rs222020 TT *GC* × rs4374421 TC *LHCGR* (*β* = -0.44, *p* = 0.0003), rs1782507 AC *FSHB* × rs1884051 AG *ESR1* × rs7766109 AA *F13A1* (*β* = 0.82, *p* = 0.0003), and rs11031010 CC *FSHB* × rs2164808 GA *POMC* × rs7753051 TT *IGF2R* (*β* = 0.49, *p* = 0.0002) (Supplementary Table 9).

**FIGURE 2:**
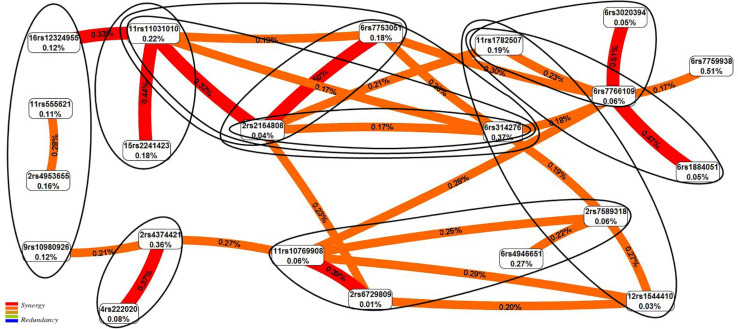
The entropy graph of single-nucleotide polymorphism (SNP)–SNP interactions with uterine leiomyoma based on the multifactor dimensionality reduction (MDR) analysis. Positive values of entropy indicate synergistic interactions, while the negative values indicate redundancy. The polymorphisms are denoted by the chromosome number and rs SNP ID. The red and orange colors denote strong and moderate synergism, respectively. The figures outline the SNP × SNP interactions within the two-, three-, and four-locus models obtained by the MB-MDR method.

The graph of SNP–SNP interactions of 21 polymorphisms (20 SNPs that make up the 11 best models and rs7759938 *LIN28B* independently associated with UL) (Figure 2) suggests that these interactions are concerted; the highest contribution to the entropy is made by interactions rs3020394 *ESR1* × rs7766109 *F13A1* (0.51%), rs2164808 *POMC* × rs7753051 *IGF2R* (0.50%), and polymorphism rs7759938 *LIN28B* (0.51%).

### Gene–Environment Interactions

In total, nine loci were determined to contribute to gene–environment interactions with induced abortions and chronic endometritis, which were significantly associated with UL. The analysis yielded 16 best two-, three-, and four-order models (Table 4 and Supplementary Table 10). Two loci, rs12324955 *FTO* and rs11031010 *FSHB*, were included in the largest number of the best models (11 and 10, respectively) (Table 4). Loci rs4633 *COMT* and rs314280 *LIN28B* were significantly associated with UL through the interaction with induced abortions and chronic endometritis. Among the obtained models of gene–environment interactions, the most optimal for prediction was rs12324955 *FTO* × rs11031010 *FSHB* × chronic endometritis × aborts (OR = 3.90, Test. Bal. Acc. 64.99, sensitivity 62.57, specificity 70.03, Table 4).

**TABLE 4:** The most significant models of gene–environment interactions associated with uterine leiomyoma.

*N*	Models of gene–environment interactions	NH	*beta* H	WH	NL	*beta* L	WL	*p*_*perm*_
Two-order interaction models (*ð* < 1 * 10^–19^)								
1	rs12324955 *FTO* × aborts	2	1.055	09.62	2	–0.999	77.98	<0.001
Three-order interaction models (*ð* < 1 * 10^–21^)								
1	rs11031010 *FSHB* × rs12324955 *FTO* × aborts	3	1.140	104.82	5	–1.018	28.07	<0.001
2	rs2164808 *POMC* × chronic endometritis × aborts	4	1.050	09.18	4	–1.136	102.78	<0.001
3	rs12324955 *FTO* × chronic endometritis × aborts	3	1.111	100.32	2	–1.058	58.60	<0.001
4	rs4374421 *LHCGR* × chronic endometritis × aborts	2	0.944	37.75	4	–1.118	99.14	<0.001
5	rs11031010 *FSHB* × rs2164808 *POMC* × aborts	4	1.094	79.47	4	–0.959	07.31	<0.001
6	rs12324955 *FTO* × rs4374421 *LHCGR* × aborts	4	1.004	97.48	7	–1.107	49.44	<0.001
Four-order interaction models (*ð* < 1 * 10^–24^)								
1	rs11031010 *FSHB* × rs12324955 *FTO* × rs314280 *LIN28B* × aborts	10	1.173	110.02	6	–0.954	95.40	<0.001
2	rs12324955 *FTO* × rs11031010 *FSHB* × chronic endometritis × aborts	4	1.174	108.08	5	–1.067	78.27	<0.001
3	rs11031010 *FSHB* × rs12324955 *FTO* × rs4633 *COMT* × aborts	6	1.173	106.48	6	–1.039	55.41	<0.001
4	rs12324955 *FTO* × rs11031010 *FSHB* × rs7753051 *IGF2R* × aborts	6	1.130	101.08	5	–0.900	55.58	<0.001
5	rs12324955 *FTO* × rs11031010 *FSHB* × rs2164808 *POMC* × aborts	6	1.140	99.01	9	–1.065	08.58	<0.001
6	rs11031010 *FSHB* × rs314276 *LIN28B* × rs2164808 *POMC* × aborts	7	1.124	89.78	7	–1.213	06.78	<0.001
7	rs11031010 *FSHB* × rs2164808 *POMC* × rs7759938 *LIN28B* × aborts	7	1.123	89.60	6	–0.960	15.68	<0.001
8	rs12324955 *FTO* × rs11031010 *FSHB* × rs4374421 *LHCGR* × aborts	6	1.153	89.51	8	–1.014	37.53	<0.001
9	rs12324955 *FTO* × rs4374421 *LHCGR* × chronic endometritis × aborts	5	1.100	09.90	7	–1.138	79.79	<0.001

*The results were obtained using the model-based multifactor dimensionality reduction (MB-MDR) method with adjustment for covariates. NH, number of significant high-risk genotypic, aborts chronic and endometritis combinations associated with uterine leiomyoma; *beta* H, coefficient of the logistic regression for significant high-risk genotypic, aborts and chronic endometritis combinations associated with uterine leiomyoma; WH, the Wald test value for significant high-risk genotypic, aborts and chronic endometritis combinations associated with uterine leiomyoma; NL, number of significant low-risk genotypic, aborts and chronic endometritis combinations associated with the uterine leiomyoma; *beta* L, coefficient of the logistic regression for significant low-risk genotypic, aborts and chronic endometritis combinations associated with the uterine leiomyoma; WL, the Wald test value for significant low-risk genotypic, aborts and chronic endometritis combinations associated with uterine leiomyoma; *p*_*perm*_, *p*-value for the permutation test (1,000 permutations).*

The following combinations of genotypes with chronic endometritis and induced abortions were associated with UL: rs11031010 CC *FSHB* × rs12324955 GG *FTO* × abortion (*β* = 0.92, *p* = 2.38 ^∗^ 10^–12^), rs4374421 TT *LHCGR* × chronic endometritis × abortion (*β* = 0.95, *p* = 2.78 ^∗^ 10^–14^), rs12324955 GG *FTO* × rs11031010 CC *FSHB* × chronic endometritis × abortion (*β* = 0.99, *p* = 5.75 ^∗^ 10^–13^) (Supplementary Table 11).

**FIGURE 3:**
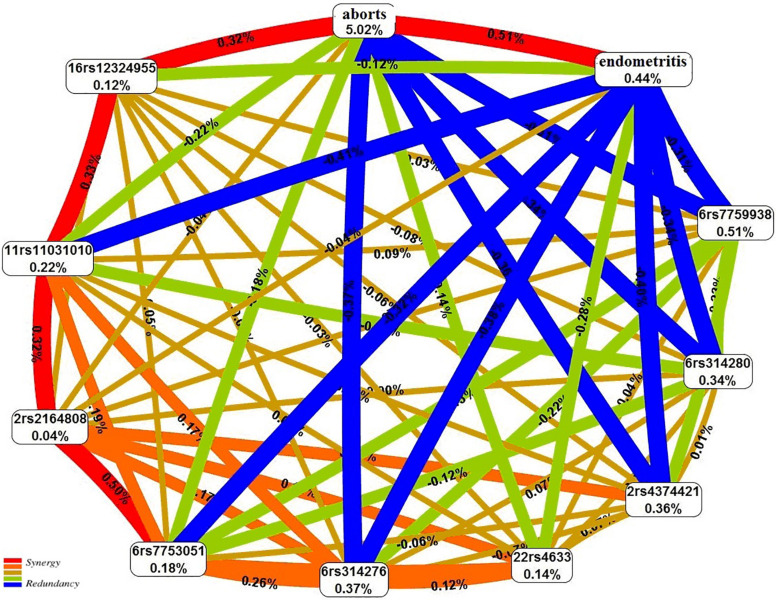
The entropy graph of single-nucleotide polymorphism (SNP) × aborts × chronic endometritis interactions with uterine leiomyoma based on the multifactor dimensionality reduction (MDR) analysis. Positive values of entropy indicate synergistic interactions, while the negative values indicate redundancy. The polymorphisms are denoted by the chromosome number and rs SNP ID. The red and orange colors denote strong and moderate synergism, respectively; brown color denotes the independent effect; and green and blue colors denote moderate and strong antagonism, respectively.

The graph of the interactions between the studied loci, induced abortions, and chronic endometritis suggests that the abortions account for the largest proportion (5.02%) of the trait entropy (Figure 3). In general, the contribution of the most significant gene–environment interactions to UL is 0.31–0.41%, on average.

### Single-Nucleotide Polymorphisms Associated With Uterine Leiomyoma Are Also Associated With Age at Menarche, Height, and Body Mass Index in Adults

The analyses determined 23 loci associated with UL either individually or through gene–gene and gene–environment interactions. We also analyzed whether these SNPs were associated with the other phenotypic characteristics, namely, menarcheal age, height, and BMI in the studied sample (the respective results were reported elsewhere; Ponomarenko et al., 2019). Out of the 23 SNPs, 16 SNPs (69.57%) also manifested association with AAM, height, and/or BMI (Supplementary Table 12). Locus rs4633 *COMT* manifested association with all the above phenotypes, and three loci (rs2164808 *POMC*, rs4374421 *LHCGR*, and rs4946651 *LIN28B*) were also associated with at least two of the three phenotypes analyzed (i.e., age of menarche and/or height and/or BMI).

### Functional Single-Nucleotide Polymorphism

#### Non-synonymous Single-Nucleotide Polymorphisms

None of the 23 polymorphisms associated with UL was missense. However, two of these were in linkage disequilibrium with non-synonymous SNPs. Polymorphism rs4633 was linked to rs4680, which results in an amino acid substitution Val158Met in the COMT protein. This replacement has SIFT Score = 0.02 that corresponds to the predictive value “deleterious.” Another locus, rs2241423 (15q23), was linked to rs7170185, a missense variant (Trp200Arg) in the SKOR1 protein. This amino acid change also has a predictive value of “deleterious” (SIFT Score = 0).

#### Regulatory Effects

The data on regulatory effects of the UL-associated SNPs are given in Supplementary Table 13. Locus rs4633 *COMT* appeared to have the most pronounced effect; the significant regulatory potential was also determined for rs314280 *LIN28B*, rs2164808 *POMC*, rs12324955 *FTO*, and rs10769908 *STK33* (Supplementary Table 13).

The 514 SNPs linked to the UL-associated loci were analyzed for their regulatory potential (Supplementary Table 14). Among these, more than 460 had regulatory significance. The most pronounced regulatory potential was determined for loci linked to polymorphisms rs314276 and rs7759938 of the *LIN28B* gene (two SNPs), rs11031010, rs555621, and rs1782507 of the *FSHB* gene (four SNPs), rs2241423 *MAP2K* (>10 SNPs), rs10769908 *STK33* (two SNPs), and rs4633 *COMT* (three SNPs) (Supplementary Table 14).

Importantly, the loci that are linked to the UL-associated SNPs showed their regulatory effects in organs and tissues related to pathogenesis of UL, i.e., ovaries, muscle tissue, adipose tissue, various brain regions (hypothalamus, pituitary, etc.), and liver (Commandeur et al., 2015).

#### Expression Quantitative Trait Loci

Eight of the 23 UL-associated SNPs manifested their association (*p* < 5 ^∗^ 10^–5^, *p*_*FDR*_ < 0.05) with the expression level of seven genes [*COMT*, *DNAJC27*, *F13A1*, *KIAA0368*, *SLC22A1*, *C11orf46* (*ARL14EP*), and *MAP2K5*] in peripheral blood (*cis*-eQTL) (Supplementary Table 15). Five of these SNPs are also linked to the other *cis-*eQTL loci affecting mRNA transcription level in blood (*MAP2K5*, *SLC22A1*, *DNAJC27*, *KIAA0368*, and *COMT*) (Supplementary Table 16). No *trans*-eQTL SNPs were determined (FDR > 0.05).

According to the data of the Genotype-Tissue Expression (GTEx) project, 16 SNPs of the 23 associated with UL had *cis*-eQTL significance in various tissues and organs, including those that are related to pathogenesis of UL (*p* < 8 ^∗^ 10^–5^, *p*_*FDR*_ ≤ 0.05) (Supplementary Table 17). Allele T (ref) of rs7759938 *LIN28B* is associated with the lower expression of the gene in hypophysis (β = -0.50, *ð* = 1.3 ^∗^ 10^–11^, *p*_*FDR*_ ≤ 0.05), while alternative allele C has a protective effect for UL (OR = 0.74).

Sixteen UL-associated loci manifested strong linkage to more than 380 polymorphisms affecting gene expression (*p* < 8.5 ^∗^ 10^–5^, *p*_*FDR*_ ≤ 0.05) in various organs and tissues (Supplementary Table 18). Loci rs1782507, rs11031010, and rs555621 of *FSHB* appeared to have the most pronounced effect as they were linked to more than 120 SNPs affecting expression of the *FSHB*, *ARL14EP*, and *RP4-710M3.1* genes in more than 25 organs and tissues.

Overall, 18 of the 23 UL-associated loci have *cis*-eQTL value: they affect expression of 28 genes. Among these, two SNPs are associated with the mRNA transcription level independently, and 16 SNPs are both individually associated with and linked to other loci affecting gene expression levels.

### Pathway Analyses

The roles in biological processes or molecular functions of the UL-associated genes (Supplementary Table 13) and those whose expression is affected by the UL-associated 23 loci according to the eQTL analysis (Supplementary Tables 15–18) were analyzed *in silico* using annotations from the Gene Ontology databases. We found evidence of enrichment for pathways involved in the follicle-stimulating hormone signaling pathway (FDR = 0.002), hormone ligand-binding receptors (FDR = 0.002), ovarian follicle development (FDR = 0.003), and other pathways related to development of the female reproductive organs and hormone-mediated signaling pathways (FDR ≤ 0.05) (Supplementary Table 19).

**FIGURE 4:**
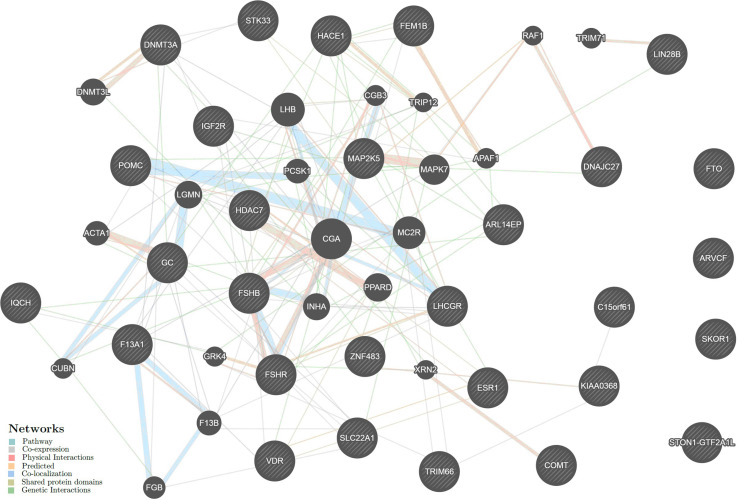
The interaction networks of the candidate genes for uterine leiomyoma inferred using GeneMANIA (http://genemania.org). The candidate genes determined in the present study are cross-shaded.

The intergenic interactions of the above genes were analyzed using GeneMANIA (see footnote 20). The data on six genes, *LINC00577*, *RP11-509E16.1*, *RP11-288H12.3*, *RP4-710M3.1*, *RP11-34F13.2*, and *IQCH-AS1*, were absent in the database. Therefore, the resulting network included 29 UL-associated genes and 20 other genes significantly interacting with them (Figure 4). The intergenic interactions are executed through pathway (26.52%), co-expression (25.00%), physical (22.76%) and predicted (11.09%) interactions, co-localization (8.47%), shared protein domains (5.87%), and genetic interactions (0.29%) (Supplementary Table 20).

## Discussion

This study reports for the first time the associations of 23 candidate loci for AAM with UL. Locus rs7759938 of the *LIN28B* gene demonstrated the most significant associations: its allele Ñ decreased a risk for UL according to the dominant model (OR = 0.74). This locus is also associated with UL within most significant four-order model of polymorphisms interactions with induced abortions.

According to the GTEx Portal database, rs7759938 and 27 SNPs linked to it have the *cis-*eQTL significance and may affect expression of *LIN28B* in the pituitary gland (allele Ñ of rs7759938 increases expression of *LIN28B*). Association of this SNP with menarcheal age was reported previously (Perry et al., 2009; Elks et al., 2010; Delahanty et al., 2013; Perry et al., 2014; Ponomarenko et al., 2019). Several studies suggested that rs7759938 might play a role in the pubertal development and adult height (Elks et al., 2010; Lango Allen et al., 2010; Widén et al., 2010; Leinonen et al., 2012; Cousminer et al., 2013; Perry et al., 2014). Two polymorphisms, rs9391253 and rs314263, which are linked to rs7759938 (*r*^2^ ≥ 0.94), were associated with height (Berndt et al., 2013; Wood et al., 2014). Importantly, allele C of rs7759938 was previously determined as associated with later menarche, delayed pubertal growth, and high height (Perry et al., 2009, 2014, Elks et al., 2010; Delahanty et al., 2013). On the other hand, the present study suggested this allele as a protective factor for UL. That means that AAM and a risk for UL may have a shared genetic basis.

Two SNPs of the *LIN28B* gene (rs314276 and rs4946651) were involved in five out of 11 most significant models of epistatic interactions, and polymorphism rs314280 *LIN28B* was associated with UL only through interaction with induced abortions and chronic endometritis. All these polymorphisms were reported as associated with AAM (He et al., 2009; Ong et al., 2009; Sulem et al., 2009; Carty et al., 2013). There is evidence that rs314276 may play a role in the pubertal development and height (Ong et al., 2009; Cousminer et al., 2014), BMI, and weight in females (Ong et al., 2011). Previously, we reported the association of the rs314280 and rs4946651 polymorphisms with BMI of adults (Ponomarenko et al., 2019). Two polymorphisms, rs9391253 and rs314263, which are linked to rs314276 (*r*^2^ ≥ 0.94), were associated with height (Berndt et al., 2013; Wood et al., 2014). The data of GTEx Portal suggest that the UL-associated SNPs of *LIN28B* have *cis*-eQTL significance: rs314276 (with 20 SNPs linked to it) and rs4946651 (with 15 SNPs linked to it) are associated with expression level of *LINC00577* in the cortex and basal ganglia of the brain, *LIN28B* in the pituitary gland, and *HACE1* in skeletal muscle; rs314276 and 27 are linked to SNPs that may affect expression of the *LIN28B* gene in the pituitary gland.

The above four UL-associated SNPs of the *LIN28B* gene are located in region 6q16.3-q21 about 38 kb from each other and are linked to about 40 SNPs with the regulatory potential and *cis*-eQTL values (affect transcription of the *LIN28B*, *LINC00577*, and *HACE1* genes).

LIN28B (lin-28 homolog B) is a member of the LIN28 family of proteins and functions as a repressor of the *let-7* family of miRNA (Tsialikas and Romer-Seibert, 2015). These miRNAs contribute to developmental processes in invertebrate animals, while their role in vertebrates is not well understood. Some data suggest that the let-7 miRNAs may contribute to cell cycle control and oncogenesis (Tsialikas and Romer-Seibert, 2015). The target genes of the *let-7* miRNA, *Kras*, *Myc*, *Hmga2*, and *Igf2bp1* are involved in body size control and metabolism in mammals (Zhu et al., 2011). The function of *LINC00577* (*LIN28B antisense RNA 1*) is unknown; according to the information from the Ensembl database, it belongs to the genes encoding long intergenic non-coding RNAs (lincRNAs), which interact with proteins, DNA, and other RNAs, and thus perform important regulatory functions (Ulitsky and Bartel, 2013). A protein product of the *HACE1* (*HECT domain and ankyrin repeat containing E3 ubiquitin protein ligase 1*) gene is a potential tumor suppressor and participates in the specific tagging of target proteins that subsequently results in proteasome degradation^21^.

The rs555621, rs11031010, and rs1782507 polymorphisms of the *FSHB* gene appeared in the largest number of the most significant models of intergenic interactions (7 out of 11 models); rs11031010 *FSHB* was presented in 10 out of 16 most significant models gene–environment interactions associated with UL. Recently, we determined association of rs555621 with BMI of adults (Ponomarenko et al., 2019). The above three loci were also associated with AAM (He et al., 2010), and rs11031010 was suggested to be a risk factor of PCOS and affect a level of luteinizing hormone (LH) in patients (Tian et al., 2016).

The *FSHB* gene encodes a beta-subunit of the follicle-stimulating hormone. This hormone has multiple functions in the female reproductive system, including stimulation proliferation of follicular granulosa cells, rescue of follicles from apoptosis, and synthesis of receptors for the LH on these cells before ovulation.

The recent meta-analysis of 11 GWAS determined the region on chromosome 11 (11p14.1, rs74485684) harboring the *FSHB* gene as that associated with endometriosis (Sapkota et al., 2017). Importantly, rs11031010 *FSHB* analyzed in the present study is located just 1.9 kb from the above locus. On the other hand, the first GWAS of UL in European populations (Rafnar et al., 2018) replicated three of the 19 previously reported endometriosis variants (Sapkota et al., 2017). Several loci associated with endometriosis, including rs11031006 located in 11p14.1, were reported for their association with UL in the recent GWAS (Gallagher et al., 2019). Importantly, the rs11031010 polymorphism of the *FSHB* gene analyzed in the present study is linked to rs11031006 (*r*^2^ = 0.64).

An epidemiological meta-analysis of 402,868 women suggested at least twofold higher risk for UL in patients with a history of endometriosis (Gallagher et al., 2019). It was hypothesized that both UL and endometriosis were underlain by hormone-related factors (Rafnar et al., 2018) and thus might have a shared genetic basis (Gallagher et al., 2019). This assumption was supported by the recent findings of syntropic genes for these diseases (Rafnar et al., 2018; Gallagher et al., 2019).

There is evidence that some loci linked to rs11031010 may contribute to various reproductive characters, such as concentration of the luteinizing (rs11031002, *r*^2^ = 0.76) and follicle-stimulating (rs11031005, *r*^2^ = 0.64) hormones in blood plasma (Ruth et al., 2016), and age at menopause (rs12294104, *r*^2^ = 0.61) (Stolk et al., 2012). The SNPs upstream of the *FSHB* transcription start were implicated into a key role in many reproductive processes (Gajbhiye et al., 2018). It is generally acknowledged that women with late menopause or/and early menarche have, on average, an increased lifetime exposure to estrogen due to the larger number of ovulatory cycles. Since mitotic activity of myometrium is the highest during the luteal phase of the cycle, a longer history of menses may theoretically increase a risk for UL. Likewise, this risk may be higher due to the increasing LH level (Wise and Laughlin-Tommaso, 2016).

The present study determined that the rs2164808 polymorphism of the *POMC* gene was involved in the largest number of the most significant models of gene–gene (four out of 11 models) and gene–environment (five out of 16 models) interactions associated with UL. Previously, we reported the association of the rs2164808 locus with BMI of adults (Ponomarenko et al., 2019). The association of this locus with AAM was first reported by He et al. (2010). Polymorphism rs1561288, which is linked to rs2164808 (*r*^2^ = 0.25), was associated with BMI (Graff et al., 2013). Locus rs2164808 and linked to it rs4665765 are both associated with the transcription level of *DNAJC27* (according to the Blood eQTL browser) and *RP11-509E16.1* (according to the GTEx Portal database). The data from the SNP Function Prediction and rSNPBase suggest a significant regulatory effect of rs2164808 (proximal regulation and RNA binding protein mediated regulation effects, regulatory potential score 0.334). The *POMC* (*pro-opiomelanocortin-alpha*) gene encodes a preproprotein that undergoes tissue-specific posttranslational modifications resulting in up to 10 biologically active peptides, which participate in various cellular processes, including those related to UL (e.g., lipolysis, mobilization of fatty acids, steroidogenesis, and adrenal development) (see footnote 21). The product of the *DNAJC27* [*DnaJ heat shock protein family* (*Hsp40*) *member C27*] gene is a GTPase that is associated with the MEK/ERK pathway and may induce cell transformation (see footnote 21). The function of *RP11-509E16.1* is unknown; according to the information from the Ensembl database^22^, it belongs to the genes encoding lincRNAs, which interact with proteins, DNA, and other RNAs and thus perform important regulatory functions (Ulitsky and Bartel, 2013). The polymorphisms located in the regions of the *POMC*, *DNAJC27* (*RBJ*), and *ADCY3* genes were associated with pubertal development, height, BMI, obesity, and type I diabetes mellitus (Gudbjartsson et al., 2008; Lango Allen et al., 2010; Speliotes et al., 2010; Bradfield et al., 2011; Berndt et al., 2013; Cousminer et al., 2013; He et al., 2015).

One more finding of the present study is that associations with UL were determined not only for candidate SNPs for AAM (Supplementary Table 4) but also for seven polymorphisms manifesting association or tagged with the traits related to menarche (e.g., sex steroid hormone and vitamin D metabolism, and physical characteristics; Supplementary Tables 4, 12). These menarche-related traits are important for pathogenesis of UL (Commandeur et al., 2015; Wise and Laughlin-Tommaso, 2016). The latter are involved in the sex steroid hormone pathway (rs3020394 and rs1884051 *ESR1*, rs4633 *COMT*, and rs12324955 *FTO* and rs7766109 *F13A1*) and vitamin D metabolism (rs1544410 *VDR* and rs222020 *GC*) (Supplementary Table 4, see footnote 21), which are important for UL development (Commandeur et al., 2015; Wise and Laughlin-Tommaso, 2016). For example, rs4633 *COMT*, which is also associated with AAM, height, and BMI in the studied sample (Ponomarenko et al., 2019) and linked to it rs4680 is associated with the *COMT* expression level in peripheral blood (according to the Blood eQTL browser). The activity of the COMT enzyme is elevated in human leiomyoma tissue as compared with normal myometrium; since this enzyme is essential for estrogen metabolism, the observed association suggests a possible causal role of COMT in UL formation (Commandeur et al., 2015). In addition, the rs4680 polymorphism encodes a replacement substitution Val158Met in COMT: the Met variant has 40% lower activity as compared with the Val one (Chen et al., 2004). Further support of the possible contribution of rs4680 to UL comes from the meta-analysis by Feng et al. (2013), who reported the association of rs4680 with UL.

We did not find a direct association of AAM with UL but determined that 23 candidate loci for AAM might be associated with UL. Among these, several were also associated with age of menarche, height, and/or BMI of adults in the study sample. This may suggest that menarcheal age *per se* is not a primary risk factor for UL in the studied sample (the population of Russia) and that other factors may be of greater importance. Specifically, we found that the history of induced abortions significantly increased the risk of UL in the study sample (OR_*adj*_ = 2.78). Our results support the recent findings by other researchers (Shen et al., 2017; Song et al., 2017). Induced abortions are common birth control method in Russia (and generally in the former USSR) (Sedgh et al., 2007; David et al., 2007; Douglas et al., 2014), and Russia (along with Cuba) is a leader by this indicator in the world (Sedgh et al., 2007). Induced abortions in the first trimester of pregnancy may cause multiple health complications, including PCOS, hyperprolactinemia, anovulatory menstruation, disorders of the luteal phase of the menstrual cycle, and post-abortion endometritis (Douglas et al., 2014). Induced recurring abortions result in injuries of the uterus. The patient cohort in the present study included 44.99% individuals who had at least two induced abortions. Uterine injuries may induce the expression of the growth factor that speed ups cellular proliferation, suppresses apoptosis, and increases extracellular matrix production (Wise and Laughlin-Tommaso, 2016). Indeed, our results suggest that the risk for UL directly correlates with the number of the abortions in the anamnesis (OR_*adj*_ = 1.69): it increases from OR = 3.85 (95% CI 2.79–5.32, *p* < 0.001) in a case of two abortions up to OR = 13.41 (95% CI 7.22–25.24, *p* < 0.001) with four and more abortions. Similar results were reported by Song et al. (2017) who showed that the higher number of induced abortions was associated with an increased risk of UL (one induced abortion, OR = 1.32; two induced abortions, OR = 1.45; and ≥ 3 induced abortions, OR = 1.62). PCOS was associated with a 65% increased risk of UL (Wise and Laughlin-Tommaso, 2016). The mechanisms by which PCOS may elevate risk for UL include increased levels of LH or unopposed estrogens and its association with hyperinsulinemia (Wise and Laughlin-Tommaso, 2016). Induced abortions may also be associated with other hormone-related diseases (e.g., breast cancer) (Schneider et al., 2014). Inflammation in the uterus (stress from chronic inflammation) seems a quite possible etiologic factor of UL: studies showed that inflammation increases the production of extracellular matrix and decreases apoptosis in UL (Commandeur et al., 2015; Wise and Laughlin-Tommaso, 2016).

## Conclusion

Candidate genes for AAM are associated with UL. Locus rs7759938 *LIN28B* is associated with UL independently; 20 loci are associated within the 11 most significant intergenic interaction models, and nine loci may contribute to the risk of the disorder through the 16 most significant gene–environment interactions. These associations probably result from multiple effects of more than 510 polymorphisms linked to the above SNPs and implicated in various metabolic processes in organs and tissues that are related to pathogenesis of UL.

## Data Availability Statement

The datasets generated for this study can be found in EVA at the following accessions Project: PRJEB40782, Analyses: ERZ1645102.

## Ethics Statement

The studies involving human participants were reviewed and approved by Regional Ethics Committee of Belgorod State University. The patients/participants provided their written informed consent to participate in this study.

## Author Contributions

IP, MC, and VD made substantial contributions to conception and design, drafted the manuscript, and gave the final approval of the version to be published. ER, AP, IV, IS, and AY analyzed and interpreted of data, drafted the manuscript, and gave the final approval of the version to be published. All the authors are accountable for all aspects of the work in ensuring that questions related to the accuracy or integrity of any part of the work are appropriately investigated and resolved.

## Conflict of Interest

The authors declare that the research was conducted in the absence of any commercial or financial relationships that could be construed as a potential conflict of interest.

## Footnotes

1 https://pubs.broadinstitute.org/mammals/haploreg/haploreg.php

2 https://www.regulomedb.org/regulome-search/

3 http://rsnp3.psych.ac.cn/

4 https://snpinfo.niehs.nih.gov/snpinfo/snpfunc.html

5 http://genenetwork.nl/bloodeqtlbrowser/

6 http://www.gtexportal.org/

7 http://zzz.bwh.harvard.edu/plink/

8 http://www.ssg.uab.edu/gmdr

9 http://sourceforge.net/projects/gmdr

10 http://www.multifactordimensionalityreduction.org/

11 http://sourceforge.net/projects/mdr

12 https://pubs.broadinstitute.org/mammals/haploreg/haploreg.php

13 http://sift.jcvi.org/

14 https://snpinfo.niehs.nih.gov/snpinfo/snpfunc.html

15 http://rsnp3.psych.ac.cn/

16 https://www.regulomedb.org/regulome-search/

17 http://genenetwork.nl/bloodeqtlbrowser/

18 http://www.gtexportal.org/

19 http://geneontology.org

20 http://genemania.org

21 http://www.genecards.org/

22 http://www.ensembl.org/
