# Supplementary material for: Candidate Genes for Age at Menarche Are Associated With Uterine Leiomyoma
Source: Front Genet. 2021 Jan 22;11:512940. doi: 10.3389/fgene.2020.512940 (PMC7863975; doi:10.3389/fgene.2020.512940)
Supplement: Supplementary file 1 [file Data_Sheet_1.zip › SupMaterial 16-12-2020/Sup_Table_12.docx]

Supplementary Table 12 Association of UL-associated SNPs with age at menarche, height and BMI in the studied sample*

| Chr | SNP | gene | age at menarche* | height* | BMI* | uterine leiomyoma |
| --- | --- | --- | --- | --- | --- | --- |
| 1 | rs1514175 | *TNNI3K* |  |  |  |  |
| 1 | rs466639 | *RXRG* |  |  |  |  |
| 1 | rs7538038 | *KISS1* |  | **yes** |  |  |
| 2 | rs713586 | *RBJ* |  | **yes** |  |  |
| 2 | rs2164808 | *POMC* | **yes** |  | **yes** | **yes** |
| 2 | rs7589318 | *POMC* |  |  |  | **yes** |
| 2 | rs4374421 | *LHCGR* | **yes** |  | **yes** | **yes** |
| 2 | rs7579411 | *LHCGR* | **yes** | **yes** |  |  |
| 2 | rs6729809 | *LHCGR* |  |  | **yes** | **yes** |
| 2 | rs4953616 | *LHCGR* |  |  | **yes** |  |
| 2 | rs6732220 | *FSHR* |  | **yes** |  |  |
| 2 | rs4953655 | *FSHR* |  |  | **yes** | **yes** |
| 2 | rs887912 | *FANCL* |  |  |  |  |
| 2 | rs12617311 | *PLCL1* | **yes** | **yes** | **yes** |  |
| 3 | rs6438424 | *3q13.32* | **yes** |  |  |  |
| 4 | rs2013573 | *UGT2B4* |  |  |  |  |
| 4 | rs13111134 | *UGT2B4* |  |  |  |  |
| 4 | rs222003 | *GC* |  |  |  |  |
| 4 | rs222020 | *GC* |  |  |  | **yes** |
| 4 | rs3756261 | *EGF* |  | **yes** |  |  |
| 5 | rs757647 | *KDM3B* |  |  |  |  |
| 6 | rs7766109 | *F13A1* |  | **yes** |  | **yes** |
| 6 | rs4946651 | *LIN28B* | **yes** |  | **yes** | **yes** |
| 6 | rs7759938 | *LIN28B* | **yes** |  |  | **yes** |
| 6 | rs314280 | *LIN28B* |  |  | **yes** | **yes** |
| 6 | rs314276 | *LIN28B* | **yes** |  |  | **yes** |
| 6 | rs3020394 | *ESR1* |  |  | **yes** | **yes** |
| 6 | rs1884051 | *ESR1* |  |  |  | **yes** |
| 6 | rs7753051 | *IGF2R* |  | **yes** |  | **yes** |
| 7 | rs1079866 | *INHBA* |  |  |  |  |
| 8 | rs2288696 | *FGFR1* |  |  |  |  |
| 9 | rs2090409 | *TMEM38B* | **yes** | **yes** |  |  |
| 9 | rs10980926 | *ZNF483* |  | **yes** |  | **yes** |
| 9 | rs10441737 | *ZNF483* |  | **yes** |  |  |
| 11 | rs10769908 | *STK33* | **yes** |  |  | **yes** |
| 11 | rs555621 | *FSHB* |  |  | **yes** | **yes** |
| 11 | rs11031010 | *FSHB* |  |  |  | **yes** |
| 11 | rs1782507 | *FSHB* |  |  |  | **yes** |
| 11 | rs6589964 | *BSX* | **yes** | **yes** |  |  |
| 12 | rs1544410 | *VDR* |  |  | **yes** | **yes** |
| 14 | rs999460 | *NKX2-1* |  |  | **yes** |  |
| 14 | rs4986938 | *ESR2* |  | **yes** |  |  |
| 15 | rs2241423 | *MAP2K5* |  |  |  | **yes** |
| 16 | rs12444979 | *GPRC5B* |  |  |  |  |
| 16 | rs9939609 | *FTO* |  | **yes** |  |  |
| 16 | rs12324955 | *FTO* |  |  |  | **yes** |
| 18 | rs1398217 | *SKOR2* |  |  | **yes** |  |
| 19 | rs2252673 | *INSR* | **yes** |  |  |  |
| 20 | rs1073768 | *GHRH* | **yes** | **yes** | **yes** |  |
| 22 | rs4633 | *COMT* | **yes** | **yes** | **yes** | **yes** |
| X | rs5930973 | *CD40LG* |  |  |  |  |
| X | rs3092921 | *CD40LG* |  |  |  |  |

**yes**, associated.

highlighted in yellow SNPs are not directly associated with AAM, but manifested association or tagged with the traits related to menarche (e.g., polycystic ovary syndrome, vitamin D metabolism, physical characteristics, etc., Supplementary table 4).

* Ponomarenko et al.,2019.
