# Supplementary material for: Candidate Genes for Age at Menarche Are Associated With Uterine Leiomyoma
Source: Front Genet. 2021 Jan 22;11:512940. doi: 10.3389/fgene.2020.512940 (PMC7863975; doi:10.3389/fgene.2020.512940)
Supplement: Supplementary file 1 [file Data_Sheet_1.zip › SupMaterial 16-12-2020/Sup_Table_3.docx]

Supplementary Table 3 Expression QTLs of the studied SNPsa.

| Chr | SNP | Gene/Region | Database | | | | |
| --- | --- | --- | --- | --- | --- | --- | --- |
|  |  |  | rSNPBase | HaploReg | RegulomeDB | GTExportal | Blood eQTL browser |
| 1 | rs1514175 | *TNNI3K* | yes |  |  |  |  |
| 1 | rs466639 | *RXRG* | yes |  |  |  |  |
| 1 | rs7538038 | *KISS1* | yes |  |  |  |  |
| 2 | rs713586 | *RBJ* | yes | 34 hits | 1f | 16 hit | yes |
| 2 | rs2164808 | *POMC* | yes | 1 hit |  |  | yes |
| 2 | rs7589318 | *POMC* | yes | 1 hit |  |  | yes |
| 2 | rs4374421 | *LHCGR* |  |  |  |  |  |
| 2 | rs7579411 | *LHCGR* | yes | 1 hit |  | 1 hit |  |
| 2 | rs6729809 | *LHCGR* |  |  |  | 1 hit |  |
| 2 | rs4953616 | *LHCGR* |  |  |  | 2 hit |  |
| 2 | rs6732220 | *FSHR* | yes | 1 hit |  | 1 hit |  |
| 2 | rs4953655 | *FSHR* | yes | 1 hit |  | 1 hit |  |
| 2 | rs887912 | *FANCL* |  |  |  |  |  |
| 2 | rs12617311 | *PLCL1* |  |  |  |  |  |
| 3 | rs6438424 | *3q13.32* | yes | 1 hit |  | 3 hit |  |
| 4 | rs2013573 | *UGT2B4* | yes |  |  | 1 hit |  |
| 4 | rs13111134 | *UGT2B4* |  |  |  | 1 hit |  |
| 4 | rs222003 | *GC* |  |  |  |  |  |
| 4 | rs222020 | *GC* | yes |  |  |  |  |
| 4 | rs3756261 | *EGF* | yes |  |  |  |  |
| 5 | rs757647 | *KDM3B* | yes |  | 1f |  |  |
| 6 | rs7766109 | *F13A1* | yes | 1 hit |  |  | yes |
| 6 | rs4946651 | *LIN28B* |  | 1 hit |  | 1 hit |  |
| 6 | rs7759938 | *LIN28B* | yes |  |  | 1 hit |  |
| 6 | rs314280 | *LIN28B* | yes | 1 hit |  | 1 hit |  |
| 6 | rs314276 | *LIN28B* | yes |  |  | 1 hit |  |
| 6 | rs3020394 | *ESR1* | yes |  |  |  |  |
| 6 | rs1884051 | *ESR1* | yes |  |  |  |  |
| 6 | rs7753051 | *IGF2R* | yes | 9 hits |  | 10 hit | yes |
| 7 | rs1079866 | *INHBA* |  |  |  |  |  |
| 8 | rs2288696 | *FGFR1* | yes |  |  |  |  |
| 9 | rs2090409 | *TMEM38B* |  |  |  |  |  |
| 9 | rs10980926 | *ZNF483* | yes | 2 hits |  | 1 hit | yes |
| 9 | rs10441737 | *ZNF483* |  | 2 hits |  | 1 hit | yes |
| 11 | rs10769908 | *STK33* | yes | 23 hits |  | 8 hit |  |
| 11 | rs555621 | *FSHB* | yes | 7 hits |  | 1 hit |  |
| 11 | rs11031010 | *FSHB* |  | 8 hits |  | 5 hit | yes |
| 11 | rs1782507 | *FSHB* | yes | 12 hits |  | 14 hit |  |
| 11 | rs6589964 | *BSX* | yes | 1 hit |  |  |  |
| 12 | rs1544410 | *VDR* | yes | 2 hits |  |  |  |
| 14 | rs999460 | *NKX2-1* |  |  |  |  |  |
| 14 | rs4986938 | *ESR2* | yes | 11 hits |  | 2 hit |  |
| 15 | rs2241423 | *MAP2K5* | yes | 16 hits |  | 21 hit | yes |
| 16 | rs12444979 | *GPRC5B* |  | 70 hits |  | 32 hit |  |
| 16 | rs9939609 | *FTO* | yes |  |  |  |  |
| 16 | rs12324955 | *FTO* | yes |  |  |  |  |
| 18 | rs1398217 | *SKOR2* | | 2 hits |  | 2 hit | yes |
| 19 | rs2252673 | *INSR* |  |  |  | 1 hit |  |
| 20 | rs1073768 | *GHRH* | yes | 2 hits |  | 4 hit | yes |
| 22 | rs4633 | *COMT* | yes | 5 hits |  | 3 hit | yes |
| X | rs5930973 | *CD40LG* |  |  |  |  |  |
| X | rs3092921 | *CD40LG* |  |  |  |  |  |

The effect on gene expression was estimated using the online tools HaploReg (v4.1) (http://archive.broadinstitute.org/mammals/haploreg/haploreg.php), RegulomeDB (Version 1.1) (http://regulome.stanford.edu/), rSNPBase (http://rsnp.psych.ac.cn/index.do), Blood eQTL browser (http://genenetwork.nl/bloodeqtlbrowser/), and GTExportal (http://www.gtexportal.org/).
