# Supplementary material for: Candidate Genes for Age at Menarche Are Associated With Uterine Leiomyoma
Source: Front Genet. 2021 Jan 22;11:512940. doi: 10.3389/fgene.2020.512940 (PMC7863975; doi:10.3389/fgene.2020.512940)
Supplement: Supplementary file 1 [file Data_Sheet_1.zip › SupMaterial 16-12-2020/Sup_Table_5.docx]

Supplementary table 5 The data on the allele and genotype frequencies of the 52 SNPs in the uterine leiomyoma group

| Chr | SNP | Minor allele | Major allele | Minor allele frequency | Number of the studied chromosomes | | Genotype distribution* | Ho | He | Р HWE |
| --- | --- | --- | --- | --- | --- | --- | --- | --- | --- | --- |
| 1 | rs1514175 | T | C | 0.377 | 1128 | | 91/243/230 | 0.431 | 0.470 | 0.049 |
| 1 | rs466639 | T | C | 0.112 | 1132 | | 4/119/443 | 0.210 | 0.199 | 0.287 |
| 1 | rs7538038 | G | A | 0.236 | 1134 | | 31/206/330 | 0.363 | 0.361 | 1.000 |
| 2 | rs713586 | C | T | 0.418 | 1130 | | 98/276/191 | 0.489 | 0.487 | 1.000 |
| 2 | rs2164808 | A | G | 0.483 | 1134 | | 130/288/149 | 0.508 | 0.499 | 0.737 |
| 2 | rs7589318 | A | G | 0.309 | 1128 | | 57/234/273 | 0.415 | 0.427 | 0.554 |
| 2 | rs4374421 | C | T | 0.292 | 1056 | | 53/202/273 | 0.383 | 0.413 | 0.092 |
| 2 | rs7579411 | T | C | 0.417 | 1114 | | 103/259/195 | 0.465 | 0.486 | 0.297 |
| 2 | rs6729809 | C | T | 0.317 | 1090 | | 47/251/247 | 0.461 | 0.433 | 0.165 |
| 2 | rs4953616 | C | T | 0.280 | 1120 | | 38/238/284 | 0.425 | 0.404 | 0.249 |
| 2 | rs6732220 | G | C | 0.243 | 1130 | | 27/220/318 | 0.389 | 0.367 | 0.171 |
| 2 | rs4953655 | G | A | 0.228 | 1134 | | 24/211/332 | 0.372 | 0.353 | 0.233 |
| 2 | rs887912 | A | G | 0.224 | 1086 | | 28/187/328 | 0.344 | 0.347 | 0.806 |
| 2 | rs12617311 | A | G | 0.310 | 1128 | | 64/222/278 | 0.394 | 0.428 | 0.061 |
| 3 | rs6438424 | C | A | 0.467 | 1118 | | 121/280/158 | 0.501 | 0.498 | 0.932 |
| 4 | rs2013573 | A | G | 0.182 | 1132 | | 14/178/374 | 0.315 | 0.298 | 0.206 |
| 4 | rs13111134 | A | G | 0.228 | 1134 | | 29/200/338 | 0.353 | 0.352 | 1.000 |
| 4 | rs222003 | C | G | 0.073 | 1134 | | 2/79/486 | 0.139 | 0.136 | 0.759 |
| 4 | rs222020 | C | T | 0.129 | 1136 | | 9/128/431 | 0.225 | 0.224 | 1.000 |
| 4 | rs3756261 | G | A | 0.085 | 1124 | | 4/88/470 | 0.157 | 0.156 | 1.000 |
| 5 | rs757647 | T | C | 0.223 | 1114 | | 25/198/334 | 0.356 | 0.346 | 0.624 |
| 6 | rs7766109 | G | A | 0.479 | 1128 | | 130/280/154 | 0.497 | 0.499 | 0.933 |
| 6 | rs4946651 | A | G | 0.392 | 1134 | | 97/251/219 | 0.443 | 0.477 | 0.094 |
| 6 | rs7759938 | C | T | 0.259 | 1132 | | 45/203/318 | 0.359 | 0.384 | 0.125 |
| 6 | rs314280 | T | C | 0.382 | 1122 | | 87/255/219 | 0.455 | 0.472 | 0.372 |
| 6 | rs314276 | A | C | 0.289 | 1090 | | 50/215/280 | 0.395 | 0.411 | 0.349 |
| 6 | rs3020394 | G | A | 0.304 | | 1130 | 51/241/273 | 0.427 | 0.423 | 0.921 |
| 6 | rs1884051 | G | A | 0.295 | | 1134 | 48/238/281 | 0.420 | 0.416 | 0.920 |
| 6 | rs7753051 | C | T | 0.292 | | 1134 | 50/231/286 | 0.407 | 0.413 | 0.761 |
| 7 | rs1079866 | C | G | 0.184 | | 1134 | 16/177/374 | 0.312 | 0.301 | 0.405 |
| 8 | rs2288696 | T | C | 0.188 | | 1132 | 18/177/371 | 0.313 | 0.306 | 0.680 |
| 9 | rs2090409 | T | G | 0.370 | | 1074 | 69/259/209 | 0.482 | 0.466 | 0.459 |
| 9 | rs10980926 | A | G | 0.301 | | 1122 | 55/228/278 | 0.406 | 0.421 | 0.423 |
| 9 | rs10441737 | C | T | 0.317 | | 1076 | 55/231/252 | 0.429 | 0.433 | 0.843 |
| 11 | rs10769908 | C | T | 0.472 | | 1114 | 129/268/160 | 0.481 | 0.499 | 0.444 |
| 11 | rs555621 | G | A | 0.383 | | 1134 | 73/288/206 | 0.508 | 0.473 | 0.091 |
| 11 | rs11031010 | A | C | 0.107 | | 1110 | 9/101/445 | 0.182 | 0.191 | 0.263 |
| 11 | rs1782507 | C | A | 0.381 | | 1122 | 80/267/214 | 0.476 | 0.472 | 0.858 |
| 11 | rs6589964 | A | C | 0.493 | | 1130 | 140/277/148 | 0.490 | 0.500 | 0.674 |
| 12 | rs1544410 | A | G | 0.375 | | 1122 | 86/249/226 | 0.444 | 0.469 | 0.208 |
| 14 | rs999460 | A | G | 0.329 | | 1134 | 65/243/259 | 0.429 | 0.442 | 0.506 |
| 14 | rs4986938 | A | G | 0.363 | | 1134 | 76/260/231 | 0.459 | 0.463 | 0.856 |
| 15 | rs2241423 | A | G | 0.179 | | 1126 | 12/177/374 | 0.314 | 0.293 | 0.113 |
| 16 | rs12444979 | T | C | 0.149 | | 1124 | 10/147/405 | 0.262 | 0.253 | 0.506 |
| 16 | rs9939609 | A | T | 0.423 | | 1128 | 103/271/190 | 0.481 | 0.488 | 0.730 |
| 16 | rs12324955 | A | G | 0.276 | | 1134 | 38/237/292 | 0.418 | 0.400 | 0.295 |
| 18 | rs1398217 | G | C | 0.416 | | 1124 | 86/296/180 | 0.527 | 0.486 | 0.056 |
| 19 | rs2252673 | G | C | 0.216 | | 1132 | 30/185/351 | 0.327 | 0.339 | 0.386 |
| 20 | rs1073768 | A | G | 0.477 | | 1128 | 127/284/153 | 0.504 | 0.499 | 0.866 |
| 22 | rs4633 | C | T | 0.500 | | 1134 | 136/295/136 | 0.520 | 0.500 | 0.356 |
| 23 | rs5930973 | A | G | 0.054 | | 1122 | 2/57/502 | 0.102 | 0.103 | 0.675 |
| 23 | rs3092921 | T | C | 0.076 | | 1136 | 4/78/486 | 0.137 | 0.140 | 0.552 |

* minor allele homozygotes / heterozygotes / major allele homozygotes
