# Supplementary material for: Candidate Genes for Age at Menarche Are Associated With Uterine Leiomyoma
Source: Front Genet. 2021 Jan 22;11:512940. doi: 10.3389/fgene.2020.512940 (PMC7863975; doi:10.3389/fgene.2020.512940)
Supplement: Supplementary file 1 [file Data_Sheet_1.zip › SupMaterial 16-12-2020/Sup_Table_6.docx]

Supplementary Table 6 The data on the allele and genotype frequencies of the 52 SNPs in the control group.

| Chr | SNP | Minor allele | | Major allele | | Minor allele frequency | | Number of the studied chromosomes | | | Genotype distribution* | | Ho | | He | Р HWE |
| --- | --- | --- | --- | --- | --- | --- | --- | --- | --- | --- | --- | --- | --- | --- | --- | --- |
| 1 | rs1514175 | T | | C | | 0.381 | | 1958 | | | 144/457/378 | | 0.467 | | 0.471 | 0.786 |
| 1 | rs466639 | T | | C | | 0.123 | | 1958 | | | 16/209/754 | | 0.214 | | 0.216 | 0.767 |
| 1 | rs7538038 | G | | A | | 0.214 | | 1960 | | | 42/336/602 | | 0.343 | | 0.337 | 0.635 |
| 2 | rs713586 | C | | T | | 0.439 | | 1954 | | | 185/487/305 | | 0.499 | | 0.493 | 0.745 |
| 2 | rs2164808 | A | | G | | 0.470 | | 1956 | | | 204/512/262 | | 0.524 | | 0.498 | 0.124 |
| 2 | rs7589318 | A | | G | | 0.305 | | 1954 | | | 85/426/466 | | 0.436 | | 0.424 | 0.407 |
| 2 | rs4374421 | C | | T | | 0.313 | | 1892 | | | 87/418/441 | | 0.442 | | 0.430 | 0.449 |
| 2 | rs7579411 | T | | C | | 0.450 | | 1948 | | | 188/501/285 | | 0.514 | | 0.495 | 0.244 |
| 2 | rs6729809 | C | | T | | 0.323 | | 1868 | | | 89/426/419 | | 0.456 | | 0.438 | 0.205 |
| 2 | rs4953616 | C | | T | | 0.279 | | 1954 | | | 62/422/493 | | 0.432 | | 0.403 | 0.026 |
| 2 | rs6732220 | G | | C | | 0.257 | | 1960 | | | 62/379/539 | | 0.387 | | 0.382 | 0.738 |
| 2 | rs4953655 | G | | A | | 0.248 | | 1958 | | | 63/360/556 | | 0.368 | | 0.373 | 0.668 |
| 2 | rs887912 | A | | G | | 0.250 | | 1850 | | | 57/348/520 | | 0.376 | | 0.375 | 1.000 |
| 2 | rs12617311 | A | | G | | 0.328 | | 1954 | | | 107/427/443 | | 0.437 | | 0.441 | 0.772 |
| 3 | rs6438424 | C | | A | | 0.472 | | 1946 | | | 222/474/277 | | 0.487 | | 0.498 | 0.480 |
| 4 | rs2013573 | A | | G | | 0.188 | | 1956 | | | 23/322/633 | | 0.329 | | 0.306 | 0.016 |
| 4 | rs13111134 | A | | G | | 0.232 | | 1954 | | | 41/372/564 | | 0.381 | | 0.357 | 0.039 |
| 4 | rs222003 | C | | G | | 0.068 | | 1958 | | | 3/127/849 | | 0.130 | | 0.127 | 0.615 |
| 4 | rs222020 | C | | T | | 0.113 | | 1958 | | | 10/202/767 | | 0.206 | | 0.201 | 0.524 |
| 4 | rs3756261 | G | | A | | 0.073 | | 1938 | | | 2/137/830 | | 0.141 | | 0.135 | 0.227 |
| 5 | rs757647 | T | | C | | 0.241 | | 1944 | | | 51/367/554 | | 0.378 | | 0.366 | 0.381 |
| 6 | rs7766109 | G | | A | | 0.463 | | 1960 | | | 200/507/273 | | 0.517 | | 0.497 | 0.222 |
| 6 | rs4946651 | A | | G | | 0.428 | | 1958 | | | 177/483/319 | | 0.493 | | 0.490 | 0.845 |
| 6 | rs7759938 | C | | T | | 0.300 | | 1956 | | | 78/431/469 | | 0.441 | | 0.420 | 0.148 |
| 6 | rs314280 | T | | C | | 0.406 | | 1892 | | | 141/486/319 | | 0.514 | | 0.482 | 0.051 |
| 6 | rs314276 | A | | C | | 0.326 | | 1916 | | | 96/433/429 | | 0.452 | | 0.440 | 0.419 |
| 6 | rs3020394 | | G | | A | | 0.296 | | 1962 | 95/391/495 | | 0.399 | | 0.417 | | 0.169 |
| 6 | rs1884051 | | G | | A | | 0.292 | | 1960 | 93/387/500 | | 0.395 | | 0.414 | | 0.164 |
| 6 | rs7753051 | | C | | T | | 0.311 | | 1958 | 80/449/450 | | 0.459 | | 0.429 | | 0.031 |
| 7 | rs1079866 | | C | | G | | 0.174 | | 1959 | 27/286/666 | | 0.292 | | 0.287 | | 0.656 |
| 8 | rs2288696 | | T | | C | | 0.204 | | 1962 | 36/329/616 | | 0.335 | | 0.325 | | 0.377 |
| 9 | rs2090409 | | T | | G | | 0.372 | | 1838 | 131/421/367 | | 0.458 | | 0.467 | | 0.572 |
| 9 | rs10980926 | | A | | G | | 0.315 | | 1956 | 91/434/453 | | 0.444 | | 0.432 | | 0.415 |
| 9 | rs10441737 | | C | | T | | 0.334 | | 1804 | 92/418/392 | | 0.463 | | 0.445 | | 0.231 |
| 11 | rs10769908 | | C | | T | | 0.475 | | 1934 | 210/499/258 | | 0.516 | | 0.499 | | 0.302 |
| 11 | rs555621 | | G | | A | | 0.407 | | 1952 | 152/490/334 | | 0.502 | | 0.483 | | 0.233 |
| 11 | rs11031010 | | A | | C | | 0.131 | | 1939 | 17/219/733 | | 0.226 | | 0.227 | | 0.887 |
| 11 | rs1782507 | | C | | A | | 0.348 | | 1954 | 107/466/404 | | 0.477 | | 0.454 | | 0.121 |
| 11 | rs6589964 | | A | | C | | 0.464 | | 1956 | 211/486/281 | | 0.497 | | 0.497 | | 1.000 |
| 12 | rs1544410 | | A | | G | | 0.369 | | 1958 | 134/455/390 | | 0.465 | | 0.466 | | 0.945 |
| 14 | rs999460 | | A | | G | | 0.346 | | 1960 | 111/456/413 | | 0.465 | | 0.453 | | 0.398 |
| 14 | rs4986938 | | A | | G | | 0.344 | | 1954 | 100/473/404 | | 0.484 | | 0.452 | | 0.028 |
| 15 | rs2241423 | | A | | G | | 0.184 | | 1948 | 37/285/652 | | 0.293 | | 0.301 | | 0.395 |
| 16 | rs12444979 | | T | | C | | 0.148 | | 1958 | 25/240/714 | | 0.245 | | 0.252 | | 0.375 |
| 16 | rs9939609 | | A | | T | | 0.438 | | 1960 | 178/503/299 | | 0.513 | | 0.492 | | 0.195 |
| 16 | rs12324955 | | A | | G | | 0.283 | | 1962 | 86/384/511 | | 0.391 | | 0.406 | | 0.271 |
| 18 | rs1398217 | | G | | C | | 0.421 | | 1950 | 164/492/319 | | 0.505 | | 0.487 | | 0.293 |
| 19 | rs2252673 | | G | | C | | 0.204 | | 1952 | 40/319/617 | | 0.327 | | 0.325 | | 0.922 |
| 20 | rs1073768 | | A | | G | | 0.479 | | 1954 | 220/495/262 | | 0.507 | | 0.499 | | 0.654 |
| 22 | rs4633 | | C | | T | | 0.493 | | 1960 | 249/468/263 | | 0.478 | | 0.500 | | 0.160 |
| 23 | rs5930973 | | A | | G | | 0.055 | | 1926 | 6/93/864 | | 0.097 | | 0.103 | | 0.058 |
| 23 | rs3092921 | | T | | C | | 0.080 | | 1956 | 3/150/825 | | 0.153 | | 0.147 | | 0.195 |

* minor allele homozygotes / heterozygotes / major allele homozygotes
