# Supplementary material for: Candidate Genes for Age at Menarche Are Associated With Uterine Leiomyoma
Source: Front Genet. 2021 Jan 22;11:512940. doi: 10.3389/fgene.2020.512940 (PMC7863975; doi:10.3389/fgene.2020.512940)
Supplement: Supplementary file 1 [file Data_Sheet_1.zip › SupMaterial 16-12-2020/Sup_Table_2.docx]

Supplementary Table 2 The regulatory potential of the studied SNPs.

| Chr | SNP | Gene/Region | Database | | | | |
| --- | --- | --- | --- | --- | --- | --- | --- |
|  |  |  | HaploReg | SNP FuncPred | RegulomeDB | rSNPBase | rSNPs MAPPER |
| 1 | rs1514175 | *TNNI3K* | Enh 3 tis,2 motifs |  |  |  | 2 TFBSs |
| 1 | rs466639 | *RXRG* | Enh 12 tis,3 motifs |  |  | Prox reg, RNA BP reg | 1 TFBS |
| 1 | rs7538038 | *KISS1* | Enh 4 tis,2 motifs |  | 4 |  | 1 TFBS |
| 2 | rs713586 | *RBJ* | 3 motifs |  | 1f | Dist reg |  |
| 2 | rs2164808 | *POMC* | 2 motifs | RP=0.334 |  | Prox reg, RNA BP reg | 1 TFBS |
| 2 | rs7589318 | *POMC* | 2 motifs | miRNA | 5 | Prox reg | 2 TFBSs |
| 2 | rs4374421 | *LHCGR* | 2 motifs | RP=0.044 |  | RNA BP reg | 1 TFBS |
| 2 | rs7579411 | *LHCGR* |  |  |  | RNA BP reg | 3 TFBSs |
| 2 | rs6729809 | *LHCGR* | DNAse 4 tis, 2 PB, 2 motifs |  | 4 | RNA BP reg | 1 TFBS |
| 2 | rs4953616 | *LHCGR* | Enh 12 tis, DNAse 1 tis,1 motif |  | 5 | RNA BP reg |  |
| 2 | rs6732220 | *FSHR* |  |  |  |  | 1 TFBS |
| 2 | rs4953655 | *FSHR* | 4 motifs | RP=0.046 |  | Prox reg | 2 TFBSs |
| 2 | rs887912 | *FANCL* | 2 motifs |  | 6 |  | 3 TFBSs |
| 2 | rs12617311 | *PLCL1* | Prom 1 tis, 2 motifs |  |  |  | 3 TFBSs |
| 3 | rs6438424 | *3q13.32* | motif |  | 3a | Dist reg |  |
| 4 | rs2013573 | *UGT2B4* | 3 motifs | RP=0.177 | 5 |  | 1 TFBS |
| 4 | rs13111134 | *UGT2B4* | 4 motifs |  |  |  | 1 TFBS |
| 4 | rs222003 | *GC* | 3 motifs |  | 6 |  | 3 TFBSs |
| 4 | rs222020 | *GC* | Prom 1 tis, 5 motifs |  |  | Prox reg |  |
| 4 | rs3756261 | *EGF* | motif | TFBS | 6 | Prox reg, Dist reg |  |
| 5 | rs757647 | *KDM3B* | Enh 7 tis, DNAse 7 tis, 1 PB, 1 motif |  | 1f | Prox reg, Dist reg | 1 TFBS |
| 6 | rs7766109 | *F13A1* | Enh 5 tis, DNAse 1 tis,2 motifs | RP=0.107 | 5 | RNA BP reg | 2 TFBSs |
| 6 | rs4946651 | *LIN28B* |  | RP=0.027 |  |  |  |
| 6 | rs7759938 | *LIN28B* | Enh 2 tis |  | 5 | Dist reg |  |
| 6 | rs314280 | *LIN28B* | Enh 1 tis, Prom 15 tis, DNAse 11 tis, 5 PB | RP= 0.098, TFBS | 4 | Prox reg |  |
| 6 | rs314276 | *LIN28B* | Enh 2 tis, Prom 5 tis, 3 motifs | TFBS | 6 | RNA BP reg | 2 TFBSs |
| 6 | rs3020394 | *ESR1* | Enh 7 tis, DNAse 1 tis |  |  | RNA BP reg | 1 TFBS |
| 6 | rs1884051 | *ESR1* | Enh 6 tis, 3 motifs |  | 5 | RNA BP reg | 3 TFBSs |
| 6 | rs7753051 | *IGF2R* | 3 motifs |  | 5 |  |  |
| 7 | rs1079866 | *INHBA* | 2 motifs |  | 6 |  | 2 TFBSs |
| 8 | rs2288696 | *FGFR1* | Enh 5 tis, DNAse 2 tis, 1 motif | RP=0.026 | 5 | Prox reg, RNA BP reg | 1 TFBS |
| 9 | rs2090409 | *TMEM38B* | 3 motifs |  |  |  | 6 TFBSs |
| 9 | rs10980926 | *ZNF483* | 9 motifs |  |  | RNA BP reg | 5 TFBSs |
| 9 | rs10441737 | *ZNF483* | 9 motifs |  | 6 | RNA BP reg | 9 TFBSs |
| 11 | rs10769908 | *STK33* | 13 motifs | RP=0.046 | 6 | RNA BP reg | 10 TFBSs |
| 11 | rs555621 | *FSHB* |  |  | 5 |  | 2 TFBSs |
| 11 | rs11031010 | *FSHB* | Prom 1 tis, 4 motifs |  |  | Dist reg | 2 TFBSs |
| 11 | rs1782507 | *FSHB* | 5 motifs |  |  |  | 1 TFBS |
| 11 | rs6589964 | *BSX* | DNAse 1 tis, 2 motifs |  | 6 | Dist reg | 5 TFBSs |
| 12 | rs1544410 | *VDR* | 4 motifs | RP=0.084 | 5 | RNA BP reg |  |
| 14 | rs999460 | *NKX2-1* | Prom 6 tis, DNAse 7 tis, 5 motifs | TFBS | 2b | Prox reg | 6 TFBSs |
| 14 | rs4986938 | *ESR2* | Enh 17 tis, Prom 1 tis, DNAse 6 tis, 5 motifs | miRNA | 2b | Prox reg, Dist reg, RNA BP reg | 3 TFBSs |
| 15 | rs2241423 | *MAP2K5* | DNAse 1 tis | RP=0.144 | 5 | RNA BP reg | 1 TFBS |
| 16 | rs12444979 | *GPRC5B* | 1 motif |  |  |  |  |
| 16 | rs9939609 | *FTO* | 2 motifs | RP=0.175 |  | RNA BP reg |  |
| 16 | rs12324955 | *FTO* | Enh 1 tis, 1 motif |  |  | Prox reg, RNA BP reg | 3 TFBSs |
| 18 | rs1398217 | *SKOR2* | 18 motifs | RP=0.036 | 6 |  | 3 TFBSs |
| 19 | rs2252673 | *INSR* | DNAse 2 tis, 1 PB, 2 motifs | RP=0.096 | 2b | RNA BP reg | 1 TFBS |
| 20 | rs1073768 | *GHRH* | DNAse 1 tis, 2 motifs |  | 5 |  | 1 TFBS |
| 22 | rs4633 | *COMT* | Enh 24 tis, Prom 2 tis, DNAse 23 tis, 7 PB , 7 motifs | RP=0.420 | 2b | Prox reg, Dist reg, RNA BP reg | 11 TFBSs |
| X | rs5930973 | *CD40LG* | Enh 1 tis, Prom 2 tis, 1 motifs |  |  |  | 1 TFBS |
| X | rs3092921 | *CD40LG* | Enh 2 tis, Prom 1 tis, 1 motifs |  | 5 |  | 3 TFBSs |

*Note:* HaploReg (v4.1) (http://archive.broadinstitute.org/mammals/haploreg/haploreg.php) (Core 25-state model using 12 imputed marks): Enh – Enhancer, Prom – Promoter, DNAse – DNase hypersensitive regions; PB - Protein-binding sites; motifs - Regulatory motifs changed; tis - tissues;

SNP FuncPred (https://snpinfo.niehs.nih.gov/snpinfo/snpfunc.html): RP - Regulatory Potential Score; TFBS - Transcription Factor Binding Sites

RegulomeDB (version 1.1) (http://rsnp.psych.ac.cn/index.do): представлены regulatory scores - 1a (eQTL + TF binding + matched TF motif + matched DNase Footprint + DNase peak), 1b (eQTL + TF binding + any motif + DNase Footprint + DNase peak), 1c (eQTL + TF binding + matched TF motif + DNase peak), 1d (eQTL + TF binding + any motif + DNase peak), 1e (eQTL + TF binding + matched TF motif), 1f (eQTL + TF binding / DNase peak), 2a (TF binding + matched TF motif + matched DNase Footprint + DNase peak), 2b (TF binding + any motif + DNase Footprint + DNase peak), 2c (TF binding + matched TF motif + DNase peak), 3a (TF binding + any motif + DNase peak), 3b (TF binding + matched TF motif), 4 (TF binding + DNase peak), 5 (TF binding or DNase peak), 6 (other);

rSNPBase (http://rsnp.psych.ac.cn/index.do): Prox reg - proximal transcriptional regulation, Dist reg - distal transcriptional regulation, RNA BP reg - RNA binding protein mediated regulation;

rSNPs MAPPER (http://genome.ufl.edu/mapper/mapper-main): TFBS - Transcription Factor Binding Sites.
