# Supplementary material for: Candidate Genes for Age at Menarche Are Associated With Uterine Leiomyoma
Source: Front Genet. 2021 Jan 22;11:512940. doi: 10.3389/fgene.2020.512940 (PMC7863975; doi:10.3389/fgene.2020.512940)
Supplement: Supplementary file 1 [file Data_Sheet_1.zip › SupMaterial 16-12-2020/Sup_Table_8.docx]

Supplementary Table 8 Cross-validation of the most significant models of SNP-SNP interactions associated with uterine leiomyoma.

| N | Models of SNP х SNP interactions | OR (95%CI) | Test. Bal. Acc. | S_e_ | S_p_ | CVC |
| --- | --- | --- | --- | --- | --- | --- |
| Two-locus models | | | | | | |
| 1 | rs11031010 *FSHB* x rs2241423 *MAP2K5* | 1.88 (1.39-2.55) | 52.70 | 88.58 | 19.57 | 10/10 |
| 2 | rs222020 *GC* x rs4374421 *LHCGR* | 1.61 (1.28-2.02) | 54.91 | 72.58 | 37.82 | 10/10 |
| 3 | rs314276 *LIN28B* x rs2164808 *POMC* | 1.47 (1.18-1.83) | 51.17 | 68.72 | 40.16 | 10/10 |
| Three-locus models | | | | | | |
| 1 | rs1782507 *FSHB*  x rs1884051 *ESR1* x rs7766109 *F13A1* | 1.90 (1.52-2.36) | 54.00 | 41.65 | 72.68 | 10/10 |
| 2 | rs1782507 *FSHB* x rs3020394 *ESR1* x rs7766109 *F13A1* | 1.87 (1.51-2.32) | 53.83 | 43.59 | 70.74 | 10/10 |
| 3 | rs11031010 *FSHB* x rs314276 *LIN28B* x rs2164808 *POMC* | 2.16 (1.71-2.74) | 55.87 | 78.21 | 37.61 | 10/10 |
| 4 | rs314276 *LIN28B* x rs2164808 *POMC* x rs7753051 *IGF2R* | 2.06 (1.66-2.56) | 57.51 | 66.78 | 50.66 | 10/10 |
| 5 | rs11031010 *FSHB* x rs2164808 *POMC* x rs7753051 *IGF2R* | 1.96 (1.59-2.41) | 57.40 | 52.72 | 63.71 | 10/10 |
| Four-locus models | | | | | | |
| 1 | rs314276 *LIN28B* x rs1782507 *FSHB* x rs1544410 *VDR* x rs7589318 *POMC* | 2.69 (2.16-3.34) | 54.70 | 68.72 | 55.05 | 10/10 |
| 2 | rs12324955 *FTO* x rs10980926 *ZNF483*  x rs555621 *FSHB* x rs4953655 *FSHR* | 2.62 (2.11-3.25) | 55.15 | 50.26 | 72.17 | 10/10 |
| 3 | rs4946651 *LIN28B* x rs7589318 *POMC*  x rs10769908 *STK33* x rs6729809 *LHCGR* | 2.29 (1.86-2.84) | 53.08 | 53.78 | 66.36 | 10/10 |

- The results were obtained using the GMDR method with adjustment for сovariates;
- OR (95%CI) – odds ratio at 95% confidence interval, р – significance level;
- Test. Bal. Acc. – Testing balanced accuracy, S_e_ – model sensitivity , S_p_ – model specificity;
- CVC - cross validation consistency;
- all models were validated through the permutation test with adjustment for covariates. The permutation test included 1000 permutations at 10-fold cross-validation that ensured р_рerm_<0.001; the reproducibility of the models (CVC) after the test was 100%.
