# Supplementary material for: Candidate Genes for Age at Menarche Are Associated With Uterine Leiomyoma
Source: Front Genet. 2021 Jan 22;11:512940. doi: 10.3389/fgene.2020.512940 (PMC7863975; doi:10.3389/fgene.2020.512940)
Supplement: Supplementary file 1 [file Data_Sheet_1.zip › SupMaterial 16-12-2020/Sup_Table_9.DOC]

Supplementary Table 9 Associations of genotype combinations with uterine leiomyoma within the most significant models of gene-gene interactions.

| N  Models | Genotype combinations | | Number of cases | | Number of controls | | *beta* | | P | | Risk,  High/Low |
| --- | --- | --- | --- | --- | --- | --- | --- | --- | --- | --- | --- |
| Two-locus models | | | | | | | | | | | |
| 1 | rs11031010 CC x rs2241423 GG | | 323 | | 498 | | 0.23 | | 0.0326 | | H |
|  | rs11031010 CA x rs2241423 GG | | 50 | | 147 | | -0.66 | | **0.0002** | | L |
|  | rs11031010 CC x rs2241423 AA | | 8 | | 31 | | -0.90 | | 0.0294 | | L |
| 2 | rs222020 TT x rs4374421 TC | | 145 | | 335 | | -0.44 | | **0.0003** | | L |
|  | rs222020 TC x rs4374421 CC | | 7 | | 29 | | -0.92 | | 0.0335 | | L |
| 3 | rs314276 CC x rs2164808 GG | | 96 | | 113 | | 0.46 | | 0.0027 | | H |
|  | rs314276 CA x rs2164808 GG | | 37 | | 124 | | -0.69 | | 0.0005 | | L |
|  | rs314276 AA x rs2164808 GA | | 18 | | 52 | | -0.58 | | 0.0428 | | L |
| Three-locus models | | | | | | | | | | | |
| 1 | rs1782507 AC x rs1884051 AG x rs7766109 AA | | 49 | | 39 | | 0.82 | | **0.0003** | | H |
|  | rs1782507 CC x rs1884051 AA x rs7766109 AG | | 28 | | 27 | | 0.70 | | 0.0130 | | H |
|  | rs1782507 AA x rs1884051 AG x rs7766109 AG | | 34 | | 88 | | -0.44 | | 0.0377 | | L |
|  | rs1782507 AA x rs1884051 AG x rs7766109 GG | | 30 | | 25 | | 0.85 | | 0.0026 | | H |
|  | rs1782507 CC x rs1884051 AG x rs7766109 GG | | 10 | | 2 | | 2.38 | | 0.0024 | | H |
| 2 | rs1782507 AC x rs3020394 AG x rs7766109 AA | | 49 | | 43 | | 0.71 | | 0.0014 | | H |
|  | rs1782507 CC x rs3020394 AA x rs7766109 AG | | 27 | | 26 | | 0.71 | | 0.0135 | | H |
|  | rs1782507 AA x rs3020394 AG x rs7766109 AG | | 35 | | 91 | | -0.45 | | 0.0331 | | L |
|  | rs1782507 AA x rs3020394 AG x rs7766109 GG | | 30 | | 24 | | 0.88 | | 0.0020 | | H |
|  | rs1782507 CC x rs3020394 AG x rs7766109 GG | 10 | | 2 | | 2.38 | | 0.0024 | | H | |
| 3 | rs11031010 CC x rs314276 CC x rs2164808 GG | 73 | | 92 | | 0.38 | | 0.0276 | | H | |
|  | rs11031010 CC x rs314276 CA x rs2164808 GG | 26 | | 98 | | -0.79 | | 0.0006 | | L | |
|  | rs11031010 CC x rs314276 CC x rs2164808 GA | 128 | | 177 | | 0.32 | | 0.0176 | | H | |
|  | rs11031010 CA x rs314276 CC x rs2164808 GA | 21 | | 66 | | -0.65 | | 0.0131 | | L | |
|  | rs11031010 CA x rs314276 CA x rs2164808 AA | 10 | | 34 | | -0.80 | | 0.0312 | | L | |
| 4 | rs314276 CC x rs2164808 GA x rs7753051 TT | 86 | | 112 | | 0.32 | | 0.0458 | | H | |
|  | rs314276 CA x rs2164808 GA x rs7753051 TT | 77 | | 101 | | 0.33 | | 0.0499 | | H | |
|  | rs314276 CC x rs2164808 GG x rs7753051 TC | 50 | | 50 | | 0.62 | | 0.0040 | | H | |
|  | rs314276 CA x rs2164808 GG x rs7753051 TC | 13 | | 57 | | -0.89 | | 0.0049 | | L | |
|  | rs314276 CA x rs2164808 GA x rs7753051 TC | 37 | | 93 | | -0.48 | | 0.0210 | | L | |
|  | rs314276 AA x rs2164808 GA x rs7753051 TC | 8 | | 29 | | -0.87 | | 0.0352 | | L | |
| 5 | rs11031010 CC x rs2164808 GA x rs7753051 TT | 147 | | 176 | | 0.49 | | **0.0002** | | H | |
|  | rs11031010 CA x rs2164808 GG x rs7753051 TC | 20 | | 10 | | 1.20 | | 0.0025 | | H | |
|  | rs11031010 CC x rs2164808 GA x rs7753051 TC | 81 | | 185 | | -0.35 | | 0.0177 | | L | |
|  | rs11031010 CA x rs2164808 GA x rs7753051 TC | 14 | | 49 | | -0.72 | | 0.0229 | | L | |
| Four-locus models | | | | | | | | | | | |
| 1 | rs314276 CA x rs1782507 AA x rs1544410 GG x rs7589318 GG | 4 | | 33 | | -1.49 | | 0.0056 | | L | |
|  | rs314276 CC x rs1782507 AC x rs1544410 GG x rs7589318 GG | 31 | | 31 | | 0.60 | | 0.0258 | | H | |
|  | rs314276 CC x rs1782507 CC x rs1544410 GG x rs7589318 GG | 10 | | 5 | | 1.36 | | 0.0182 | | H | |
|  | rs314276 CA x rs1782507 AC x rs1544410 GA x rs7589318 GG | 16 | | 57 | | -0.80 | | 0.0065 | | L | |
|  | rs314276 CC x rs1782507 AA x rs1544410 AA x rs7589318 GG | 11 | | 5 | | 1.37 | | 0.0132 | | H | |
| 2 | rs314276 CA x rs1782507 AA x rs1544410 AA x rs7589318 GG | 9 | | 4 | | 1.49 | | 0.0159 | | H | |
|  | rs314276 CC x rs1782507 AC x rs1544410 AA x rs7589318 GG | 16 | | 12 | | 0.87 | | 0.0278 | | H | |
|  | rs314276 CA x rs1782507 AA x rs1544410 GA x rs7589318GA | 27 | | 25 | | 0.72 | | 0.0132 | | H | |
|  | rs314276 CA x rs1782507 AA x rs1544410 AA x rs7589318 GA | 4 | | 26 | | -1.43 | | 0.0093 | | L | |
|  | rs314276 CA x rs1782507 AC x rs1544410 GG x rs7589318 AA | 2 | | 15 | | -1.56 | | 0.0401 | | L | |
|  | rs12324955 GG x rs10980926 GG x rs555621 AA x rs4953655 AA | 44 | | 37 | | 0.77 | | 0.0012 | | H | |
|  | rs12324955 AA x rs10980926 GG x rs555621 AA x rs4953655 AA | 9 | | 5 | | 1.12 | | 0.0498 | | H | |
|  | rs12324955 GG x rs10980926 GG x rs555621 AG x rs4953655 AA | 47 | | 63 | | 0.43 | | 0.0391 | | H | |
|  | rs12324955 GA x rs10980926 GG x rs555621 AG x rs4953655 AA | 38 | | 40 | | 0.57 | | 0.0184 | | H | |
|  | rs12324955 GG x rs10980926 AA x rs555621 AA x rs4953655 AG | 1 | | 12 | | -2.08 | | 0.0470 | | L | |
|  | rs12324955 GA x rs10980926 AA x rs555621 AA x rs4953655 AG | 9 | | 3 | | 1.87 | | 0.0057 | | H | |
|  | rs12324955 GA x rs10980926 GG x rs555621 GG x rs4953655 AG | 12 | | 10 | | 0.89 | | 0.0452 | | H | |
| 3 | rs4946651 GG x rs7589318 AA x rs10769908 TC x rs6729809 TT | 11 | | 7 | | 0.98 | | 0.0463 | | H | |
|  | rs4946651 GA x rs7589318 AA x rs10769908 TC x rs6729809 TT | 10 | | 8 | | 0.97 | | 0.0469 | | H | |
|  | rs4946651 GG x rs7589318 GG x rs10769908 CC x rs6729809 TT | 17 | | 11 | | 1.20 | | 0.0027 | | H | |
|  | rs4946651 GG x rs7589318 GG x rs10769908 TT x rs6729809 TC | 21 | | 12 | | 1.10 | | 0.0034 | | H | |
|  | rs4946651 GA x rs7589318 AA x rs10769908 TC x rs6729809 TC | 8 | | 2 | | 2.25 | | 0.0052 | | H | |
|  | rs4946651 GA x rs7589318 GG x rs10769908 CC x rs6729809 TC | 16 | | 12 | | 0.88 | | 0.0278 | | H | |
|  | rs4946651 GG x rs7589318 GG x rs10769908 TC x rs6729809 CC | 9 | | 4 | | 1.40 | | 0.0235 | | H | |

The results were obtained by the MB-MDR analysis with adjustment for сovariates;

*beta* – coefficient of logistic regression;
