# Supplementary material for: Candidate Genes for Age at Menarche Are Associated With Uterine Leiomyoma
Source: Front Genet. 2021 Jan 22;11:512940. doi: 10.3389/fgene.2020.512940 (PMC7863975; doi:10.3389/fgene.2020.512940)
Supplement: Supplementary file 1 [file Data_Sheet_1.zip › SupMaterial 16-12-2020/Sup_Table_16.DOC]

Supplementary Table 16 cis-eQTL values in peripheral blood of SNPs in high LD (r^2^≥0.80) with the UL-associated polymorphisms determined in the present study (according to the Blood eQTL browser) (http://genenetwork.nl/bloodeqtlbrowser/) (р <5.0е-5, FDR<0.05).

| Chr | Lead SNP | Position lead SNP(hg38) | Proxy SNP | Position proxy SNP (hg38) | Distance (bp) between proxy and lead SNP | r^2^ between lead and proxy | D’ between lead and proxy | Gene Symbol | Z-score | P | FDR |
| --- | --- | --- | --- | --- | --- | --- | --- | --- | --- | --- | --- |
| 2 | rs2164808 | 25154307 | rs4665765 | 25139646 | 14661 | 0.99 | 1 | *DNAJC27* | -4.08 | 4.52E-05 | 0.02 |
| 6 | rs7753051 | 160107025 | rs2297370 | 160103741 | 3284 | 0.9 | 0.98 | *SLC22A1* | -4.99 | 5.93E-07 | 0 |
| 6 | rs7753051 | 160107025 | rs2297371 | 160104155 | 2870 | 0.91 | 0.99 | *SLC22A1* | -4.99 | 6.01E-07 | 0 |
| 6 | rs7753051 | 160107025 | rs9347385 | 160108389 | -1364 | 0.94 | 1 | *SLC22A1* | -4.91 | 8.89E-07 | 0 |
| 6 | rs7753051 | 160107025 | rs1479355 | 160109594 | -2569 | 0.98 | 0.99 | *SLC22A1* | -4.96 | 6.88E-07 | 0 |
| 6 | rs7753051 | 160107025 | rs9365132 | 160110411 | -3386 | 0.91 | 0.98 | *SLC22A1* | -4.93 | 8.09E-07 | 0 |
| 6 | rs7753051 | 160107025 | rs9365133 | 160110743 | -3718 | 0.92 | 0.98 | *SLC22A1* | -4.93 | 8.05E-07 | 0 |
| 6 | rs7753051 | 160107025 | rs9346808 | 160111620 | -4595 | 0.98 | 0.99 | *SLC22A1* | -4.9 | 9.39E-07 | 0 |
| 6 | rs7753051 | 160107025 | rs6935207 | 160120141 | -13116 | 0.82 | 0.96 | *SLC22A1* | -4.7 | 2.65E-06 | 0 |
| 6 | rs7753051 | 160107025 | rs4709400 | 160122578 | -15553 | 0.82 | 0.96 | *SLC22A1* | -4.68 | 2.86E-06 | 0 |
| 9 | rs10980926 | 111531354 | rs10441737 | 111539305 | -7951 | 0.98 | 1 | *KIAA0368* | -6 | 1.94E-09 | 0 |
| 15 | rs2241423 | 67794500 | rs7172055 | 67645996 | 148504 | 0.8 | 0.95 | *MAP2K5* | -12.73 | 4.05E-37 | 0 |
| 15 | rs2241423 | 67794500 | rs11857017 | 67680570 | 113930 | 0.8 | 0.95 | *MAP2K5* | -12.61 | 1.94E-36 | 0 |
| 15 | rs2241423 | 67794500 | rs13380201 | 67681327 | 113173 | 0.8 | 0.95 | *MAP2K5* | -12.61 | 1.91E-36 | 0 |
| 15 | rs2241423 | 67794500 | rs4331293 | 67695463 | 99037 | 0.8 | 0.95 | *MAP2K5* | -12.58 | 2.67E-36 | 0 |
| 15 | rs2241423 | 67794500 | rs4776964 | 67724617 | 69883 | 0.8 | 0.95 | *MAP2K5* | -12.58 | 2.73E-36 | 0 |
| 15 | rs2241423 | 67794500 | rs8028313 | 67750719 | 43781 | 0.91 | 0.96 | *MAP2K5* | -10.5 | 8.53E-26 | 0 |
| 15 | rs2241423 | 67794500 | rs3784707 | 67751540 | 42960 | 0.85 | 0.98 | *MAP2K5* | -11.45 | 2.43E-30 | 0 |
| 15 | rs2241423 | 67794500 | rs4548812 | 67768214 | 26286 | 0.99 | 1 | *MAP2K5* | -10.36 | 3.94E-25 | 0 |
| 15 | rs2241423 | 67794500 | rs3784710 | 67780120 | 14380 | 0.96 | 0.99 | *MAP2K5* | -9.69 | 3.46E-22 | 0 |
| 15 | rs2241423 | 67794500 | rs8025790 | 67784061 | 10439 | 0.97 | 0.99 | *MAP2K5* | -10.36 | 3.60E-25 | 0 |
| 15 | rs2241423 | 67794500 | rs8030456 | 67784518 | 9982 | 0.99 | 0.99 | *MAP2K5* | -10.4 | 2.47E-25 | 0 |
| 15 | rs2241423 | 67794500 | rs16951275 | 67784830 | 9670 | 0.99 | 0.99 | *MAP2K5* | -10.42 | 1.96E-25 | 0 |
| 15 | rs2241423 | 67794500 | rs7175517 | 67785292 | 9208 | 0.99 | 0.99 | *MAP2K5* | -10.42 | 1.96E-25 | 0 |
| 15 | rs2241423 | 67794500 | rs2241420 | 67790478 | 4022 | 0.99 | 1 | *MAP2K5* | -10.29 | 8.10E-25 | 0 |
| 15 | rs2241423 | 67794500 | rs4776375 | 67793217 | 1283 | 1 | 1 | *MAP2K5* | -10.42 | 2.05E-25 | 0 |
| 15 | rs2241423 | 67794500 | rs16951304 | 67797280 | -2780 | 0.9 | 1 | *MAP2K5* | -11.3 | 1.29E-29 | 0 |
| 15 | rs2241423 | 67794500 | rs2127163 | 67798504 | -4004 | 0.9 | 1 | *MAP2K5* | -11.33 | 9.32E-30 | 0 |
| 15 | rs2241423 | 67794500 | rs13329413 | 67804788 | -10288 | 0.91 | 1 | *MAP2K5* | -11.28 | 1.61E-29 | 0 |
| 15 | rs2241423 | 67794500 | rs2278076 | 67807262 | -12762 | 0.91 | 1 | *MAP2K5* | -11.32 | 1.11E-29 | 0 |
| 15 | rs2241423 | 67794500 | rs4776976 | 67810953 | -16453 | 0.92 | 0.96 | *MAP2K5* | -10.41 | 2.15E-25 | 0 |
| 15 | rs2241423 | 67794500 | rs13329567 | 67812029 | -17529 | 0.97 | 0.99 | *MAP2K5* | -10.41 | 2.15E-25 | 0 |
| 15 | rs2241423 | 67794500 | rs13329582 | 67812270 | -17770 | 0.97 | 0.99 | *MAP2K5* | -10.41 | 2.15E-25 | 0 |
| 15 | rs2241423 | 67794500 | rs4776981 | 67817136 | -22636 | 0.97 | 0.99 | *MAP2K5* | -10.41 | 2.14E-25 | 0 |
| 15 | rs2241423 | 67794500 | rs4776982 | 67822636 | -28136 | 0.96 | 0.98 | *MAP2K5* | -10.39 | 2.74E-25 | 0 |
| 15 | rs2241423 | 67794500 | rs4776987 | 67831577 | -37077 | 0.86 | 0.97 | *MAP2K5* | -11.22 | 3.08E-29 | 0 |
| 22 | rs4633 | 19962712 | rs4680 | 19963748 | -1036 | 0.99 | 1 | *COMT* | -4.27 | 1.95E-05 | 0.01 |
