# Supplementary material for: Candidate Genes for Age at Menarche Are Associated With Uterine Leiomyoma
Source: Front Genet. 2021 Jan 22;11:512940. doi: 10.3389/fgene.2020.512940 (PMC7863975; doi:10.3389/fgene.2020.512940)
Supplement: Supplementary file 1 [file Data_Sheet_1.zip › SupMaterial 16-12-2020/Sup_Table_17.DOC]

Supplementary table 17 The *cis*-eQTL effect of the 23 UL-associated SNPs in various tissues according to the Genotype-Tissue Expression (GTEx)*

| Chr | | SNP | Gene | Affected Gene Symbol | Ref | Alt | β | р | | Tissue |  | | | | | | | | | | | | | |
| --- | --- | --- | --- | --- | --- | --- | --- | --- | --- | --- | --- | --- | --- | --- | --- | --- | --- | --- | --- | --- | --- | --- | --- | --- |
| 2 | | rs2164808 | *POMC* | *RP11-509E16.1* | T | C | -0.23 | 0.000067 | | Esophagus - Mucosa |  | | | | | | | | | | | | | |
| 2 | | rs7589318 | *POMC* | *DNMT3A* | G | A | 0.24 | 4.3E-06 | | Esophagus - Mucosa |  | | | | | | | | | | | | | |
| 2 | | rs6729809 | *LHCGR* | *STON1-GTF2A1L* | C | T | 0.34 | 0.000023 | | Nerve - Tibial |  | | | | | | | | | | | | | |
| 2 | | rs4953655 | *FSHR* | *FSHR* | G | A | 0.39 | 1.80E-07 | | Testis |  | | | | | | | | | | | | | |
| 6 | | rs4946651 | *LIN28B* | *LIN28B* | A | G | -0.4 | 7.60E-08 | | Pituitary |  | | | | | | | | | | | | | |
|  | | rs4946651 |  | *LINC00577* | A | G | 0.58 | 1.6E-06 | | Brain - Cortex |  | | | | | | | | | | | | | |
|  | | rs4946651 |  | *LINC00577* | A | G | 0.48 | 2.2E-06 | | Brain - Putamen (basal ganglia) |  | | | | | | | | | | | | | |
| 6 | | rs7759938 | *LIN28B* | *LIN28B* | C | T | -0.5 | 1.30E-11 | | Pituitary |  | | | | | | | | | | | | | |
| 6 | | rs314280 | *LIN28B* | *LIN28B* | A | G | -0.4 | 7.60E-08 | | Pituitary |  | | | | | | | | | | | | | |
|  | | rs314280 |  | *LINC00577* | A | G | 0.58 | 1.6E-06 | | Brain - Cortex |  | | | | | | | | | | | | | |
|  | | rs314280 |  | *LINC00577* | A | G | 0.48 | 2.2E-06 | | Brain - Putamen (basal ganglia) |  | | | | | | | | | | | | | |
| 6 | | rs314276 | *LIN28B* | *LIN28B* | A | C | -0.5 | 9.40E-12 | | Pituitary |  | | | | | | | | | | | | | |
| 6 | | rs7753051 | *IGF2R* | *SLC22A1* | T | C | -0.31 | 4.00E-19 | | Whole Blood |  | | | | | | | | | | | | | |
|  | | rs7753051 |  | *SLC22A1* | T | C | -0.42 | 2.00E-17 | | Muscle - Skeletal |  | | | | | | | | | | | | | |
|  | | rs7753051 |  | *SLC22A1* | T | C | -0.44 | 3.50E-11 | | Lung |  | | | | | | | | | | | | | |
|  | | rs7753051 |  | *SLC22A1* | T | C | -0.33 | 2.30E-10 | | Thyroid |  | | | | | | | | | | | | | |
|  | | rs7753051 |  | *SLC22A1* | T | C | -0.33 | 3.80E-08 | | Adipose - Visceral (Omentum) |  | | | | | | | | | | | | | |
|  | | rs7753051 |  | *RP11-288H12.3* | T | C | 0.27 | 2.3E-06 | | Colon - Sigmoid |  | | | | | | | | | | | | | |
|  | | rs7753051 |  | *SLC22A1* | T | C | -0.35 | 4.3E-06 | | Esophagus - Muscularis |  | | | | | | | | | | | | | |
|  | | rs7753051 |  | *SLC22A1* | T | C | -0.42 | 0.000005 | | Testis |  | | | | | | | | | | | | | |
|  | | rs7753051 |  | *SLC22A1* | T | C | -0.35 | 5.6E-06 | | Colon - Transverse |  | | | | | | | | | | | | | |
|  | | rs7753051 |  | *SLC22A1* | T | C | -0.3 | 6.8E-06 | | Adipose - Subcutaneous |  | | | | | | | | | | | | | |
| 11 | | rs10769908 | *STK33* | *STK33* | C | T | -0.58 | 4.20E-33 | | Artery - Tibial |  | | | | | | | | | | | | | |
|  | | rs10769908 |  | *STK33* | C | T | -0.58 | 1.80E-17 | | Artery - Aorta |  | | | | | | | | | | | | | |
|  | | rs10769908 |  | *STK33* | C | T | -0.26 | 7.90E-14 | | Testis |  | | | | | | | | | | | | | |
|  | | rs10769908 |  | *STK33* | C | T | -0.17 | 1.20E-08 | | Muscle - Skeletal |  | | | | | | | | | | | | | |
|  | | rs10769908 |  | *TRIM66* | C | T | -0.22 | 5.30E-08 | | Skin - Sun Exposed (Lower leg) |  | | | | | | | | | | | | | |
|  | | rs10769908 |  | *TRIM66* | C | T | -0.23 | 7.80E-08 | | Thyroid |  | | | | | | | | | | | | | |
|  | | rs10769908 |  | *STK33* | C | T | -0.52 | 2.00E-07 | | Brain - Cerebellar Hemisphere |  | | | | | | | | | | | | | |
|  | | rs10769908 |  | *STK33* | C | T | -0.38 | 5.60E-07 | | Artery - Coronary |  | | | | | | | | | | | | | |
|  | | rs10769908 |  | *STK33* | C | T | -0.14 | 2.3E-06 | | Adipose - Visceral (Omentum) |  | | | | | | | | | | | | | |
|  | | rs10769908 |  | *STK33* | C | T | -0.45 | 2.4E-06 | | Brain - Cerebellum |  | | | | | | | | | | | | | |
|  | | rs10769908 |  | *TRIM66* | C | T | -0.24 | 8.4E-06 | | Breast - Mammary Tissue |  | | | | | | | | | | | | | |
|  | | rs10769908 |  | *TRIM66* | C | T | -0.18 | 0.000011 | | Skin - Not Sun Exposed (Suprapubic) |  | | | | | | | | | | | | | |
|  | | rs10769908 |  | *STK33* | C | T | -0.27 | 0.000035 | | Skin - Not Sun Exposed (Suprapubic) |  | | | | | | | | | | | | | |
| 11 | | rs555621 | *FSHB* | *ARL14EP* | T | C | 0.3 | 2.60E-11 | | Nerve - Tibial |  | | | | | | | | | | | | | |
|  | | rs555621 |  | *ARL14EP* | T | C | -0.21 | 1.50E-10 | | Whole Blood |  | | | | | | | | | | | | | |
|  | | rs555621 |  | *ARL14EP* | T | C | 0.35 | 5.00E-10 | | Testis |  | | | | | | | | | | | | | |
|  | | rs555621 |  | *ARL14EP* | T | C | 0.2 | 3.90E-07 | | Heart - Atrial Appendage |  | | | | | | | | | | | | | |
|  | | rs555621 |  | *ARL14EP* | T | C | 0.24 | 4.00E-07 | | Esophagus - Gastroesophageal Junction |  | | | | | | | | | | | | | |
|  | | rs555621 |  | *ARL14EP* | T | C | 0.21 | 0.000001 | | Esophagus - Muscularis |  | | | | | | | | | | | | | |
|  | | rs555621 |  | *ARL14EP* | T | C | 0.32 | 2.3E-06 | | Pancreas |  | | | | | | | | | | | | | |
|  | | rs555621 |  | *ARL14EP* | T | C | 0.37 | 2.5E-06 | | Pituitary |  | | | | | | | | | | | | | |
|  | | rs555621 |  | *ARL14EP* | T | C | 0.18 | 3.6E-06 | | Thyroid |  | | | | | | | | | | | | | |
|  | | rs555621 |  | *ARL14EP* | T | C | 0.3 | 5.3E-06 | | Brain - Cerebellum |  | | | | | | | | | | | | | |
|  | | rs555621 |  | *FSHB* | T | C | 0.54 | 6.2E-06 | | Brain - Hypothalamus |  | | | | | | | | | | | | | |
|  | | rs555621 |  | *RP4-710M3.1* | T | C | 0.37 | 6.4E-06 | | Testis |  | | | | | | | | | | | | | |
|  | | rs555621 |  | *ARL14EP* | T | C | 0.22 | 0.000025 | | Brain - Caudate (basal ganglia) |  | | | | | | | | | | | | | |
| 11 | | rs11031010 | *FSHB* | *ARL14EP* | C | A | 0.76 | 3.60E-23 | | Testis |  | | | | | | | | | | | | | |
|  | | rs11031010 |  | *ARL14EP* | C | A | 0.42 | 8.70E-15 | | Thyroid |  | | | | | | | | | | | | | |
|  | | rs11031010 |  | *ARL14EP* | C | A | 0.39 | 1.40E-08 | | Nerve - Tibial |  | | | | | | | | | | | | | |
|  | | rs11031010 |  | *ARL14EP* | C | A | 0.3 | 4.70E-08 | | Artery - Aorta |  | | | | | | | | | | | | | |
|  | | rs11031010 |  | *ARL14EP* | C | A | 0.17 | 0.000007 | | Artery - Tibial |  | | | | | | | | | | | | | |
|  | | rs11031010 |  | *ARL14EP* | C | A | 0.21 | 9.5E-06 | | Skin - Sun Exposed (Lower leg) |  | | | | | | | | | | | | | |
|  | | rs11031010 |  | *ARL14EP* | C | A | 0.26 | 0.000018 | | Esophagus - Mucosa |  | | | | | | | | | | | | | |
|  | | rs11031010 |  | *ARL14EP* | C | A | 0.56 | 0.000032 | | Spleen |  | | | | | | | | | | | | | |
|  | | rs11031010 |  | *ARL14EP* | C | A | 0.25 | 0.000033 | | Stomach |  | | | | | | | | | | | | | |
|  | | rs11031010 |  | *ARL14EP* | C | A | 0.26 | 0.000042 | | Adipose - Subcutaneous |  | | | | | | | | | | | | | |
| 11 | | rs1782507 | *FSHB* | *ARL14EP* | G | T | 0.44 | 7.60E-32 | | Esophagus - Muscularis |  | | | | | | | | | | | | | |
|  | | rs1782507 |  | *ARL14EP* | G | T | 0.63 | 9.90E-29 | | Brain - Cerebellum |  | | | | | | | | | | | | | |
|  | | rs1782507 |  | *ARL14EP* | G | T | 0.48 | 1.20E-26 | | Nerve - Tibial |  | | | | | | | | | | | | | |
|  | | rs1782507 |  | *ARL14EP* | G | T | 0.35 | 9.00E-20 | | Thyroid |  | | | | | | | | | | | | | |
|  | | rs1782507 |  | *ARL14EP* | G | T | 0.63 | 1.60E-18 | | Pituitary |  | | | | | | | | | | | | | |
|  | | rs1782507 |  | *ARL14EP* | G | T | 0.55 | 2.50E-16 | | Pancreas |  | | | | | | | | | | | | | |
|  | | rs1782507 |  | *ARL14EP* | G | T | 0.33 | 1.10E-15 | | Esophagus - Mucosa |  | | | | | | | | | | | | | |
|  | | rs1782507 |  | *ARL14EP* | G | T | 0.37 | 1.80E-15 | | Esophagus - Gastroesophageal Junction |  | | | | | | | | | | | | | |
|  | | rs1782507 |  | *ARL14EP* | G | T | 0.3 | 1.40E-14 | | Heart - Atrial Appendage |  | | | | | | | | | | | | | |
|  | | rs1782507 |  | *ARL14EP* | G | T | 0.28 | 4.60E-13 | | Lung |  | | | | | | | | | | | | | |
|  | | rs1782507 |  | *ARL14EP* | G | T | 0.37 | 7.00E-13 | | Colon - Sigmoid |  | | | | | | | | | | | | | |
|  | | rs1782507 |  | *ARL14EP* | G | T | 0.23 | 1.40E-11 | | Skin - Sun Exposed (Lower leg) |  | | | | | | | | | | | | | |
|  | | rs1782507 |  | *ARL14EP* | G | T | 0.38 | 4.20E-10 | | Testis |  | | | | | | | | | | | | | |
|  | | rs1782507 |  | *ARL14EP* | G | T | 0.27 | 7.40E-10 | | Adipose - Subcutaneous |  | | | | | | | | | | | | | |
|  | | rs1782507 |  | *ARL14EP* | G | T | 0.23 | 2.20E-09 | | Skin - Not Sun Exposed (Suprapubic) |  | | | | | | | | | | | | | |
|  | | rs1782507 |  | *ARL14EP* | G | T | 0.38 | 3.40E-09 | | Brain - Cerebellar Hemisphere |  | | | | | | | | | | | | | |
|  | | rs1782507 |  | *ARL14EP* | G | T | 0.25 | 4.70E-09 | | Stomach |  | | | | | | | | | | | | | |
|  | | rs1782507 |  | *ARL14EP* | G | T | 0.25 | 1.30E-08 | | Artery - Aorta |  | | | | | | | | | | | | | |
|  | | rs1782507 |  | *ARL14EP* | G | T | 0.17 | 3.80E-08 | | Heart - Left Ventricle |  | | | | | | | | | | | | | |
|  | | rs1782507 |  | *ARL14EP* | G | T | 0.15 | 1.20E-07 | | Artery - Tibial |  | | | | | | | | | | | | | |
|  | | rs1782507 |  | *ARL14EP* | G | T | 0.37 | 2.70E-07 | | Prostate |  | | | | | | | | | | | | | |
|  | | rs1782507 |  | *ARL14EP* | G | T | 0.43 | 2.90E-07 | | Ovary |  | | | | | | | | | | | | | |
|  | | rs1782507 |  | *ARL14EP* | G | T | 0.27 | 3.10E-07 | | Brain - Caudate (basal ganglia) |  | | | | | | | | | | | | | |
|  | | rs1782507 |  | *ARL14EP* | G | T | 0.29 | 6.10E-07 | | Artery - Coronary |  | | | | | | | | | | | | | |
|  | | rs1782507 |  | *ARL14EP* | G | T | 0.3 | 8.90E-07 | | Adrenal Gland |  | | | | | | | | | | | | | |
|  | | rs1782507 |  | *ARL14EP* | G | T | 0.14 | 1.9E-06 | | Colon - Transverse |  | | | | | | | | | | | | | |
|  | | rs1782507 |  | *ARL14EP* | G | T | 0.27 | 4.4E-06 | | Brain - Cortex |  | | | | | | | | | | | | | |
|  | | rs1782507 |  | *FSHB* | G | T | 0.6 | 0.00001 | | Brain - Hypothalamus |  | | | | | | | | | | | | | |
| 12 | | rs1544410 | *VDR* | *HDAC7* | C | T | 0.14 | 0.000013 | | Esophagus - Mucosa |  | | | | | | | | | | | | | |
| 15 | | rs2241423 | *MAP2K5* | *MAP2K5* | G | A | -0.32 | 4.10E-19 | | Artery - Tibial |  | | | | | | | | | | | | | |
|  | | rs2241423 |  | *MAP2K5* | G | A | -0.4 | 2.90E-15 | | Heart - Atrial Appendage |  | | | | | | | | | | | | | |
|  | | rs2241423 |  | *MAP2K5* | G | A | -0.32 | 1.20E-14 | | Adipose - Subcutaneous |  | | | | | | | | | | | | | |
|  | | rs2241423 |  | *MAP2K5* | G | A | -0.27 | 1.00E-13 | | Thyroid |  | | | | | | | | | | | | | |
|  | | rs2241423 |  | *SKOR1* | G | A | -0.3 | 1.90E-12 | | Lung |  | | | | | | | | | | | | | |
|  | | rs2241423 |  | *SKOR1* | G | A | -0.32 | 3.50E-12 | | Thyroid |  | | | | | | | | | | | | | |
|  | | rs2241423 |  | *SKOR1* | G | A | -0.3 | 1.40E-11 | | Muscle - Skeletal |  | | | | | | | | | | | | | |
|  | | rs2241423 |  | *MAP2K5* | G | A | -0.26 | 1.50E-11 | | Whole Blood |  | | | | | | | | | | | | | |
|  | | rs2241423 |  | *SKOR1* | G | A | -0.4 | 7.30E-11 | | Heart - Left Ventricle |  | | | | | | | | | | | | | |
|  | | rs2241423 |  | *SKOR1* | G | A | -0.29 | 2.10E-10 | | Adipose - Subcutaneous |  | | | | | | | | | | | | | |
|  | | rs2241423 |  | *SKOR1* | G | A | -0.35 | 3.70E-10 | | Adipose - Visceral (Omentum) |  | | | | | | | | | | | | | |
|  | | rs2241423 |  | *MAP2K5* | G | A | -0.28 | 9.20E-10 | | Esophagus - Muscularis |  | | | | | | | | | | | | | |
|  | | rs2241423 |  | *SKOR1* | G | A | -0.26 | 1.40E-09 | | Artery - Tibial |  | | | | | | | | | | | | | |
|  | | rs2241423 |  | *SKOR1* | G | A | -0.23 | 6.20E-09 | | Skin - Sun Exposed (Lower leg) |  | | | | | | | | | | | | | |
|  | | rs2241423 |  | *MAP2K5* | G | A | -0.22 | 9.40E-09 | | Lung |  | | | | | | | | | | | | | |
|  | | rs2241423 |  | *MAP2K5* | G | A | -0.29 | 1.10E-08 | | Artery - Aorta |  | | | | | | | | | | | | | |
|  | | rs2241423 |  | *SKOR1* | G | A | -0.25 | 1.90E-08 | | Skin - Not Sun Exposed (Suprapubic) |  | | | | | | | | | | | | | |
|  | | rs2241423 |  | *MAP2K5* | G | A | -0.27 | 5.40E-08 | | Nerve - Tibial |  | | | | | | | | | | | | | |
|  | | rs2241423 |  | *SKOR1* | G | A | -0.2 | 9.50E-08 | | Nerve - Tibial |  | | | | | | | | | | | | | |
|  | | rs2241423 |  | *SKOR1* | G | A | -0.32 | 1.10E-07 | | Artery - Aorta |  | | | | | | | | | | | | | |
|  | | rs2241423 |  | *MAP2K5* | G | A | -0.27 | 1.20E-07 | | Adipose - Visceral (Omentum) |  | | | | | | | | | | | | | |
|  | | rs2241423 |  | *SKOR1* | G | A | -0.48 | 1.30E-07 | | Pituitary |  | | | | | | | | | | | | | |
|  | | rs2241423 |  | *SKOR1* | G | A | -0.3 | 1.30E-07 | | Breast - Mammary Tissue |  | | | | | | | | | | | | | |
|  | | rs2241423 |  | *SKOR1* | G | A | -0.24 | 1.80E-07 | | Esophagus - Muscularis |  | | | | | | | | | | | | | |
|  | | rs2241423 |  | *MAP2K5* | G | A | -0.25 | 1.90E-07 | | Breast - Mammary Tissue |  | | | | | | | | | | | | | |
|  | | rs2241423 |  | *MAP2K5* | G | A | -0.23 | 9.00E-07 | | Heart - Left Ventricle |  | | | | | | | | | | | | | |
|  | | rs2241423 |  | *SKOR1* | G | A | -0.29 | 6.3E-06 | | Esophagus - Gastroesophageal Junction |  | | | | | | | | | | | | | |
|  | | rs2241423 |  | *SKOR1* | G | A | -0.51 | 0.000011 | | Ovary |  | | | | | | | | | | | | | |
|  | | rs2241423 |  | *SKOR1* | G | A | -0.25 | 0.000012 | | Colon - Sigmoid |  | | | | | | | | | | | | | |
|  | | rs2241423 |  | *MAP2K5* | G | A | -0.27 | 0.000013 | | Esophagus - Gastroesophageal Junction |  | | | | | | | | | | | | | |
|  | | rs2241423 |  | *SKOR1* | G | A | -0.2 | 0.000016 | | Colon - Transverse |  | | | | | | | | | | | | | |
|  | | rs2241423 |  | *MAP2K5* | G | A | -0.38 | 0.000017 | | Pituitary |  | | | | | | | | | | | | | |
|  | | rs2241423 |  | *RP11-34F13.2* | G | A | -0.29 | 0.00004 | | Thyroid |  | | | | | | | | | | | | | |
| 22 | | rs4633 | *COMT* | *COMT* | C | T | 0.3 | | 5.30E-18 | Esophagus - Mucosa |  | | | | | | | | | | | | | |
|  | | rs4633 |  | *ARVCF* | C | T | 0.23 | | 9.80E-15 | Thyroid |  | | | | | | | | | | | | | |
|  | | rs4633 |  | *COMT* | C | T | 0.22 | | 9.60E-11 | Skin - Not Sun Exposed (Suprapubic) |  | | | | | | | | | | | | | |
|  | | rs4633 |  | *ARVCF* | C | T | -0.27 | | 1.20E-07 | Artery - Aorta |  | | | | | | | | | | | | | |
|  | | rs4633 |  | *ARVCF* | C | T | 0.19 | | 3.2E-06 | Esophagus - Mucosa |  | | | | | | | | | | | | | |
|  | | rs4633 |  | *COMT* | C | T | 0.13 | | 0.000029 | Skin - Sun Exposed (Lower leg) |  | | | | | | | | | | | | | |
|  |  | | | | | | | | | | | | | | | | | | | | | | |  |
|  | * ([http://www.gtexportal.org](http://www.gtexportal.org/)) (р <8.0е-5. FDR≤0.05) | | | | | | | | | | |  |  |  |  |  |  |  |  |  |  |  |  | |
