# Supplementary material for: Candidate Genes for Age at Menarche Are Associated With Uterine Leiomyoma
Source: Front Genet. 2021 Jan 22;11:512940. doi: 10.3389/fgene.2020.512940 (PMC7863975; doi:10.3389/fgene.2020.512940)
Supplement: Supplementary file 1 [file Data_Sheet_1.zip › SupMaterial 16-12-2020/Sup_Table_19.DOC]

Supplementary Table 19 Gene set enrichment analysis of biological pathways with 28 UL-associated genes (Gene Ontology (GO) Portal tools - PANTHER Overrepresentation Test accessed on 04/13/2017; PANTHER version 12.0 accessed on 07/10/2017, [http://geneontology.org](http://geneontology.org/)).

| Database | Gene set | Original # genes in gene set | Genes in gene set analyzed by Gene Ontology | Fold Enrichment | P | FDR |
| --- | --- | --- | --- | --- | --- | --- |
| GO biological process complete | follicle-stimulating hormone signaling pathway | 5 | *FSHB, FSHR* | > 100 | 1.31E-07 | 0.002 |
|  | ovulation cycle process | 79 | *FSHB, FSHR,*  *ESR1,* *LHCGR* | 36.74 | 4.93E-06 | 0.019 |
|  | ovarian follicle development | 53 | *FSHB, FSHR,*  *ESR1,* *LHCGR* | 54.76 | 1.09E-06 | 0.008 |
|  | female gonad development | 87 | *FSHB, FSHR,*  *ESR1,* *LHCGR* | 33.36 | 7.12E-06 | 0.018 |
|  | hormone-mediated signaling pathway | 166 | *FSHB, FSHR, ESR1, LHCGR,VDR* | 21.86 | 3.37E-06 | 0.017 |
|  | mammary gland branching involved in pregnancy | 6 | *ESR1,* *VDR* | > 100 | 5.09E-05 | 0.044 |
|  | primary ovarian follicle growth | 3 | *FSHB, FSHR* | > 100 | 1.82E-05 | 0.024 |
|  | regulation of hormone levels | 514 | *FSHB, FSHR,ESR1, LHCGR,VDR, GC, COMT* | 9.88 | 5.28E-06 | 0.016 |
| GO molecular function complete | D3 vitamins binding | 2 | *VDR, GC* | > 100 | 1.10E-05 | 0.050 |
|  | follicle-stimulating hormone receptor activity | 2 | *FSHB, FSHR* | > 100 | 1.10E-05 | 0.025 |
| PANTHER GO-Slim Biological Process | response to endogenous stimulus | 219 | *FSHB, FSHR,*  *LHCGR,VDR, SKOR1* | 16.57 | 1.25E-05 | 0.003 |
| Reactome pathways | hormone ligand-binding receptors | 11 | *FSHB, FSHR,*  *LHCGR* | > 100 | 8.45E-07 | 0.002 |
|  | G alpha (s) signalling events | 141 | *FSHB, FSHR,*  *POMC,* *LHCGR* | 20.58 | 4.47E-05 | 0.044 |
