# Supplementary material for: Candidate Genes for Age at Menarche Are Associated With Uterine Leiomyoma
Source: Front Genet. 2021 Jan 22;11:512940. doi: 10.3389/fgene.2020.512940 (PMC7863975; doi:10.3389/fgene.2020.512940)
Supplement: Supplementary file 1 [file Data_Sheet_1.zip › SupMaterial 16-12-2020/Sup_Table_10.DOC]

Supplementary Table 10 Cross-validation of the significant models of gene-environment interactions associated with uterine leiomyoma.

| N | Models of gene-environment interactions | OR (95%CI) | Test. Bal. Acc. | S_e_ | S_p_ | CVC |
| --- | --- | --- | --- | --- | --- | --- |
| Two-order interaction models | | | | | | |
| 1 | rs12324955 *FTO* x aborts | 3.20 (2.58-3.97) | 64.16 | 63.80 | 64.53 | 10/10 |
| Three-order interaction models | | | | | | |
| 1 | rs11031010 *FSHB* x rs12324955 *FTO* x aborts | 3.52 (2.83-4.37) | 64.06 | 60.11 | 70.03 | 10/10 |
| 2 | rs2164808 *POMC* x chronic endometritis x aborts | 3.50 (2.81-4.35) | 65.13 | 67.66 | 62.59 | 10/10 |
| 3 | rs12324955 *FTO* x chronic endometritis x aborts | 3.56 (2.86-4.42) | 64.78 | 66.26 | 64.42 | 10/10 |
| 4 | rs4374421 *LHCGR* x chronic endometritis x aborts | 3.49 (2.80-4.34) | 65.06 | 68.54 | 61.57 | 10/10 |
| 5 | rs11031010 *FSHB* x rs2164808 *POMC* x aborts | 3.39 (2.73-4.21) | 63.98 | 61.16 | 68.30 | 10/10 |
| 6 | rs12324955 *FTO* x rs4374421 *LHCGR* x aborts | 3.33 (2.67-4.14) | 63.16 | 69.24 | 59.63 | 10/10 |
| Four-order interaction models | | | | | | |
| 1 | rs11031010 *FSHB* x rs12324955 *FTO* x rs314280 *LIN28B* x aborts | 3.77 (3.03-4.68) | 64.18 | 61.86 | 69.93 | 10/10 |
| 2 | rs12324955 *FTO* x rs11031010 *FSHB* x chronic endometritis x aborts | 3.90 (3.14-4.58) | 64.99 | 62.57 | 70.03 | 10/10 |
| 3 | rs11031010 *FSHB* x rs12324955 *FTO* x rs4633 *COMT* x aborts | 3.72 (2.99-4.62) | 62.50 | 62.57 | 69.01 | 10/10 |
| 4 | rs12324955 *FTO* x rs11031010 *FSHB* x rs7753051 *IGF2R* x aborts | 3.62 (2.92-4.50) | 63.10 | 59.75 | 70.95 | 10/10 |
| 5 | rs12324955 *FTO* x rs11031010 *FSHB* x rs2164808 *POMC* x aborts | 3.87 (3.11-4.81) | 64.50 | 65.20 | 67.38 | 10/10 |
| 6 | rs11031010 *FSHB* x rs314276 *LIN28B* x rs2164808 *POMC* x aborts | 3.75 (3.02-4.66) | 63.83 | 60.46 | 71.05 | 10/10 |
| 7 | rs11031010 *FSHB* x rs2164808 *POMC* x rs7759938 *LIN28B* x aborts | 3.70 (2.98-4.60) | 63.21 | 60.98 | 70.34 | 10/10 |
| 8 | rs12324955 *FTO* x rs11031010 *FSHB* x rs4374421 *LHCGR* x aborts | 3.69 (2.97-4.59) | 64.38 | 66.43 | 65.14 | 10/10 |
| 9 | rs12324955 *FTO* x rs4374421 *LHCGR* x chronic endometritis x aborts | 3.74 (2.99-4.67) | 63.49 | 70.83 | 60.65 | 10/10 |

- The results were obtained using the GMDR method with adjustment for сovariates;
- OR (95%CI) – odds ratio at 95% confidence interval, р – significance level.
- Test. Bal. Acc. – Testing balanced accuracy, S_e_ – model sensitivity , S_p_ – model specificity.
- CVC - cross validation consistency,
- all models were validated through the permutation test with adjustment for covariates. The permutation test included 1000 permutations at 10-fold cross-validation that ensured р_рerm_<0.001; the reproducibility of the models (CVC) after the test was 100%.
