# Supplementary material for: Candidate Genes for Age at Menarche Are Associated With Uterine Leiomyoma
Source: Front Genet. 2021 Jan 22;11:512940. doi: 10.3389/fgene.2020.512940 (PMC7863975; doi:10.3389/fgene.2020.512940)
Supplement: Supplementary file 1 [file Data_Sheet_1.zip › SupMaterial 16-12-2020/Sup_Table_11.DOC]

Supplementary Table 11 Associations of the genotype × abortion × chronic endometritis combinations with uterine leiomyoma within the most significant models of gene-environment interactions.

| N  Models | Genotype, aborts and chronic endometritis combinations | Number of cases | Number of controls | | *beta* | P | | Risk,  High/Low |
| --- | --- | --- | --- | --- | --- | --- | --- | --- |
| Two-order interaction models | | | | | | | | |
| 1 | rs12324955 GG x abortion 0 | 91 | | 315 | -0.80 | | 3.56E-09 | L |
|  | rs12324955 GG x abortion 1 | 203 | | 196 | 0.70 | | 5.32E-09 | H |
|  | rs12324955 GA x abortion 1 | 160 | | 152 | 0.68 | | 1.99E-07 | H |
| Three-order interaction models | | | | | | | | |
| 1 | rs11031010 CC x rs12324955 GG x abortion 0 | 71 | | 246 | -0.74 | | 5.58E-07 | L |
|  | rs11031010 CC x rs12324955 GG x abortion 1 | 176 | | 138 | 0.92 | | **2.38E-12** | H |
|  | rs11031010 CC x rs12324955 GA x abortion 1 | 120 | | 113 | 0.64 | | 1.17E-05 | H |
|  | rs11031010 CA x rs12324955 GA x abortion 1 | 38 | | 35 | 0.60 | | 1.43E-02 | H |
| 2 | rs2164808 GG x chronic endometritis 0 x abortion 0 | 49 | | 174 | -0.74 | | 1.83E-05 | L |
|  | rs2164808 GA x chronic endometritis 0 x abortion 0 | 83 | | 290 | -0.80 | | 8.04E-09 | L |
|  | rs2164808 GG x chronic endometritis 0 x abortion 1 | 88 | | 80 | 0.64 | | 1.11E-04 | H |
|  | rs2164808 GA x chronic endometritis 0 x abortion 1 | 189 | | 182 | 0.70 | | 9.66E-09 | H |
|  | rs2164808 AA x chronic endometritis 0 x abortion 1 | 72 | | 81 | 0.44 | | 1.11E-02 | H |
|  | rs2164808 GG x chronic endometritis 1 x abortion 1 | 11 | | 6 | 1.05 | | 4.15E-02 | H |
| 3 | rs12324955 GG x chronic endometritis 0 x abortion 0 | 88 | | 311 | -0.82 | | 1.95E-09 | L |
|  | rs12324955 GA x chronic endometritis 0 x abortion 0 | 71 | | 231 | -0.73 | | 1.18E-06 | L |
|  | rs12324955 GA x chronic endometritis 1 x abortion 0 | 6 | | 1 | 2.45 | | 2.43E-02 | H |
|  | rs12324955 GG x chronic endometritis 0 x abortion 1 | 183 | | 162 | 0.81 | | 1.37E-10 | H |
|  | rs12324955 GA x chronic endometritis 0 x abortion 1 | 144 | | 132 | 0.70 | | 2.18E-07 | H |
| 4 | rs4374421 TT x chronic endometritis 0 x abortion 0 | 87 | | 282 | -0.70 | | 4.14E-07 | L |
|  | rs4374421 TC x chronic endometritis 0 x abortion 0 | 64 | | 237 | -0.82 | | 9.57E-08 | L |
|  | rs4374421 TT x chronic endometritis 0 x abortion 1 | 197 | | 158 | 0.95 | | **2.78E-14** | H |
|  | rs4374421 TC x chronic endometritis 0 x abortion 1 | 124 | | 153 | 0.32 | | 1.70E-02 | H |
| 5 | rs11031010 CC x rs2164808 GG x abortion 0 | 38 | | 142 | -0.77 | | 0.00006 | L |
|  | rs11031010 CC x rs2164808 GA x abortion 0 | 71 | | 227 | -0.65 | | 0.00001 | L |
|  | rs11031010 CA x rs2164808 GA x abortion 0 | 16 | | 65 | -0.84 | | 0.003 | L |
|  | rs11031010 CC x rs2164808 GG x abortion 1 | 72 | | 71 | 0.54 | | 0.002 | H |
|  | rs11031010 CA x rs2164808 GG x abortion 1 | 24 | | 15 | 0.96 | | 0.004 | H |
|  | rs11031010 CC x rs2164808 GA x abortion 1 | 176 | | 163 | 0.72 | | 1.40E-08 | H |
|  | rs11031010 CC x rs2164808 AA x abortion 1 | 69 | | 57 | 0.74 | | 0.00008 | H |
| 6 | rs12324955 GG x rs4374421 TT x abortion 0 | 48 | | 163 | -0.67 | | 1.23E-04 | L |
|  | rs12324955 GA x rs4374421 TT x abortion 0 | 36 | | 114 | -0.61 | | 2.38E-03 | L |
|  | rs12324955 GG x rs4374421 TC x abortion 0 | 34 | | 119 | -0.66 | | 1.21E-03 | L |
|  | rs12324955 GA x rs4374421 TC x abortion 0 | 30 | | 99 | -0.64 | | 3.29E-03 | L |
|  | rs12324955 GG x rs4374421 TT x abortion 1 | 112 | | 87 | 0.84 | | 7.22E-08 | H |
|  | rs12324955 GA x rs4374421 TT x abortion 1 | 88 | | 65 | 0.90 | | 2.87E-07 | H |
| Four-order interaction models | | | | | | | | |
| 1 | rs11031010 CC x rs12324955 GG x rs314280 CT x abortion 0 | 30 | | 126 | -0.87 | | 4.03E-05 | L |
|  | rs11031010 CA x rs12324955 GG x rs314280 CT x abortion 0 | 6 | | 40 | -1.31 | | 3.15E-03 | L |
|  | rs11031010 CC x rs12324955 GG x rs314280 CC x abortion 1 | 66 | | 49 | 0.84 | | 2.22E-05 | H |
|  | rs11031010 CC x rs12324955 GA x rs314280 CC x abortion 1 | 44 | | 40 | 0.65 | | 4.17E-03 | H |
|  | rs11031010 CC x rs12324955 GG x rs314280 CT x abortion 1 | 82 | | 68 | 0.72 | | 4.04E-05 | H |
|  | rs11031010 CC x rs12324955 GA x rs314280 CT x abortion 1 | 60 | | 60 | 0.50 | | 9.73E-03 | H |
|  | rs11031010 CC x rs12324955 GG x rs314280 TT x abortion 1 | 28 | | 21 | 0.81 | | 6.29E-03 | H |
| 2 | rs12324955 GG x rs11031010 CC x chronic endometritis 0 x abortion 0 | 68 | | 244 | -0.78 | | 2.12E-07 | L |
|  | rs12324955 GG x rs11031010 CC x chronic endometritis 0 x abortion 1 | 158 | | 115 | 0.99 | | **5.75E-13** | H |
|  | rs12324955 GA x rs11031010 CC x chronic endometritis 0 x abortion 1 | 109 | | 98 | 0.68 | | 7.14E-06 | H |
|  | rs12324955 GA x rs11031010 CA x chronic endometritis 0 x abortion 1 | 33 | | 30 | 0.60 | | 2.06E-02 | H |
| 3 | rs11031010 CC x rs12324955 GG x rs4633 TC x abortion 0 | 33 | | 119 | -0.69 | | 8.71E-04 | L |
|  | rs11031010 CC x rs12324955 GA x rs4633 TC x abortion 0 | 25 | | 92 | -0.76 | | 1.11E-03 | L |
|  | rs11031010 CC x rs12324955 GG x rs4633 CC x abortion 0 | 13 | | 64 | -1.04 | | 8.65E-04 | L |
|  | rs11031010 CC x rs12324955 GG x rs4633 TC x abortion 1 | 91 | | 65 | 0.89 | | 2.63E-07 | H |
|  | rs11031010 CC x rs12324955 GA x rs4633 TC x abortion 1 | 70 | | 57 | 0.72 | | 1.42E-04 | H |
|  | rs11031010 CA x rs12324955 GA x rs4633 TC x abortion 1 | 20 | | 9 | 1.31 | | 1.37E-03 | H |
|  | rs11031010 CC x rs12324955 GG x rs4633 CC x abortion 1 | 45 | | 30 | 0.92 | | 1.74E-04 | H |
|  | rs11031010 CC x rs12324955 GA x rs4633 CC x abortion 1 | 31 | | 27 | 0.67 | | 1.41E-02 | H |
| 4 | rs12324955 GG x rs11031010 CC x rs7753051 TT x abortion 0 | 40 | | 118 | -0.45 | | 2.00E-02 | L |
|  | rs12324955 GG x rs11031010 CA x rs7753051 TT x abortion 0 | 5 | | 32 | -1.26 | | 9.51E-03 | L |
|  | rs12324955 GG x rs11031010 CC x rs7753051 TC x abortion 0 | 25 | | 112 | -0.98 | | 2.21E-05 | L |
|  | rs12324955 GG x rs11031010 CC x rs7753051 TT x abortion 1 | 96 | | 63 | 0.99 | | 1.06E-08 | H |
|  | rs12324955 GA x rs11031010 CC x rs7753051 TT x abortion 1 | 65 | | 45 | 0.89 | | 1.20E-05 | H |
|  | rs12324955 GA x rs11031010 CA x rs7753051 TC x abortion 1 | 18 | | 12 | 0.94 | | 1.35E-02 | H |
|  | rs12324955 GG x rs11031010 CC x rs7753051 CC x abortion 1 | 19 | | 11 | 1.05 | | 6.37E-03 | H |
| 5 | rs12324955 GG x rs11031010 CC x rs2164808 GG x abortion 0 | 19 | | 79 | -0.81 | | 2.03E-03 | L |
|  | rs12324955 GA x rs11031010 CC x rs2164808 GA x abortion 0 | 30 | | 94 | -0.58 | | 7.85E-03 | L |
|  | rs12324955 GG x rs11031010 CA x rs2164808 GA x abortion 0 | 7 | | 41 | -1.19 | | 4.32E-03 | L |
|  | rs12324955 GG x rs11031010 CC x rs2164808 GG x abortion 1 | 37 | | 24 | 0.92 | | 6.76E-04 | H |
|  | rs12324955 GA x rs11031010 CA x rs2164808 GG x abortion 1 | 14 | | 7 | 1.19 | | 1.14E-02 | H |
|  | rs12324955 GG x rs11031010 CC x rs2164808 GA x abortion 1 | 98 | | 83 | 0.72 | | 7.84E-06 | H |
|  | rs12324955 GA x rs11031010 CC x rs2164808 GA x abortion 1 | 71 | | 59 | 0.73 | | 9.83E-05 | H |
|  | rs12324955 GG x rs11031010 CC x rs2164808 AA x abortion 1 | 41 | | 31 | 0.82 | | 8.11E-04 | H |
|  | rs12324955 GA x rs11031010 CC x rs2164808 AA x abortion 1 | 21 | | 16 | 0.76 | | 2.60E-02 | H |
| 6 | rs11031010 CC x rs314276 CA x rs2164808 GG x abortion 0 | 9 | | 65 | -1.37 | | 1.52E-04 | L |
|  | rs11031010 CA x rs314276 CC x rs2164808 GA x abortion 0 | 9 | | 36 | -0.83 | | 2.98E-02 | L |
|  | rs11031010 CC x rs314276 CA x rs2164808 GA x abortion 0 | 26 | | 107 | -0.87 | | 1.37E-04 | L |
|  | rs11031010 CC x rs314276 CC x rs2164808 GG x abortion 1 | 47 | | 32 | 0.91 | | 1.32E-04 | H |
|  | rs11031010 CC x rs314276 CC x rs2164808 GA x abortion 1 | 87 | | 78 | 0.69 | | 4.38E-05 | H |
|  | rs11031010 CC x rs314276 CA x rs2164808 GA x abortion 1 | 79 | | 64 | 0.72 | | 5.72E-05 | H |
|  | rs11031010 CC x rs314276 CC x rs2164808 AA x abortion 1 | 34 | | 32 | 0.56 | | 2.71E-02 | H |
|  | rs11031010 CC x rs314276 CA x rs2164808 AA x abortion 1 | 29 | | 20 | 0.92 | | 1.94E-03 | H |
| 7 | rs11031010 CC x rs2164808 GG x rs7759938 TC x abortion 0 | 10 | | 63 | -1.21 | | 4.67E-04 | L |
|  | rs11031010 CC x rs2164808 GA x rs7759938 TC x abortion 0 | 27 | | 112 | -0.88 | | 7.58E-05 | L |
|  | rs11031010 CC x rs2164808 GG x rs7759938 TT x abortion 1 | 46 | | 32 | 0.90 | | 1.77E-04 | H |
|  | rs11031010 CA x rs2164808 GG x rs7759938 TT x abortion 1 | 15 | | 8 | 1.09 | | 1.46E-02 | H |
|  | rs11031010 CC x rs2164808 GA x rs7759938 TT x abortion 1 | 96 | | 82 | 0.73 | | 6.48E-06 | H |
|  | rs11031010 CC x rs2164808 AA x rs7759938 TT x abortion 1 | 38 | | 35 | 0.60 | | 1.45E-02 | H |
|  | rs11031010 CC x rs2164808 GA x rs7759938 TC x abortion 1 | 72 | | 64 | 0.62 | | 6.39E-04 | H |
|  | rs11031010 CC x rs2164808 AA x rs7759938 TC x abortion 1 | 25 | | 18 | 0.87 | | 5.75E-03 | H |
| 8 | rs12324955 GG x rs11031010 CC x rs4374421 TT x abortion 0 | 37 | | 134 | -0.72 | | 2.19E-04 | L |
|  | rs12324955 GG x rs11031010 CA x rs4374421 TC x abortion 0 | 8 | | 36 | -0.89 | | 2.51E-02 | L |
|  | rs12324955 GG x rs11031010 CC x rs4374421 TT x abortion 1 | 96 | | 63 | 1.00 | | 8.01E-09 | H |
|  | rs12324955 GA x rs11031010 CC x rs4374421 TT x abortion 1 | 68 | | 46 | 0.97 | | 1.46E-06 | H |
|  | rs12324955 GG x rs11031010 CC x rs4374421 TC x abortion 1 | 62 | | 58 | 0.59 | | 2.37E-03 | H |
| 9 | rs12324955 GG x rs4374421 TT x chronic endometritis 0 x abortion 0 | 47 | | 159 | -0.67 | | 1.57E-04 | L |
|  | rs12324955 GG x rs4374421 TC x chronic endometritis 0 x abortion 0 | 32 | | 119 | -0.72 | | 5.29E-04 | L |
|  | rs12324955 GG x rs4374421 TT x chronic endometritis 0 x abortion 1 | 101 | | 67 | 1.03 | | 1.49E-09 | H |
|  | rs12324955 GA x rs4374421 TT x chronic endometritis 0 x abortion 1 | 76 | | 57 | 0.87 | | 2.57E-06 | H |
|  | rs12324955 GG x rs4374421 TC x chronic endometritis 0 x abortion 1 | 66 | | 73 | 0.42 | | 1.93E-02 | H |
|  | rs12324955 GA x rs4374421 CC x chronic endometritis 0 x abortion 1 | 12 | | 7 | 1.01 | | 3.63E-02 | H |

The results were obtained by the MB-MDR analysis with adjustment for сovariates;

*beta* – coefficient of logistic regression;

1 - abortion is present, 0 – abortion is absent;

1 - chronic endometritis is present, 0 – chronic endometritis is absent
