# Supplementary material for: Candidate Genes for Age at Menarche Are Associated With Uterine Leiomyoma
Source: Front Genet. 2021 Jan 22;11:512940. doi: 10.3389/fgene.2020.512940 (PMC7863975; doi:10.3389/fgene.2020.512940)
Supplement: Supplementary file 1 [file Data_Sheet_1.zip › SupMaterial 16-12-2020/Sup_Table_15.DOC]

Supplementary Table 15 Effect of the UL-associated SNPs on the gene expression (eQTL) in peripheral blood according to the Blood eQTL browser.*

| Chr | SNP | Gene/Region | MAF | Gene Expressed | Mechanism | Z-score | P | FDR |
| --- | --- | --- | --- | --- | --- | --- | --- | --- |
| 2 | rs2164808 | *POMC* | T | *DNAJC27* | Cis | -4.31 | 1.7*10^-5^ | 0.01 |
| 2 | rs7589318 | *POMC* | A | *DNAJC27* | Cis | -4.94 | 7.8*10^-7^ | 0 |
| 6 | rs7766109 | *F13A1* | G | *F13A1* | Cis | -5.48 | 4.4*10^-8^ | 0 |
| 6 | rs7753051 | *IGF2R* | C | *SLC22A1* | Cis | -5.06 | 4.2*10^-7^ | 0 |
| 9 | rs10980926 | *ZNF483* | A | *KIAA0368* | Cis | -6.02 | 1.7*10^-9^ | 0 |
| 11 | rs11031010 | *FSHB* | A | *C11orf46* | Cis | -11.51 | 1.1*10^-30^ | 0 |
| 15 | rs2241423 | *MAP2K5* | A | *MAP2K5* | Cis | -10.37 | 3.3*10^-25^ | 0 |
| 22 | rs4633 | *COMT* | C | *COMT* | Cis | -4.14 | 3.4*10^-5^ | 0.02 |

* http://genenetwork.nl/bloodeqtlbrowser/ (р <5.0е-5. FDR<0.05)
