# Supplementary material for: Candidate Genes for Age at Menarche Are Associated With Uterine Leiomyoma
Source: Front Genet. 2021 Jan 22;11:512940. doi: 10.3389/fgene.2020.512940 (PMC7863975; doi:10.3389/fgene.2020.512940)
Supplement: Supplementary file 1 [file Data_Sheet_1.zip › SupMaterial 16-12-2020/+References to Supplementary Table 4.DOC]

**References to Supplementary Table 4**

Abadi, A., Peralta-Romero, J., Suarez, F., Gomez-Zamudio, J., Burguete-Garcia, A.I., Cruz, M., et al. (2016) Assessing the effects of 35 European-derived BMI-associated SNPs in Mexican children. Obesity (Silver Spring) 9, 1989-1995.

Berndt, S.I., Gustafsson, S., Mägi, R., Ganna, A., Wheeler, E., Feitosa, M.F., et al. (2013). Justice AE, Monda KL, Croteau-Chonka DC, Day FR et al. Genome-wide meta-analysis identifies 11 new loci for anthropometric traits and provides insights into genetic architecture. *Nat. Genet.* 5, 501-512.

Carroll, J., Saxena, R., Welt, C.K. (2012) Environmental and genetic factors influence age at menarche in women with polycystic ovary syndrome. *J. Pediatr. Endocrinol. Metab.* 5-6, 459-466.

Carty, C.L., Spencer, K.L., Setiawan, V.W., Fernandez-Rhodes, L., Malinowski, J., Buyske, S., et al. (2013). Replication of genetic loci for ages at menarche and menopause in the multi-ethnic Population Architecture using Genomics and Epidemiology (PAGE) study. *Hum. Reprod.* 6, 1695-1706.

Chen, C.T., Fernández-Rhodes, L., Brzyski, R.G., Carlson, C.S., Chen, Z., Heiss, G., et al. (2012). Replication of loci influencing ages at menarche and menopause in Hispanic women: the Women’s Health Initiative SHARe Study. *Hum. Mol. Genet.* 6, 1419-1432.

Chenthuran, T., Galhenagey, G.H., Jayasekara, R.W., Dissanayake, V.H. (2014) Polymorphism in the epidermal growth factor gene is associated with pre-eclampsia and low birthweight. *J. Obstet. Gynaecol. Res.* 5, 1235-1242.

Cobayashi, F., Lourenço, B.H., Cardoso, M.A. (2015). 25-Hydroxyvitamin D3 levels, BsmI polymorphism and insulin resistance in Brazilian Amazonian children. *Int. J. Mol. Sci.* 6, 12531-12546.

Cotsapas, C., Speliotes, E.K., Hatoum, I.J., Greenawalt, D.M., Dobrin, R., Lum, P.Y., et al. (2009). Common body mass index-associated variants confer risk of extreme obesity. *Hum. Mol. Genet.* 18, 3502-3507.

Cousminer, D.L., Berry, D.J., Timpson, N.J., Ang, W., Thiering, E., Byrne, E.M., et al. (2013). Genome-wide association and longitudinal analyses reveal genetic loci linking pubertal height growth, pubertal timing and childhood adiposity. *Hum. Mol. Genet.* 13, 2735-2747.

Cousminer, D.L., Stergiakouli, E., Berry, D.J., Ang, W., Groen-Blokhuis, M.M., Körner, A., et al. (2014). Genome-wide association study of sexual maturation in males and females highlights a role for body mass and menarche loci in male puberty. *Hum. Mol. Genet.* 16, 4452-4464.

Dahlman, I., Vaxillaire, M., Nilsson, M., Lecoeur, C., Gu, H.F., Cavalcanti-Proença, C., et al. (2008). Estrogen receptor alpha gene variants associate with type 2 diabetes and fasting plasma glucose. *Pharmacogenet. Genomics*, 11, 967-975.

Demerath, E.W., Liu, C.T., Franceschini, N., Chen, G., Palmer, J.R., Smith, E.N., et al. (2013). Genome-wide association study of age at menarche in African-American women. Hum. Mol. Genet. 16, 3329-3346.

Delahanty, R.J., Beeghly-Fadiel, A., Long, J.R., Gao, Y.T., Lu, W., Xiang, Y.B., et al. (2013). Evaluation of GWAS-identified genetic variants for age at menarche among Chinese women. *Hum. Reprod.* 4, 1135-43.

Doo, M., Kim, Y. (2011). Association between ESR1 rs1884051 polymorphism and dietary total energy and plant protein intake on obesity in Korean men. *Nutr. Res. Pract.* 6, 527-532.

Du, J., Wang, Z., Zhang, J., Jia, L., Zhang, F., Shi, Y., et al. (2014). Association between single nucleotide polymorphism of rs2252673 of INSR gene and polycystic ovarian syndrome. *Zhonghua Fu Chan Ke Za Zhi* 12, 919-924.

Duicu, C., Mărginean, C.O., Voidăzan, S., Tripon, F., Bănescu, C. (2016). FTO rs9939609 SNP is associated with adiponectin and leptin levels and the risk of obesity in a cohort of Romanian children population. *Medicine (Baltimore)* 20, e3709.

Elks, C.E., Perry, J.RB., Sulem, P., Chasman, D.I., Franceschini, N., He, C., et al. (2010). Thirty new loci for age at menarche identified by a meta-analysis of genome-wide association studies. *Nat. Genet.* 12, 1077-1085.

Engelman, C.D., Fingerlin, T.E., Langefeld, C.D.,  Hicks, P.J., Rich, S.S., Wagenknecht, L.E., et al. (2008). Genetic and environmental determinants of 25-hydroxyvitamin D and 1,25-dihydroxyvitamin D levels in Hispanic and African Americans. *J. Clinl. Endocrinol. Metab.* 9, 3381-3388.

Fernandez-Rhodes, L., Demerath, E.W., Cousminer, D.L., Tao, R., Dreyfus, J.G., Esko, T., et al. (2018). Association of adiposity genetic variants with menarche timing in 92,105 women of European descent. *Am. J. Epidemiol.* (2013) 3, 451-460.

Frayling, T.M., Timpson, N.J., Weedon, M.N., Zeggini, E., Freathy, R.M., Lindgren, C.M., et al. (2007). A common variant in the FTO gene is associated with body mass index and predisposes to childhood and adult obesity. *Science* 5826, 889-94.

Graff, M., Ngwa, J.S., Workalemahu, T., Homuth, G., Schipf, S., Teumer, A. et al. (2013). Genome–wide analysis of BMI in adolescents and young adults reveals additional insight into the effects of genetic loci over the life course. *Hum. Mol. Genet.* 22(17), 3597–3607.

Goodarzi, M.O., Louwers, Y.V., Taylor, K.D., Jones, M.R., Cui, J., Kwon, S., et al. (2011). Replication of association of a novel insulin receptor gene polymorphism with polycystic ovary syndrome. *Fertil. Steril.* 5, 1736-41.e11.

He, C., Kraft, P., Chen, C., Buring, J.E., Paré, G., Hankinson, S.E., et al. (2009). Genome-wide association studies identify novel loci associated with age at menarche and age at natural menopause. *Nat. Genet.* 6, 724-728.

He, C., Kraft, P., Buring, J.E., Chen, C., Hankison, S.E., Pare, G., et al. (2010). A large-scale candidate-gene association study of age at menarche and age at natural menopause. *Hum. Genet.* 5, 515-527.

Hong, K.W., Oh, B. (2012). Recapitulation of genome-wide association studies on body mass index in the Korean population. Int. J. Obes. (Lond). 8, 1127-1130.

Hsiao, T.J., Lin, E. (2016). Association of a common rs9939609 variant in the fat mass and obesity-associated (FTO) gene with obesity and metabolic phenotypes in a Taiwanese population: a replication study. *J. Genet.* 3, 595-601.

Jia, F., Sun, R.F., Li, Q.H., Wang, D.X., Zhao, F., Li, J.M., et al. (2013). Vitamin D receptor BsmI polymorphism and osteoporosis risk: a meta-analysis from 26 studies. *Genet. Test. Mol. Biomarkers*, 1, 30-34.

Kitanaka, S., Isojima, T., Takaki, M., Numakura, C., Hayasaka, K., Igarashi, T. (2012). Association of vitamin D-related gene polymorphisms with manifestation of vitamin D deficiency in children. *Endocr. J.* 11, 1007-1014.

Laczmanski, L., Lwow, F., Mossakowska, M., Puzianowska-Kuznicka, M., Szwed, M., Kolackov, K., et al. (2015). Association between vitamin D concentration and levels of sex hormones in an elderly Polish population with different genotypes of VDR polymorphisms (rs10735810, rs1544410, rs7975232, rs731236). *Gene* 1, 73-76.

Lango Allen, H., Estrada, K., Lettre, G., Berndt, S.I., Weedon, M.N., Rivadeneira, F., et al. (2010). Hundreds of variants clustered in genomic loci and biological pathways affect human height. *Nature* 7317, 832-838.

Leinonen, J.T., Surakka, I., Havulinna, A.S., Kettunen, J., Luoto, R., Salomaa, V., et al. (2012). Association of LIN28B with Adult Adiposity-Related Traits in Females. *PLoS One* 11, e48785.

Lv, D., Zhang, D.D., Wang, H., Zhang, Y., Liang, L., Fu, J.F., et al. (2015). Genetic variations in SEC16B, MC4R, MAP2K5 and KCTD15 were associated with childhood obesity and interacted with dietary behaviors in Chinese school-age population. *Gene* 2, 149-155.

Mei, H., Chen, W., Jiang, F., He, J., Srinivasan, S., Smith, E.N., et al. (2012). Longitudinal replication studies of GWAS risk SNPs influencing body mass index over the course of childhood and adulthood. *PLoS One* 2, e31470.

Ong, K.K., Elks, C.E., Li, S., Zhao, J.H., Luan, J., Andersen, B., et al. (2009). Genetic variation in LIN28B is associated with the timing of puberty. *Nat. Genet.* 6, 729-733.

Petkeviciene, J., Smalinskiene, A., Klumbiene, J., Petkevicius, V., Kriaucioniene, V., Lesauskaite, V. (2016). Physical activity, but not dietary intake, attenuates the effect of the FTO rs9939609 polymorphism on obesity and metabolic syndrome in Lithuanian adult population. *Public. Health.* 135, 23-29.

Perry, J.R., Stolk, L., Franceschini, N., Lunetta, K.L., Zhai, G., McArdle, P.F., et al. (2009). Meta-analysis of genome-wide association data identifies two loci influencing age at menarche. *Nat. Genet.* 6, 648-650.

Perry, J.R., Voight, B.F., Yengo, L., Amin, N., Dupuis, J., Ganser, M., et al. (2012). Stratifying type 2 diabetes cases by BMI identifies genetic risk variants in LAMA1 and enrichment for risk variants in lean compared to obese cases. *PLoS Genet.* 5, e1002741.

Perry, J.R., Day, F., Elks, C.E., Sulem, P., Thompson, D.J., Ferreira, T., et al. (2014). Parent-of-origin-specific allelic associations among 106 genomic loci for age at menarche. *Nature* 7520, 92-97.

Pyun, J.A., Kim, S., Cho, N.H., Koh, I., Lee, J.Y., Shin, C., et al. (2014). Genome-wide association studies and epistasis analyses of candidate genes related to age at menarche and age at natural menopause in a Korean population. *Menopause* 5, 522-529.

Ponomarenko, I.V., Reshetnikov, E.A., Altuchova, O.B., Polonikov, A.V., Sorokina, I.N., Yermachenko, A., et al. (2019). Association of genetic polymorphisms with age at menarche in Russian women. *Gene* 686, 228-236. https://doi.org/10.1016/j.gene. (2018).11.042.

Quan, L.L., Wang, H., Tian, Y., Mu, X., Zhang, Y., Tao, K. (2015). Association of fat-mass and obesity-associated gene FTO rs9939609 polymorphism with the risk of obesity among children and adolescents: a meta-analysis. *Eur. Rev. Med. Pharmacol. Sci.* 4, 614-623.

Rask-Andersen, M., Jacobsson, J.A., Moschonis, G., Ek, A.E., Chrousos, G.P., Marcus, C., et al. (2012). The MAP2K5-linked SNP rs2241423 is associated with BMI and obesity in two cohorts of Swedish and Greek children. *BMC Med. Genet.* 13, 36.

Schweighofer, N., Lerchbaum, E., Trummer, O., Schwetz, V., Pilz, S., Pieber, T.R., et al. (2012). Androgen levels and metabolic parameters are associated with a genetic variant of F13A1 in women with polycystic ovary syndrome. *Gene* 1, 133-139.

Speliotes, E.K., Willer, C.J., Berndt, S.I., Monda, K.L., Thorleifsson, G., Jackson, A.U., et al. (2010). Association analyses of 249,796 individuals reveal 18 new loci associated with body mass index. *Nat. Genet.* 11, 937-948.

Stavrou, I., Zois, C., Chatzikyriakidou, A., Georgiou, I., Tsatsoulis, A. (2006). Combined estrogen receptor α and estrogen receptor β genotypes influence the age of menarche. *Hum. Reprod.* 2, 554-557.

Suh, K.T., Eun, I.S., Lee, J.S. (2010). Polymorphism in vitamin D receptor is associated with bone mineral density in patients with adolescent idiopathic scoliosis. *Eur. Spine. J.* 9, 1545-1550.

Sulem, P., Gudbjartsson, D.F, Rafnar, T., Holm, H., Olafsdottir, E.J., Olafsdottir, G.H., et al. (2009). Genome-wide association study identifies sequence variants on 6q21 associated with age at menarche. *Nat. Genet.* 6, 734-738.

Tian, Y., Zhao, H., Chen, H., Peng, Y., Cui, L., Du, Y., et al. (2016). Variants in FSHB are associated with polycystic ovary syndrome and luteinizing hormone level in Han Chinese women. *J. Clin. Endocrinol. Metab.* 101(5), 2178–2184.

Tu, W., Wagner, E.K., Eckert, G.J., Yu, Z., Hannon, T., Pratt, J.H., et al. (2015). Associations between menarche-related genetic variants and pubertal growth in male and female adolescents. *J. Adolesc. Health.* 1, 66-72.

Wang, J.T., Guo, Y., Yang, T.L., Xu, X.H., Dong, S.S., Li, M., et al. (2008). Polymorphisms in the estrogen receptor genes are associated with hip fractures in Chinese. *Bone* 5, 910-914.

Wang, H.J., Hinney, A., Song, J.Y., Scherag, A., Meng, X.R., Grallert, H., et al. (2016). Association of common variants identified by recent genome-wide association studies with obesity in Chinese children: a case-control study. *BMC Med. Genet.* 17, 7.

Widén, E., Ripatti, S., Cousminer, D.L., Surakka, I., Lappalainen, T., Järvelin, M.R., et al. (2010). Distinct variants at LIN28B influence growth in height from birth to adulthood. *Am. J. Hum. Genet.* 5, 773-782.

Willer, C.J., Schmidt, E.M., Sengupta, S., Peloso, G.M., Gustafsson, S., Kanoni, S., et al. (2013). Discovery and refinement of loci associated with lipid levels. *Nat. Genet.* 11, 1274-1283.

Xu, X.H., Xiong, D.H., Liu, X.G., Guo, Y., Chen, Y., Zhao, J., et al. (2010). Association analyses of vitamin D-binding protein gene with compression strength index variation in Caucasian nuclear families. *Osteoporos. Int.* 1, 99-107.

Yang, T.L., Guo, Y., Li, S.M., Li, S.K., Tian, Q., Liu, Y.J., et al. (2013). Ethnic differentiation of copy number variation on chromosome 16p12.3 for association with obesity phenotypes in European and Chinese populations. *Int J Obes (Lond)* 2, 188-190.

Yermachenko, A., Dvornyk, V. (2016). UGT2B4 previously implicated in the risk of breast cancer is associated with menarche timing in Ukrainian females. *Gene* 1, 85-89.

Zhang, Y., Wang, X., Liu, Y., Qu, H., Qu, S., Wang, W., et al. (2012). The GC, CYP2R1 and DHCR7 genes are associated with vitamin D levels in northeastern Han Chinese children. *Swiss. Med. Wkly.* 142, w13636.
