# Supplementary material for: Candidate Genes for Age at Menarche Are Associated With Uterine Leiomyoma
Source: Front Genet. 2021 Jan 22;11:512940. doi: 10.3389/fgene.2020.512940 (PMC7863975; doi:10.3389/fgene.2020.512940)
Supplement: Supplementary file 1 [file Data_Sheet_1.zip › SupMaterial 16-12-2020/Sup_Table_7.DOC]

Supplementary Table 7 Associations of haplotypes with uterine leiomyoma.

| Gene | Haploblock | SNPs included | Haplotype | Frequency | | OR | P |
| --- | --- | --- | --- | --- | --- | --- | --- |
|  |  |  |  | uterine leiomyoma (n=569) | control (n=981) |  |  |
| *POMC* | H1 | rs2164808\|rs7589318 | AA | 0.308 | 0.305 | 1.01 | 0.905 |
|  |  | rs2164808\|rs7589318 | AG | 0.173 | 0.164 | 1.05 | 0.622 |
|  |  | rs2164808\|rs7589318 | GG | 0.519 | 0.531 | 0.96 | 0.629 |
| *LHCGR* | H2 | rs7579411\|rs6729809\|rs4953616 | TCC | 0.277 | 0.277 | 0.97 | 0.712 |
|  |  | rs7579411\|rs6729809\|rs4953616 | TCT | 0.034 | 0.044 | 0.76 | 0.194 |
|  |  | rs7579411\|rs6729809\|rs4953616 | TTT | 0.113 | 0.131 | 0.89 | 0.372 |
|  |  | rs7579411\|rs6729809\|rs4953616 | CTT | 0.576 | 0.549 | 1.09 | 0.281 |
| *FSHR* | H3 | rs6732220\|rs4953655 | GG | 0.229 | 0.247 | 0.90 | 0.283 |
|  |  | rs6732220\|rs4953655 | GA | 0.012 | 0.010 | 1.38 | 0.379 |
|  |  | rs6732220\|rs4953655 | CA | 0.758 | 0.743 | 1.09 | 0.354 |
| *UGT2B4* | H4 | rs2013573\|rs13111134 | AA | 0.181 | 0.188 | 0.93 | 0.513 |
|  |  | rs2013573\|rs13111134 | GA | 0.047 | 0.044 | 1.16 | 0.439 |
|  |  | rs2013573\|rs13111134 | GG | 0.772 | 0.768 | 1.02 | 0.840 |
| *LIN28B* | H5 | rs4946651\|rs7759938 | AC | 0.258 | 0.300 | 0.81 | 0.019 |
|  |  | rs4946651\|rs7759938 | AT | 0.134 | 0.126 | 1.11 | 0.357 |
|  |  | rs4946651\|rs7759938 | GT | 0.608 | 0.574 | 1.12 | 0.147 |
| *ESR1* | H6 | rs3020394\|rs1884051 | GG | 0.295 | 0.291 | 0.97 | 0.711 |
|  |  | rs3020394\|rs1884051 | AA | 0.705 | 0.709 | 1.04 | 0.634 |
| *ZNF483* | H7 | rs10980926\|rs10441737 | AC | 0.312 | 0.332 | 0.88 | 0.166 |
|  |  | rs10980926\|rs10441737 | GT | 0.688 | 0.668 | 1.12 | 0.190 |
| *FSHB* | H8 | rs555621\|rs11031010\|rs1782507 | ACC | 0.381 | 0.348 | 1.13 | 0.148 |
|  |  | rs555621\|rs11031010\|rs1782507 | GAA | 0.107 | 0.131 | 0.74 | 0.014 |
|  |  | rs555621\|rs11031010\|rs1782507 | GCA | 0.278 | 0.277 | 1.09 | 0.342 |
|  |  | rs555621\|rs11031010\|rs1782507 | ACA | 0.235 | 0.245 | 0.94 | 0.505 |

The results were obtained by the logistic regression analysis with adjustment for covariates;

OR – odds ratio, р – significance level.
