# Supplementary material for: Candidate Genes for Age at Menarche Are Associated With Uterine Leiomyoma
Source: Front Genet. 2021 Jan 22;11:512940. doi: 10.3389/fgene.2020.512940 (PMC7863975; doi:10.3389/fgene.2020.512940)
Supplement: Supplementary file 1 [file Data_Sheet_1.zip › SupMaterial 16-12-2020/Sup_Table_4.DOC]

Supplementary Table 4 The literature data about associations of the studied polymorphisms with age at menarche and some menarche-related traits.

| Chr | SNP | Gene/Region | Phenotype | Association (significance)  (associated allele/genotype) | Reference |
| --- | --- | --- | --- | --- | --- |
| 1 | rs1514175 | [*TNNI3K*](https://www.ncbi.nlm.nih.gov/entrez/query.fcgi?db=gene&cmd=Retrieve&dopt=Graphics&list_uids=100526835) | Age at menarche  **Body mass index**  **Body mass index**  Body mass index | −12.2 d (p=4.0 × 10^−7^) (A)  **0.07** **kg/m^2^ (p=8 x10^-14^) (А)**  **0.06 unit (p=3 x10^-11^) (А)**  0.20 kg/m^2^ (p=7.7× 10^−4^) (A) | (Fernandez-Rhodes et al., 2013)  **(Spelioteset al., 2010)**  **(Graff al., 2013)**  (Hong al., 2012) |
| 1 | rs466639 | [*RXRG*](https://www.ncbi.nlm.nih.gov/entrez/query.fcgi?db=gene&cmd=Retrieve&dopt=Graphics&list_uids=6258) | **Age at menarche**  Body mass index  Age at menarche  **Age at menarche** | **-4.2 w (р= 1 x10^-13^) (T)**  increasing (р= 0.011) (T)  -4.1 w (р= 0.023) (T)  **0.08 unit (p= 2 x10^-24^) (C)** | **(Elks al., 2010)**  (Elks al., 2010)  (Delahanty al., 2013)  **(Perry al., 2014)** |
| 1 | rs7538038 | [*KISS1*](https://www.ncbi.nlm.nih.gov/entrez/query.fcgi?db=gene&cmd=Retrieve&dopt=Graphics&list_uids=3814) | Age at menarche | -0.061 y (р_adj_=0.012) (G) | (He al., 2010) |
| 2 | rs713586 | *RBJ* | Age at menarche  **Body mass index** | 11.7 d (р=8.4 × 10^−7^) (T)  **0.14 kg/m^2^ (p= 6 x10^-22^) (С)** | (Fernandez-Rhodes et al., 2013)  **(Spelioteset al., 2013)** |
| 2 | rs2164808 | *POMC* | Age at menarche | 0.046 y (р_adj_=0.038) (T) | (He al., 2010) |
| 2 | rs7589318 | *POMC* | Age at menarche | 0.048 y (р_adj_=0.047)(A) | (He al., 2010) |
| 2 | rs4374421 | [*LHCGR*](https://www.ncbi.nlm.nih.gov/entrez/query.fcgi?db=gene&cmd=Retrieve&dopt=Graphics&list_uids=3973) | Age at menarche | 0.060 y (р_adj_=0.017) (C) | (He al., 2010) |
| 2 | rs7579411 | [*LHCGR*](https://www.ncbi.nlm.nih.gov/entrez/query.fcgi?db=gene&cmd=Retrieve&dopt=Graphics&list_uids=3973) | Age at menarche | 0.058 y (р_adj_=0.013)(T) | (He al., 2010) |
| 2 | rs6729809 | [*LHCGR*](https://www.ncbi.nlm.nih.gov/entrez/query.fcgi?db=gene&cmd=Retrieve&dopt=Graphics&list_uids=3973) | Age at menarche | 0.063 y (р_adj_=0.0099) (C) | (He al., 2010) |
| 2 | rs4953616 | [*LHCGR*](https://www.ncbi.nlm.nih.gov/entrez/query.fcgi?db=gene&cmd=Retrieve&dopt=Graphics&list_uids=3973) | Age at menarche | -0.065 y (р_adj_=0.006) (T) | (He al., 2010) |
| 2 | rs6732220 | [*FSHR*](https://www.ncbi.nlm.nih.gov/entrez/query.fcgi?db=gene&cmd=Retrieve&dopt=Graphics&list_uids=2492) | Age at menarche  Age at menarche | 0.17 y (р=0.0018,p_bonf_>0.05)(G) *  0.34 y (p= 3 x10^-5^) epistasis | (Pyun al., 2014) |
| 2 | rs4953655 | [*FSHR*](https://www.ncbi.nlm.nih.gov/entrez/query.fcgi?db=gene&cmd=Retrieve&dopt=Graphics&list_uids=2492) | Age at menarche  Age at menarche | 0.17 y (р=0.0023,p_bonf_>0.05)(G) *  0.34 y (p= 3 x10^-5^) epistasis | (Pyun al., 2014) |
| 2 | rs887912 | *FANCL* | Age at menarche  **Body mass index**  **Obesity (class I)**  **Obesity (class II)** | −11.1 d (р=3.8 × 10^−5^) (T)  **0.1 kg/m^2^ (p= 2 x10^-12^) (T)**  **OR= 1.07 (p= 1 x10^-10^ ) (T)**  **OR= 1.1 (p= 6 x10^-9^ ) (T)** | (Fernandez-Rhodes et al., 2013)  **(Spelioteset al., 2013)**  **(Berndt al., 2013)**  **(Berndt al., 2013)** |
| 2 | rs12617311 | *PLCL1* | **Age at menarche**  Age at menarche | **-3.0 w (р= 6 x10^-13^) (A**)  -4.7 w (р=0.004) (A) | **(Elks al., 2010)**  (Delahanty al., 2013) |
| 3 | rs6438424 | *3q13.32* | **Age at menarche**  Height adult  Age at menarche  Age at menarche | **-2.7 w (р= 1 x10^-13^) (A)**  -0.013 Zscore (p=0.0004) (A)  -3.7 w (р=0.008) (A))  0.17 y (р_perm_=0.005) (A)) | **(Elks al., 2010)**  (Elks al., 2010)  (Delahanty al., 2013)  (Ponomarenko al., 2019) |
| 4 | rs2013573 | [***UGT2B4***](https://www.ncbi.nlm.nih.gov/entrez/query.fcgi?db=gene&cmd=Retrieve&dopt=Graphics&list_uids=7363) | Age at menarche | 0.060 y (р_adj_=0.019) (C) | (He al., 2010) |
| 4 | rs13111134 | *UGT2B4* | Age at menarche  Age at menarche | 0.055 y (р_adj_=0.042) (G)  0.431y (p_bonf_=0.044) (A) | (He al., 2010)  (Yermachenko al., 2016) |
| 4 | rs222003 | *GC* | Plasma levels of 25(OH)D and 1,25(OH)_2_D  Compression strength index (male) | (p>0.05)  (p>0.05) | (Engelman al., 2008)  (Xu al., 2010) |
| 4 | rs222020 | *GC* | Plasma 25(OH)D concentrations  Compression strength index (male)  Adolescent idiopathic scoliosis | (p=0.004)  (p=0.0042)  (p<0.05) | (Zhang al., 2012)  (Xu al., 2010)  (Wang al., 2016) |
| 4 | rs3756261 | *EGF* | Birth weight | lower (p<0.0001) (A)  (4 snps haplotype) | (Chenthuran al., 2014) |
| 5 | rs757647 | *KDM3B* | **Age at menarche**  Age at menarche | **-2.4w(р=5.4x10^-8^,р_bonf_>0.05)(A)***  -4.2 w (р=0.003) (A) | **(Elks al., 2010)**  (Delahanty al., 2013) |
| 6 | rs7766109 | *F13A1* | Body mass index,  pressure insulin response,  triglycerides,  high density lipoprotein cholesterol in PCOS | (p=0.013)  (p=0.015)  (p=0.001)  (p=0.012) | (Schweighofer al., 2012) |
| 6 | rs4946651 | *LIN28B* | **Age at menarche** | **0.085 y (p= 3 x10^-8^) (A)** | **(He al., 2009)** |
| 6 | rs7759938 | *LIN28B* | **Age at menarche**  **Age at menarche**  Height adult  Age at menarche  [**Height**](https://www.ebi.ac.uk/gwas/search?query=Height)  **Late pubertal growth**  **Age at menarche**  Tanner stage  Tanner stage (boys)  Tanner stage (girls)  Height adult  Height adult  Pubertal growth  Age at menarche in PCOS | **0.09 y (p= 7 x10^-9^) (C)**  **6.4 w (p= 5 x10^-60^) (C)**  -0.042 Zscore (p=8.7 x10^-18^) (T)  8.3 w (p= 4 x10^-6^) (C)  **-0.045 unit (p= 8 x10^-31^) (T)**  **0.11 unit (p= 4 x10^-9^) (C)**  **0.12 unit (p= 8 x10^-110^) (C)**  ‐0.080 (p=3.42 x10^-8^) (C)  ‐0.063 (p=0.012) (C)  ‐0.088 (p=5.57 x10^-7^) (C)  increasing (p=8.69 x10^-18^) (C)  0.042 cm (p= 2 x10^-6^) (C)  (p=5x10^-11^)  earlier (p=0.006) (T) | **(Perry al., 2009)**  **(Elks al., 2010)**  (Elks al., 2010)  (Delahanty al., 2013)  **(Lango al., 2010)**  **(Cousminer al., 2013)**  **(Perry al., 2014)**  (Perry al., 2014)  (Perry al., 2014)  (Perry al., 2014)  (Perry al., 2014)  (Leinonen al., 2012)  (Widén al., 2010)  (Carroll al., 2012) |
| 6 | rs314280 | *LIN28B* | **Age at menarche**  **Age at menarche**  Age at menarche (EA)  Age at menarche (Asians) | **0.086 y (p= 2 x10^-8^) (T)**  **1.2 m (p=2 x10^-14^) (T)**  0.08 y (p=2 x10^-8^) (T)  0.13 y (p= 0.0012) (T) | **(He al., 2009)**  **(Sulem al., 2009)**  (Carty al., 2013)  (Carty al., 2013) |
| 6 | rs314276 | *LIN28B* | **Age at menarche**  Height  Breast development in girls  Voice breaking  Height growth in girls  [**Puberty onset**](https://www.ebi.ac.uk/gwas/search?query=Puberty onset) | **- 0.14 y (р= 4 x10^-16^) (C)**  -0.37 cm (p=3.6 x10^-7^) (C)  earlier (p = 0.001)  earlier (p = 0.006)  faster tempo (p= 0.00008)  **0.08 unit (p= 2 x10^-8^ ) (C)** | **(Ong al., 2009)**  (Ong al., 2009)  **(Cousminer al., 2014)** |
| 6 | rs3020394 | *ESR1* | Age at menarche  Hip fractures | -0.12 y (p=0.0081,p_bonf_>0.05)(A)*  OR=1.66 (p=0.0004) | (Pyun al., 2014)  (Wang al., 2008) |
| 6 | rs1884051 | *ESR1* | Age at menarche  Hip fractures  Body weight,  body mass index ,  waist-hip ratio,  fat body mass,  body fat percentage  obesity in men  Type 2 diabetes | -0.13 y (p=0.0035,p_bonf_>0.05)(A)*  OR=1.46 (p=0.0004)  lower (p = 0.007) (A),  (p = 0.003) (A) ,  (p= 0.011) (A) ,  (p= 0.010) (A),  (p= 0.040) (A),  decreased (p=0.04) (A)  (p=0.011) | (Pyun al., 2014)  (Wang al., 2008)  (Doo al., 2011)  (Dahlman al., 2008) |
| 6 | rs7753051 | *IGF2R* | Age at menarche  Age at menarche  Age at menarche | 0.14 y (p=0.001, p_bonf_>0.05) (T) *  0.34 y (p= 3 x10^-5^) epistasis  0.500 y (p=0.046,p_bonf_>0.05)(A)* | (Pyun al., 2014)  (Pyun al., 2014)  (Yermachenko al., 2016) |
| 7 | rs1079866 | *INHBA* | **Age at menarche**  Height adult  **Age at menarche**  Tanner stage  Tanner stage (girls)  Height adult  Age at menarche | **3.9 w (p=6 x10^-14^) (G)**  -0.016 Zscore (p=0.015) (C)  **0.07 unit (p=9 x10^-24^) (G )**  ‐0.054 (p=0.005) (G)  ‐0.058 (p=0.015) (G)  increasing (p=0.015) (G)  0.13 y (p=0.037) (G) | **(Elks al., 2010)**  (Elks al., 2010)  **(Perry al., 2014)**  (Perry al., 2014)  (Perry al., 2014)  (Perry al., 2014)  (Chen al., 2012) |
| 8 | rs2288696 | *FGFR1* | Age at menarche | -0.058 y (p_adj_=0.033) (G) | (He al., 2010) |
| 9 | rs2090409 | *TMEM38B* | **Age at menarche**  **Age at menarche**  Height adult  Age at menarche | **-0.1 y (p= 2 x10^-9^) (A)**  **-4.7 w (p= 2 x10^-33^) (A)**  -0.020 Zscore (p=2.8 x10^-5^) (A)  -3.7 w (р=0.006) (A) | **(Perry al., 2009)**  **(Elks al., 2010)**  (Elks al., 2010)  (Delahanty al., 2013) |
| 9 | rs10980926 | *ZNF483* | **Age at menarche**  Height adult  Age at menarche | **2.5 w (p= 4 x10^-11^) ( A)**  -0.015 Zscore (p=0.0017) (G)  2.6 w (p= 0.047) ( A) | **(Elks al., 2010)**  (Elks al., 2010)  (Delahanty al., 2013) |
| 9 | rs10441737 | *ZNF483* | **Age at menarche (EA)** | **-2.78 unit (p= 4 x10^-15^) (T)** | **(Demerath al., 2013)** |
| 11 | rs10769908 | *STK33* | Age at menarche | 9.6 d (p=7.8× 10^-5^) (T) | (Fernandez-Rhodes et al., 2013) |
| 11 | rs555621 | *FSHB* | Age at menarche | 0.059 y (p_adj_=0.0011) (C) | (He al., 2010) |
| 11 | rs11031010 | *FSHB* | Age at menarche  PCOS | 0.079 y (p_adj_=0.0037) (A)  (p=4.27 × 10^-8^ ) | (He al., 2010)  (Tian al., 2016) |
| 11 | rs1782507 | *FSHB* | Age at menarche | 0.046 y (p_adj_=0.021) (T) | (He al., 2010) |
| 11 | rs6589964 | *BSX* | **Age at menarche**  Height adult | **-2.7 w (p= 2 x10^-12^) (A)**  -0.014 Zscore (p=0.0025) (A) | **(Elks al., 2010)**  (Elks al., 2010) |
| 12 | rs1544410 | *VDR* | Vitamin D deficiency in children  Vitamin D vs testosterone level, free androgen index in women  23% of the variation in serum 25-hydroxyvitamin D concentrations in children  Testosterone level and free androgen index in women  Osteoporosis  Bone mineral density at the lumbar spine in girls with adolescent idiopathic scoliosis  Adolescent idiopathic scoliosis | OR=2.33 (p=0.041) (G)  (p<0.05)  (p<0.05)  p<0.002  OR=0.61-0.70 (p<0.05)  lower levels (p<0.05) (TT)  (p<0.05) | (Kitanaka al., 2012)  (Laczmanski al., 2015)  (Cobayashi al., 2015)  (Laczmanski al., 2015)  (Jia al., 2013)  (Suh al., 2010)  (Wang al., 2016) |
| 14 | rs999460 | *NKX2-1* | Age at menarche | 0.046 y (p_adj_=0.039) (T) | (He al., 2010) |
| 14 | rs4986938 | *ESR2* | Age at menarche | -7.0 m (р=0.005) (G) | (Stavrou al., 2006) |
| 15 | rs2241423 | *MAP2K5* | Age at menarche  **Body mass index**  Obesity  Body mass index  Children obesity  Obesity  Adulthood body mass index  Body mass index | 13.1 d (p=6.1× 10^-6^) (A)  **0.13 kg/m^2^ (p= 1 x10^-18^) (G)**  OR = 0.79 (p= 0.029) (A)  −0.092 unit (p = 0.028) (A)  (p<0.005)  OR=1.34 (0.001) (G)  (p<0.05)  0.11 kg/m^2^ (p=0.028) (G) | (Fernandez-Rhodes et al., 2013)  **(Spelioteset al., 2013)**  (Rask-Andersen al., 2012)  (Rask-Andersen al., 2012)  (Lv al., 2015)  (Wang al., 2016)  (Mei al., 2012)  (Hong al., 2012) |
| 16 | rs12444979 | *GPRC5B* | Age at menarche  **Body mass index**  Body mass index  Body mass index (EA)  Body fat mass (EA) | 13.5 d (p=2.6× 10^-4^) (T)  **0.17 kg/m^2^ (p=3 x10^-21^) (C)**  (p<0.05)  (p=0.014)  (p=0.002) | (Fernandez-Rhodes et al., 2013)  **(Spelioteset al., 2013)**  (Abadi al., 2012)  (Yang al., 2013)  (Yang al., 2013) |
| 16 | rs9939609 | [*FTO*](https://www.ncbi.nlm.nih.gov/entrez/query.fcgi?db=gene&cmd=Retrieve&dopt=Graphics&list_uids=79068) | **Age at menarche**  Body mass index  **Body mass index**  **Body mass index**  **Obesity**  Obesity  Obesity  Obesity  Obesity  Body mass index  Extreme obesity | **-2.1 w (p= 3 x10^-8^) (A)**  increasing (р= 6.3 x10^-17^) (A)  **0.33 kg/m^2^ (p= 4 x10^-51^) (A)**  **0.36 kg/m^2^ (p= 3 x10^-35^) (A)**  **OR=1.25 (p= 1 x10^-20^) (A)**  OR=9.86 (p=0.026) (AA)  OR=2.03 (p = 0.002) (AA)  OR=1.72 (p=0.009) (AA)  OR = 1.39 (p < 0.01) (A)  increasing (p=0.0059) (A)  OR=1.46 (p=1.42 x10^-11^) (A) | **(Elks al., 2010)**  (Elks al., 2010)  **(Willer al., 2013)**  **(Frayling al., 2007)**  **(Perry al., 2012)**  (Hsiao al., 2016)  (Duicu al., 2016)  (Petkeviciene al., 2016)  (Quan al., 2015)  (Tu al., 2015)  (Cotsapas al., 2009) |
| 16 | rs12324955 | [*FTO*](https://www.ncbi.nlm.nih.gov/entrez/query.fcgi?db=gene&cmd=Retrieve&dopt=Graphics&list_uids=79068) | Age at menarche | -0.15 y (p=0.0057,p_bonf_>0.05)(A)* | (Pyun al., 2014) |
| 18 | rs1398217 | [*SKOR2*](https://www.ncbi.nlm.nih.gov/entrez/query.fcgi?db=gene&cmd=Retrieve&dopt=Graphics&list_uids=652991) | **Age at menarche** | **-2.7 w (р= 2 x10^-13^) (G)** | **(Elks al., 2010)** |
| 19 | rs2252673 | [*INSR*](https://www.ncbi.nlm.nih.gov/entrez/query.fcgi?db=gene&cmd=Retrieve&dopt=Graphics&list_uids=3643) | PCOS  PCOS | OR=1.32 (p=0.006) (G)  (p<0.05) | (Goodarzi al., 2011)  (Du al., 2011) |
| 20 | rs1073768 | *GHRH* | Age at menarche | -0.046 y (p_adj_=0.045) (C) | (He al., 2010) |
| 22 | rs4633 | [*COMT*](https://www.ncbi.nlm.nih.gov/entrez/query.fcgi?db=gene&cmd=Retrieve&dopt=Graphics&list_uids=1312) | Age at menarche | 0.13 y (p=0.0097,p_bonf_>0.05) (T) * | (Pyun al., 2014) |
| X | rs5930973 | [*CD40LG*](https://www.ncbi.nlm.nih.gov/entrez/query.fcgi?db=gene&cmd=Retrieve&dopt=Graphics&list_uids=959) | Age at menarche | 0.101 y (p_adj_=0.0067) (A) | (He al., 2010) |
| X | rs3092921 | [*CD40LG*](https://www.ncbi.nlm.nih.gov/entrez/query.fcgi?db=gene&cmd=Retrieve&dopt=Graphics&list_uids=959) | Age at menarche | 0.074 y (p_adj_=0.037) (C) | (He al., 2010) |

*Note:* The data from the GWAS are highlighted in bold.

* putative candidate loci age at menarche (crude p<0.05, corrected for multiple comparisons p_bonf_>0.05).
